# Identification of stable housekeeping genes in response to ionizing radiation in cancer research

**DOI:** 10.1038/srep43763

**Published:** 2017-03-06

**Authors:** Gopal Iyer, Albert R. Wang, Sean R. Brennan, Shay Bourgeois, Eric Armstrong, Pari Shah, Paul M. Harari

**Affiliations:** 1 Department of Human Oncology and the University of Wisconsin School of Medicine and Public Health, University of Wisconsin-Madison, Madison, USA

## Abstract

Housekeeping genes (HKGs) are essential for basic maintenance of a variety of cellular processes. They ideally maintain uniform expression independent of experimental conditions. However, the effects of ionizing radiation (IR) on HKG expression is unclear. Statistical algorithms, geNorm and Normfinder were used for estimating the stability of HKGs as raw quantification cycle (Cq) values were not a reliable factor for normalization. Head and neck, non-small lung and pancreas cells were exposed to 2, 4 and 6 Gy IR doses and expression of fourteen HKGs was measured at 5 min to 48 h post-irradiation within a given tissue. Paired and single cell line analyses under these experimental conditions identified TATA-Box Binding Protein (TBP) and Importin 8 (IPO8) to be stable in non-small cell lung cancer. In addition to these two genes, Ubiquitin C (UBC) in head and neck cancer and Transferrin receptor (TFRC) and β-Glucuronidase (GUSB) in pancreatic cancer were identified to be stable as well. In summary we present a resource for top ranked five stable HKGs and their transcriptional behavior in commonly used cancer model cell lines and suggest the use of multiple HKGs under radiation treatment conditions is a reliable metric for quantifying gene expression.

Gene expression changes, following exposure to stimuli, contribute to the molecular phenotype of a cell. For such changes to be measured accurately it is imperative to have a faithful set of genes that remain relatively constant under stimulated conditions. The expression of housekeeping genes (HKGs) is assumed to be constant across various cellular and developmental processes. However, there is increasing evidence that HKGs are involved in maintaining cellular structure and homeostasis under various experimental conditions^1^,^2^,^3^ and therefore their utility as normalizing factors can be compromised when reporting differences in gene expression. For example, a classic HKG like GAPDH has been reported to have variable expression across tissues^4^. Among experimental perturbations, one of the more extreme stressors of cellular processes is ionizing radiation. Ionizing radiation (IR) has system wide effects on normal and cancer cells^5^: at the molecular level-DNA damage^6^ alteration in oxidative stress^7^ and signal transduction pathways^8^; at the cellular level, arrest in cell cycle progression^9^ and apoptosis^10^ and at the organismal level, acute radiation sickness, central nervous system damage and carcinogenesis^11^ can be observed. Hence, due to its wide ranging global effects, measuring the altered gene expression profiles is critical for understanding the complexity of cancer in response to IR.

For this study, we adopted one of the most sensitive and powerful medium throughput platforms to measure specific changes in gene expression under various experimental conditions - quantitative real time polymerase chain reaction (qRT-PCR). The aim of the study was to identify a set of stably expressed HKGs across time post-radiation in paired cell lines - head and neck cancer – SCC6, SCC-1483; non-small lung cancer –A549, NCI-H226 and pancreas cancer – MIA PaCa-2, PANC-1. To determine the stability of HKG within a given tissue, qPCR data of HKGs from the same tissue (for e.g., SCC6 and SCC-1483 for head and neck cancer) were consolidated and put into four groups based on radiation doses: Untreated, 2, 4 and 6 Gy. Each group contained all time points (5 min, 1, 5, 24 and 48 h) within that given dose and the two algorithms were performed on each group individually.

We chose fourteen HKGs (Supplementary Table S1) which reflect a wide variety of basic cellular pathways in normal cells. We ruled out ribosomal genes 5 S and 18 S as it is transcribed by RNA Polymerase III and Polymerase I respectively while selected HKGs gene in this study are transcribed by RNA Polymerase II. Second, the range of Cq values of several HKGs in this study are broad which would allow for accurate normalization of target genes and control for potential outliers during any experimental perturbation. Finally, the number of HKGs were selected to encompass various biological functions required for basic maintenance of the cell.

This comprehensive approach was employed to reduce the bias that may occur when only one stable HKG is used as a reference for a given IR dose. We applied two independent and rigorous statistical methods geNorm and Normfinder algorithms to estimate the stability of these HKGs.

Across all doses, HKGs that were consistently ranked within top five stable genes by independent statistical algorithms, geNorm and NormFinder would be considered as stable HKGs. We used both the algorithms as geNorm relies on multiple pair-wise correlation and the need for large sample size is not absolutely essential for calculating the stability of HKGs^12^ while the strength of Normfinder is dependent on the sample size and is biased towards genes that have similar (Cq) values. We implemented the algorithms but distinguish our experimental design in that we introduced 2 independent variables – five time points and three radiation doses for a given tissue. We addressed two main questions in this study: a) The effect of clinically relevant radiation doses on the stability of the 14 different HKGs within a given tissue and b) effect on the stability of these HKGs post-irradiation, up to 48 h within a given tissue. By the definition of an ideal housekeeping gene, it should have stable expression between samples taken from different experimental conditions. However, radiation can induce DNA damage in human cells, and resulting changes in gene expression over time as part of the cells complex response^13^. In addition, changes could also include commonly used housekeeping genes, and thus making them unstable over the time period after being exposed to radiation^14^.

To investigate the utility of our approach in the context of IR, we normalized the expression of EGFR using three stable and unstable HKGs and in addition calculated the geometric mean along with the traditional arithmetic mean for accurate averaging of the expression across all IR doses in head and neck cancer cell lines. To our knowledge, this study is unique as we measured the expression and stability of fourteen HKGs in six independent cancer cell lines exposed to IR.

## Results

### The study design

Fourteen HKGs primers were designed using the PrimerQuest software and optimized for an annealing temperature of 61 °C. A single annealing temperature was important to reduce inter and intra-variability that could potentially arise during amplification of different samples (Supplementary Table S1). The experimental matrix contained 14 HKGs X 3 independent biological replicates X 3 technical replicates per gene X 3 IR doses X 5 time intervals X 2 cell lines per cancer with corresponding non-irradiated controls. Head and neck cancer cell lines - SCC6 and SCC-1483 (squamous cell carcinoma), non-small lung cancer – A549 and NCI-H226 and pancreas cancer – MIA PaCa-2 and PANC-1 were chosen in this study for irradiation as they are commonly used in molecular and biochemical experiments. The treatment doses were chosen to be clinically relevant and the time points were chosen to determine the range from immediate (5 minute) to long-term effects (48 hours). The aim of the study was to identify a set of stably expressed genes across time post-irradiation dose of 2, 4 and 6 Gy in head and neck, non-small lung and pancreas tissue. To determine the stability of HKGs within a given tissue, qPCR data of cells from the same were consolidated and put into four groups based on radiation doses: Untreated, 2, 4 and 6 Gy. Each group contains all time points (5 min, 1, 5, 24 and 48 h) within the given dose and GeNorm and NormFinder were performed on each group individually.

### Expression levels of housekeeping genes across different IR doses

**Figure 1:**
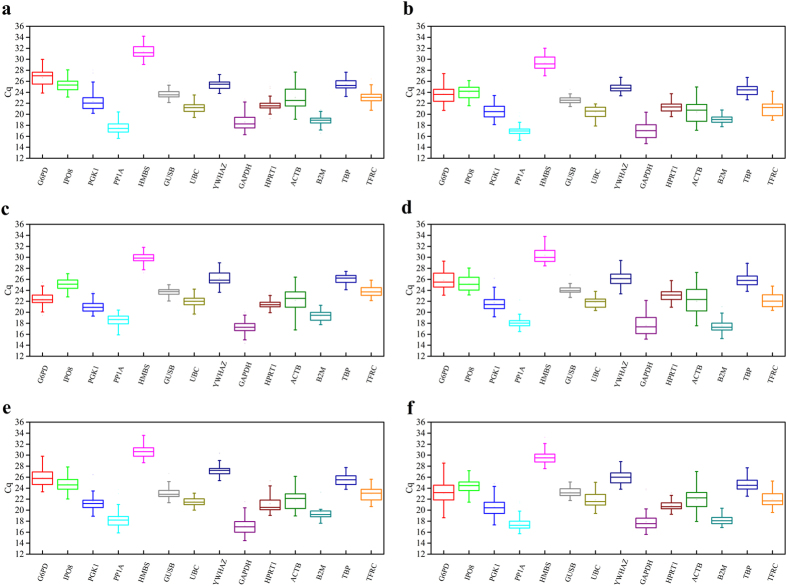
RNA expression of fourteen housekeeping genes in irradiated cancer cell lines. Values are given as cycle threshold numbers (C_q_). Each subplot shows the HKG expression of the following cells: (**a**) SCC6, (**b**) SCC-1483, (**c**) A549, (**d**) NCI-H226, (**e**) MIA PaCa-2, and (**f**) PANC-1. Data from all treated conditions (radiation doses and time) were included for each cell. The boxes represent the lower and upper quartiles with lines in between representing medians; whiskers represent the range of data from nine technical triplicates from three biological replicates.

The relative abundance of the 14 HKGs across all IR treatment groups with corresponding non-irradiated controls was determined by direct cycle threshold (Cq) method (Supplementary Tables S2, S3, S4, S5, S6 and S7). The interquartile range estimated under IR doses of 2, 4 and 6 Gy revealed considerable variation in expression among the 14 HKGs in various cancer cell lines (Fig. 1a–f).

Interestingly, β-Actin (ACTB) had the maximum interquartile range amongst all cell lines across all IR treatment groups while the minimum range in SCC6 (Fig. 1a), SCC-1483 (Fig. 1b), A549 (Fig. 1c), NCI-H226 (Fig. 1d), MIA PaCa-2 (Fig. 1e) and PANC-1 (Fig. 1f) cell lines were 0.858 for hypoxanthine phosphoribosyltransferase 1 (HPRT1); 0.809 for peptidylprolyl isomerase A (PP1A); 0.815, 0.781for β-glucuronidase (GUSB); 0.965 for tyrosine 3-monooxygenase (YWHAZ) and 1.128 for B2M respectively. In contrast, in the non-irradiated controls, the interquartile range did not reveal any consensus HKGs. However, the minimum and maximum (min-max) range for head and neck cancer cell line SCC6 was calculated to be 0.490 for HPRT1 and 2.990 for ACTB (Supplementary Fig. S1a) while for SCC-1483 (Supplementary Fig. S1b) it was 0.409 for B2M and 4.093 for ACTB respectively. In non-small lung cancer cell lines A549 (Supplementary Fig. S1c) and NCI-H226 (Supplementary Fig. S1d), the min-max range was determined to be 0.234 for HPRT1; 0.112 for B2M and 2.157 for ACTB and 1.905 for GAPDH respectively. Likewise, in pancreatic cell lines, MIA PaCa-2 (Supplementary Fig. S1e) and PANC-1 (Supplementary Fig. S1f), the min-max range was 0.249 for G6PD; 0.331 for B2M and 1.804 for YWHAZ and 2.607 for ACTB respectively.

Furthermore, we observed that radiation doses modulated the mean Cq values suggesting either specificity of cancer cell type or experimental variation. To explore either of the experimental observations, analyses of Tables S2–S7 and S14 (unirradiated) revealed variations in mean Cq values. For example, G6PD in head and neck cancer cell lines SCC-6 and SCC1483 was modulated by radiation doses compared to untreated. In SCC-6, unirradiated had a Cq value of 25.99 while radiation doses 2, 4 and 6 Gy had 27, 26.51 and 26.51 respectively. Similarly, in SCC-1483, untreated – 24.04 while radiation doses 2, 4 and 6 Gy was 24.04, 23.2 and 23.63). These variations are not surprising because each cell type, albeit, from the same tissue have been isolated at different stages of cancer suggesting the complexity of the radiation effects on molecular expression of HKGs. However, the experimental variation is minimal or negligible because the statistical values obtained from the mean Cq for each gene is calculated from 45 independent measurements. All raw (Cq) values are provided in Supplementary Tables S8–S13.

Since the comparison of the raw Cq values using the box plots are not a reliable assessment of expression stability, we decided to use the geNorm and Normfinder algorithms to estimate the stability of HKGs on non-normalized expression Cq levels.

### Stability of housekeeping genes across radiation dose and time

Stable HKGs show relatively uniform mean Cq across all time points, while the unstable HKGs demonstrated significantly larger variation from the mean Cq (Supplementary Tables S2–S7). For example, one of the stable HKGs for SCC-1483 is TBP, and the mean Cq of TBP ranged from 23.58 to 25.53, a difference of 1.95 cycles. By comparison, a less stable HKG of SCC-1483 such as ACTB, demonstrated a mean Cq range from 19.50 to 22.94 (difference of 3.44 cycles). To address this variable expression in HKGs, Cq values obtained across all dose treatments and time intervals (Supplementary Tables S2–S7) for individual cancer cell lines were used for stability calculations by applying the geNorm algorithm^15^. The geNorm algorithm defines the gene stability value, M, as the average pairwise variation between a given gene and all other HKGs by iteratively computing their expression values to exclude unstable genes. This step-wise approach eventually results in the lowest M value for the most stable HKG while the gene with the highest M value is excluded from the analyses. We applied this step-wise ranking for the top five genes with respect to their stability in head and neck cancer: SCC6, SCC-1483; non-small lung – A549, NCI-H226 and pancreas – MIA PaCa-2, PANC-1 cell lines and binned them into three IR dose treatment groups of 2, 4 and 6 Gy.

### Stability of HKGs in head and neck cancer

**Figure 2:**
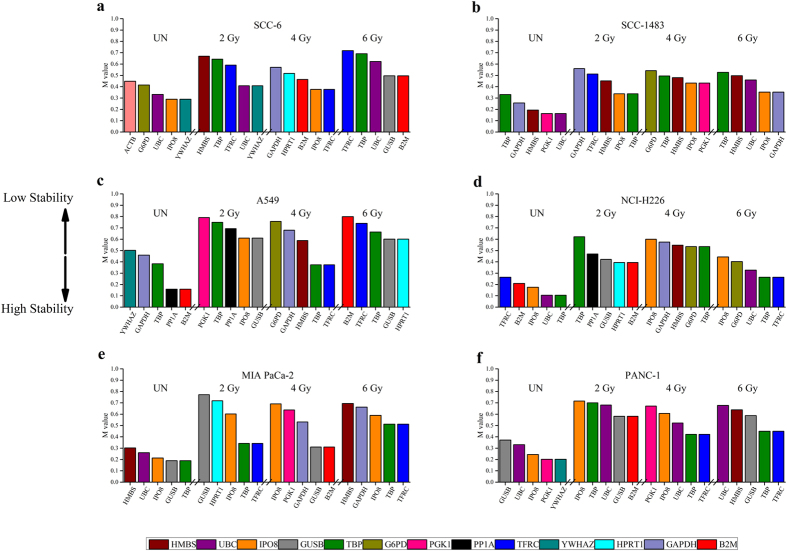
Top five housekeeping gene stability measure M by geNorm for all irradiated cancer cell lines with untreated control. (**a**) SCC6, (**b**) SCC-1483, (**c**) A549, (**d**) NCI-H226, (**e**) MIA PaCa-2, and (**f**) PANC-1. Housekeeping gene TBP was consistently stable across all radiation doses in A549, NCI-H226, Mia Paca-2 and PANC-1 while in SCC6 and SCC-1483, TFRC and IPO8 was stable respectively. By using the geNorm algorithm, the M values (average expression stability measure) were calculated to determine the stability of each HKG. The algorithm performs stepwise exclusion of the least stable HKG, and rank them from high to low M values, with the lowest M value being the most stable HKG. For each cell, geNorm was performed on data from each radiation dose, and the top five stable HKGs are presented in this figure (see Supplementary Figs S3–S5 for the complete geNorm results).

In SCC6 cells (Fig. 2a), we identified TFRC specific for 2, 4 and 6 Gy IR treatment (Fig. 2a, Supplementary Tables S15, S16, S17 and Fig. S3). TBP and UBC were specific for 2 and 6 Gy exclusively. In addition, ranking of the top five HKGs revealed YWHAZ and HMBS specific for 2 Gy while GUSB and B2M were specific for 6 Gy. Exclusive to 4 Gy were B2M, HPRT1, IPO8 and GAPDH. Interestingly, G6PD and ACTB which were found to be stable in non-irradiated controls (Supplementary Fig. S2a) were eliminated in the IR treatment groups suggesting that their individual expression was affected by IR doses. Therefore, identification of specific HKGs for a given IR dose is an important criterion for normalization in SCC6 cells.

Interestingly, while ranking the top five HKGs in SCC-1483 (Fig. 2b, Supplementary Tables S15, S16, S17 and Fig. S3), IPO8, the only stable gene amplified across all IR dose treatment groups, was absent in non-irradiated control (Supplementary Fig. S2b). This suggests that the expression of this gene is IR specific and IR may play a specific functional role in stabilizing its expression. In contrast to SCC6, we identified a set of HKGs – HMBS, IPO8 and TBP, stably expressed across all IR treatment groups and non-irradiated control. However, similar to SCC6 cells, single HKGs were specific for IR dose treatment groups. GAPDH was specific for 2 and 6 Gy doses while PGK1 and UBC were specific for 4 and 6 Gy dose treatment groups respectively.

### Stability of HKGs in non-small lung cancer

Next, we performed similar ranking in non-small lung cancer cell lines A549 (Fig. 2c) and NCI-H226 (Fig. 2d), with its corresponding non-irradiated controls (Supplementary Fig. S2c,d). Unlike head and neck cancer cell lines, TBP was stably expressed in A549 and NCI-H226 across all IR dose treatment groups while PP1A, GAPDH and B2M expression was exclusively expressed at 2, 4 and 6 Gy respectively (Fig. 2c,d Supplementary Tables S15, S16, S17 and Fig. S4). Likewise, in NCI-H226 cells (Fig. 2d), 4 out of 5 HKGs – TFRC, UBC, IPO8 and TBP was specific for 6 Gy IR treatment group only. Only IPO8 overlapped with 4 Gy treatment group while B2M was specific for 2 Gy treatment group. In summary, this analysis highlighted that the expression of TBP gene was resistant to fluctuations across all IR treatment groups in both cell lines and it could be used for normalizing gene expression data in these cell lines

### Stability of HKGs in pancreas cancer

Lastly, we compared the expression data of HKGs in pancreatic cancer cell lines MIA PaCa-2 (Fig. 2e), and PANC-1(Fig. 2f), to non-irradiated controls (Supplementary Fig. S2e,f). We estimated two stable universal HKGs – IPO8 and UBC to be expressed across all IR treatment groups compared to non-irradiated controls (Supplementary Fig. S2e,f) for Mia Paca-2 and PANC-1 respectively. Furthermore, expression levels of GUSB and TBP was specific for 2 and 4 Gy while the latter was also confirmed for 6 Gy treatment. In addition, HMBS gene, specific for 6 Gy did not overlap with other treatment groups (Fig. 2e,f, Supplementary Tables S15, S16, S17 and Fig. S5).

While the gene stability measure M for a control gene takes into account the arithmetic mean of all pairwise variation of all genes within the IR treatment group, it does not take into consideration the inter and intra-experimental variations that could arise due to IR or post-IR cellular responses. Therefore, we applied another statistical method known as the Normfinder. This method estimates the standard deviation (SD) of each HKG expression and in particular relevant for this study, it calculates the variation within an intra- (within an IR treatment) and inter- (between IR treatments) groups and assigns an SD value. This method rules out potential artifacts that can arise due to co-regulated genes.

**Figure 3:**
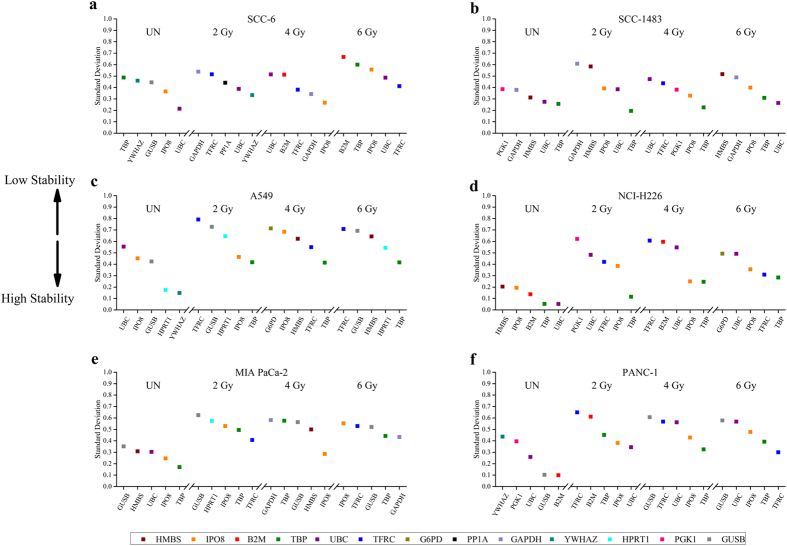
Top five stable genes identified by inter and intragroup variations by Normfinder for all radiated cancer cells with untreated control. (**a**) UBC for SCC6; (**b**) TBP and UBC for SCC-1483; (**c**) TBP for A549; (**d**) TBP, UBC and IPO8 for NCI-H226; (**e**) IPO8 and GUSB for Mia PaCa-2 and (**f**) UBC for PANC-1 were stable across all radiation doses The Normfinder algorithm estimates the SDs of each HKG in order to rank the genes based on their stabilities in the experiment. The lower the SD, the more stable the HKG. Like geNorm, the Normfinder was performed on data from each radiation level, and the top five stable HKGs are presented in this figure (see Supplementary Table S5 and Figs S6–S8 for the complete Normfinder results).

We applied this analysis to our dataset to rank the top five genes across all IR doses and time. The ranking of the top five genes estimated by Normfinder concurred with geNorm analyses for all tissue types with a marginal statistical exception in head and neck cancer cells. geNorm estimated TBP with an M value of 0.776 while UBC was 0.808 (ranked 6^th^) which was outside the top five genes specific for 2 Gy IR dose. However, Normfinder estimated both TBP and UBC to be in the top five ranked stable genes (Fig. 3a,b). This statistical variation for UBC and TBP is not significant but caution should be taken into consideration when measuring important target genes. The Normfinder algorithm revealed the following universal stable HKGs (Supplementary Tables S15, S16, S17) across all IR treatment groups and time when compared to non-irradiated cell lines: TBP and UBC for SCC6 and SCC-1483, A549, NCI-H226, IPO8, Mia PaCa-2 for PANC-1 (Fig. 3a–f, Table S17, Supplementary Figs S6, S7 and S8). It was interesting to note that geNorm did not rank a stable HKG for SCC6 but Normfinder estimated UBC gene to be stably expressed, across all IR treatment groups. Similarly, in A549, Normfinder did not validate any stable HKGs in the top five rank while geNorm estimated TBP gene to be stable while none of the top five ranked HKGs was validated by Normfinder in A549 cells. Taken together, majority of the genes identified by Normfinder analysis overlapped with geNorm algorithm for most IR treatment groups.

### Paired and single cell line analyses of stable HKGs in head and neck cancer, non-small lung and pancreas

To validate a stable HKG that shows little variation across all radiation doses and time points in a given cell line, we performed geNorm and Normfinder on the data. This was done by pairing the cell lines within in the same tissue type as well as individual cell line by grouping all radiation doses including 0 Gy and five time points. Data analysis and ranking of the top 5 HKGs using geNorm and Normfinder (Supplementary Table S15) identified **UBC, TBP, IPO8,** TFRC, GAPDH in head and neck cancer; **TBP, IPO8,** GUSB, UBC in lung and **TBP, IPO8, TFRC, GUSB,** HMBS in pancreas to be stable. In addition to the paired cell line analyses, we also performed geNorm and Normfinder on individual cell lines to identify common stable HKGs across all radiation doses including 0 Gy and five time points (Supplementary Table S16). In head and neck cancer, cell lines SCC6 and SCC-1483 identified **UBC, IPO8, TBP,** TFRC, and **UBC, IPO8, TBP,** HMBS to be stable respectively. In non-small lung cancer, cell lines A549 and NCI-H226 - **TBP, IPO8,** HPRT, TFRC and **TBP, IPO8,** TFRC, UBC while in pancreatic cancer, MIA PaCa-2 and PANC-1- **TBP, IPO8, TFRC, GUSB,** HMBS and **TBP, IPO8, TFRC, GUSB,** UBC were found to be stable respectively. Ranking of the stable HKGs from both these approaches identified that a subset of the genes, stable in the paired analyses, to be stable in individual cell line from the same tissue type, suggesting potential cellular differences within the same tissue type.

Having identified stable HKGs across all IR treatment groups by applying both these algorithms, we next sought to test the effects on a relevant target gene, EGFR. EGFR is over-expressed and/or frequently mutated in many human cancers. It is a clinically relevant target for inhibition by drug and radiation^16^. Clinical trials with EGFR inhibitors have failed to demonstrate efficacy, albeit preclinical studies have indicated a clear role of EGFR in pancreatic adenocarcinoma (PDAC)^17^,^18^,^19^, and its functional role in non-small lung^20^,^21^ and head and neck cancer^22^,^23^,^24^. We chose to validate the effect of these HKGs on EGFR expression in the head and neck cancer cell line SCC6.

### Validation of housekeeping genes with target gene EGFR in head and neck cancer

**Figure 4:**
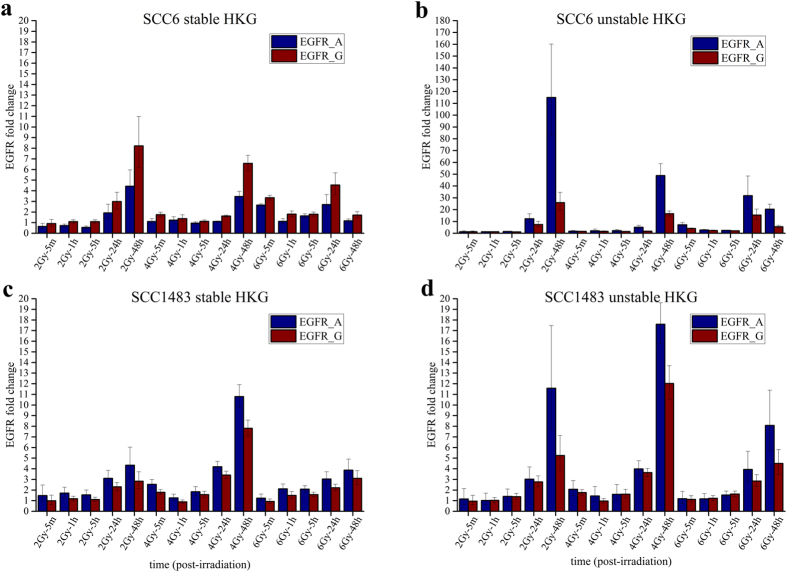
Time course expression of normalized EGFR expression across three independent radiation doses normalized to stable and unstable HKGs. (**a**) The expression levels of EGFR in SCC6 were normalized to geometric mean of TFRC, UBC, and IPO8, which are the top three stable HKGs for SCC6. EGFR was also normalized to arithmetic mean of UBC (most stable). (**b**) Normalization of EGFR in SCC6 using unstable HKGs: ACTB (most unstable), G6PD, and PGK1. (**c**) Normalization of EGFR in SCC-1483 using stable HKGs: TBP (most stable), IPO8, and UBC. (**d**) Normalization of EGFR in SCC-1483 using unstable HKGs: ACTB (most unstable), YWHAZ, and G6PD. Data was derived from three independent biological experiments with nine technical replicates.

We validated the fold change differences in expression of EGFR by applying geometric and arithmetic mean using the top and bottom three stable HKGs across all IR treatment groups (Fig. 4a–d). We calculated the ΔΔ Cq for EGFR expression. The fold change differences observed by applying the arithmetic mean varied from 1.2 to 16 fold for 2 Gy, 2.5 to 9.5 fold for 4 Gy and 5 to 7.5 fold for 6 Gy respectively. For geometric mean calculations, we added two more top ranked genes: TFRC and IPO8. As expected, applying geometric mean yielded smaller expression variation of 0.9 to 7 fold for 2 Gy, 1.5 to 6.5 fold for 4 Gy and 1.5 to 2.5 for Gy. Likewise, when we performed the same analysis using the bottom ranked genes ACTB, G6PD and PGK1, the variation in expression between arithmetic and geometric mean was very large. The highest fold change observed was 115 and 22 when applying the arithmetic and geometric mean respectively for 2 Gy. A similar trend was observed for 4 and 6 Gy IR treatment groups (Fig. 4b,d).

The results obtained here highlight the value of selecting optimal HKGs for normalization since indiscriminant application of common HKGs described in the literature may yield an overestimation of EGFR expression.

## Discussion

Careful selection and validation of HKGs is an important criterion for normalization of gene expression data for experimental conditions^25^. This is especially important when dealing with heterogeneous populations such as cancer cells derived from patient samples. Such efforts will prove valuable for preclinical studies that examine radiation alone, or radiation combined with agents that modulate molecular targets to alter radiation response^26^,^27^. To add to this variability, IR can have a significant impact on cellular processes that may influence the outcome of RNA expression. To minimize experimental fluctuations, we selected fourteen HKGs (Supplementary Table S1) that have different functional roles in the cell so as to reduce the probability of genes that might be co-regulated. In addition, primers designed for all these genes had a uniform annealing temperature of 61 °C and amplicon size of less than 150 bp which would also reduce the variability introduced due to PCR amplification efficiency. The HKGs selected were amplified in three different cancer cell types – head and neck, non-small lung and pancreas and irradiated 2, 4 and 6 Gy doses. The Cq data obtained from these HKGs were compared statistically using two independent algorithms which quantified the stability of HKGs in post-irradiated samples at different time intervals.

Systematic evaluation of the raw Cq values (Fig. 1, Supplementary Tables S2, S3, S4, S5, S6 and S7) revealed considerable variation in expression of several HKGs across cell lines and IR treatment doses while the HKGs within the cell line and across IR treatments had a much tighter distribution. However, the calculated SD and Cq values for a given HKG cannot be used for normalization because of the variability associated with total RNA extracted and the yield of reverse transcription reactions from samples isolated from different IR treatment groups. Furthermore, upon closer inspection of the interquartile range (Fig. 1), it was clear that that even within the same cancer type there was lack of consensus. While in both non-small lung cancer cell lines - A549 and NCI-H226, GUSB was the least variable gene; for the head and neck cancer cell lines - SCC6 and SC1483, HPRT1 and PP1A, respectively showed the smallest variation. Similarly, in pancreatic cell lines – MIA PaCa-2 and PANC-1, UBC and B2M were found to have the smallest variation. Hence, it was evident that even within the cancer subtype, Cq values of the same HKG were not absolutely reliable for normalization of gene expression. Another interesting finding among all cell lines was that traditional HKGs – GAPDH, G6PD and ACTB had higher interquartile range suggesting that IR treatments might affect their expression. This is not surprising especially for GAPDH, as it was reported to fluctuate in response to hypoxia, mitogens, EGF^1^ and also in tumor samples and cancer cells^28^,^29^,^30^. Taken together the raw Cq values are not a reliable factor for normalization, hence, we resorted to statistical algorithms such as geNorm and Normfinder, which reliably calculates a normalization factor for the most stable HKGs for a given experimental condition.

Although both the methods were able to rank the HKGs for their stability within and across IR treatment groups in all cell lines, we did not find many HKGs that overlapped with non-irradiated controls. When compared to the non-irradiated control SCC6 and SCC-1483 cells, only Normfinder estimated UBC and TBP to be stable across all IR treatments while in the pancreas cell lines TBP and IPO8 was calculated to be stable by geNorm and Normfinder. Likewise, we observed the opposite trend where geNorm estimated TBP to be stable in A549 cells across all IR treatment groups when compared to non-irradiated control while TBP and IPO8 was stable using the Normfinder algorithm. We inferred from these results that selection of HKGs is IR dose specific since the stability of YWHAZ and UBC from 2 Gy and IPO8 from 4 Gy overlapped with non-irradiated control in SCC6 cells while PP1A, HPRT1, GUSB and IPO8; GAPDH and IPO8 and B2M; GUSB and HPRT1 genes were specific for the 2, 4 and 6 Gy treatment groups respectively. For all other cell lines, the algorithms were able to estimate overlapping genes across all IR treatment groups when compared to non-irradiated controls.

In summary, our data suggests that stability of HKGs can be influenced by the dose of ionizing radiation. We propose the following guidelines for implementing normalization of gene expression involving ionizing radiation across multiple time points and various radiation doses. (i) Careful selection of stable HKGs that show little experimental and Cq variation across multiple time points and various doses including 0 Gy. (ii) Identifying stable HKGs by grouping together all doses including 0 Gy and time points within a cancer cell line or multiple cell lines from the same cancer tissue type and (iii) applying geometric mean to calculate fold changes over arithmetic mean. We have listed the top five ranked stable HKGs identified in commonly used cancer cell lines across IR treatment groups which could be utilized as a resource for normalization of gene expression data. In addition, selection of stable HKGs with independent cellular functions that are unlikely to be co-regulated in radiation studies would be critical for normalization. Furthermore, our data suggest that use of more than one HKG yields a better estimate of normalizing gene expression across IR treatment groups.

## Methods

### Cell lines

Six different human tumor cell lines, i.e., the non-small non-small lung adenocarcinoma A549 and NCI-H226, head and neck squamous cell carcinoma – UMSCC-6 provided by Dr. Thomas E. Carey (Univeristy of Michigan, Ann Arbor, MI), SCC-1483 provided by Dr. Jennifer Grandis (University of California, San Francisco, CA), and pancreatic adenocarcinoma – PANC-1 and MIA PaCa-2 provided by Dr. George Wilson, Beaumont health system, MI were used for the study. Cell lines were cultured in Dulbecco’s Modified Eagle’s Medium (DMEM) or RPMI-1640 (Life Technologies) under standard conditions in a fully humidified incubator with 5.0% CO_2_ at 37.0 °C.

### Irradiation

Cells were irradiated in 10 cm dish with 2, 4 and 6 Gy of photon with a Shepherd & Associates Model 109 irradiator (San Fernando, CA) and a^137^ cesium hotbox source. After irradiation, the cells were incubated for 5 minutes, 1, 5, 24 and 48 h at 37.0 °C and 5% CO_2_. Control cells were treated identically but without irradiation. Cells were scrapped using the cell scraper after adding 1 ml TRIzol (Invitrogen) and collected in 1.5 ml Eppendorf tubes, flash frozen in liquid nitrogen and subsequently stored at −80.0 °C.

### RNA isolation and cDNA synthesis

RNA was isolated in phase lock tubes using TRIzol (Invitrogen) according to the manufacturer’s protocol. To avoid genomic DNA contamination RNA was treated with Dnase I (Qiagen). Purified RNA was eluted in 20.0 μL of nuclease-free water and stored at −20.0 °C. RNA concentration and purity was assessed using a Nanodrop ND-1000 spectrophotometer (Peqlab). RNA (1.0 μg) was subjected to reverse transcription reaction using the high-capacity quantitect reverse transcription kit (Qiagen) according to the manufacturer’s protocol. Relative mRNA levels were quantified via real-time PCR (RT-qPCR) using a Bio-Rad C1000 qPCR Detection System and Power SYBR Green PCR Master Mix as recommended by the manufacturer (Life Technologies). All reactions were performed in triplicate from RNA isolated from three independent biological experiments.

### Automation

All qPCR reactions were set up on an automated robot platform (Gilson, Inc Middleton, WI, USA) and PIPETMAX qPCR assistant. The Gilson lab wizard was programmed to set up all qPCR reactions.

### Data and Statistical Analysis

After obtaining raw Cq data from qPCR, all calculations of mean Cq, standard deviation, and coefficient of variance were carried out using Matlab (The MathWork Inc., Natick, MA) and Origin 2015 (OriginLab, Northampton, MA). The stability values of gene expressions were computed using geNorm^15^ and NormFinder^31^ algorithms respectively. The HKGs were ranked according to their stability values generated by geNorm (Figs S2–S5 and Tables S15–S16) and NormFinder (Tables S15–S17). Based on the overall stability across all conditions, the top three most stable HKGs and the bottom three most unstable HKGs within SCC6 and SCC-1483 were selected to demonstrate and compare the effect of using stable or unstable HKGs as normalization factors. In the order of their stability (or instability), TFRC, UBC, IPO8 were selected as the top three most stable HKGs, and ACTB, G6PD, PGK1 were selected as the most unstable HKGs for SCC6. TBP, UBC, IPO8 were the most stable HKGs, and ACTB, G6PD, YWHAZ were the most unstable HKGs for SCC-1483. Following the ΔΔCq method, EGFR was selected as the target gene, while either the geometric mean of the three most stable HKGs or the three most unstable HKGs was used as the reference for normalization. Arithmetic mean of either the most stable HKG or most unstable HKG was also calculated to serve as comparison to results of using the geometric mean.

## Additional Information

**How to cite this article**: Iyer, G. *et al*. Identification of stable housekeeping genes in response to ionizing radiation in cancer research. *Sci. Rep.* **7**, 43763; doi: 10.1038/srep43763 (2017).

**Publisher's note:** Springer Nature remains neutral with regard to jurisdictional claims in published maps and institutional affiliations.

## Supplementary Material

Supplementary Information

Supplementary Dataset 1
