# Supplementary material for: Identification of stable housekeeping genes in response to ionizing radiation in cancer research
Source: Sci Rep. 2017 Mar 6;7:43763. doi: 10.1038/srep43763 (PMC5338320; doi:10.1038/srep43763)
Supplement: Supplementary Information [file srep43763-s1.pdf]

Identification of stable housekeeping genes in response to ionizing radiation in cancer research

Gopal Iyer*, Albert Wang, Sean Brennan, Shay Bourgeois, Eric Armstrong, Pari Shah and Paul. M Harari*

Department of Human Oncology and the University of Wisconsin School of Medicine and Public Health, University of Wisconsin-Madison, Madison

*** - Giyer@humonc.wisc.edu and harari@humonc.wisc.edu**

Supplementary

Table S1. Primer sequences of housekeeping genes used in this study.

Gene function	Gene	RefSeq	Exon Location	Primer For/Rev	Amplicon (bp)	Start Position	End Position
glucose-6-phosphate dehydrogenase	G6PD	NM_001042351	1 to 2	GTATCCGACTGATGGAAGGC/TCCGGAGAGAAGTCTGAGTC	150	56	205
B-Actin	ACTB	NM_001101	1 to 2	CCTTGCACATGCCGGAG/ACAGAGCCTCGCCTTTG	110	30	139
Glyceraldehyde-3-Phosphate Dehydrogenase	GAPDH	NM_002046	2 to 3	TGTAGTTGAGGTCAATGAAGGG/ACATCGCTCAGACACCATG	143	87	229
hypoxanthine phosphoribosyltransferase 1	HPRT1	NM_000194	8 to 9	GCGATGTCAATAGGACTCCAG/TTGTTGTAGGATATGCCCTTGA	149	727	875
Beta-2-Microglobulin	B2M	NM_004048	2 to 4	ACCTCCATGATGCTGCTTAC/GGACTGGTCTTTCTATCTCTTGT	143	294	436
Beta Glucuronidase	GUSB	NM_000181	10 to 11	GTTTTTGATCCAGACCCAGATG/GCCCATTATTCAGAGCGAGTA	128	1733	1860
Importin 8	IPO8	NM_006390	1 to 2	GAATTCCACATGGTCAGAGACT/CGAAGTTGCGGATTGCAG	104	395	498
Ubiquitin C	UBC	NM_021009	1 to 2	CCTTATCTTGGATCTTTGCCTTG/GATTTGGGTCGCAGTTCTTG	150	399	548
Peptidylprolyl Isomerase A (Cyclophilin A	PPIA	NM_021130	4 to 5	TCTTTCACTTTGCCAAACACC/CATCCTAAAGCATACGGGTCC	138	347	484
TATA Box Binding Protein	TBP	NM_003194	1 to 2	CAGCAACTTCCTCAATTCCTTG/GCTGTTTAACTTCGCTTCCG	104	81	184
Hydroxymethylbilane Synthase	HMBS	NM_001258209	1 to 2	CACTCTCATCTTTGGGCTGTT/CACACTTTGGGCTTCTGGA	95	214	308
Tyrosine 3-Monooxygenase	YWHAZ	NM_001135702	1 to 2	TGCTCAGTTACAGACTTCATGC/GTAACCGACGTCTCAAGTCAG	127	146	272
Phosphoglycerate Kinase 1	PGK1	NM_000291	1 to 3	GACAGCAGCCTTAATCCTCTG/CTAACAAGCTGACGCTGGA	122	183	304
Transferrin Receptor	TFRC	NM_001128148	1 to 3	CCACCAAACAAGTTAGAGAATGC/TCAGAGCGTCGGGATATCG	114	70	183

Table S2. Mean Cq values, standard deviation (SD), standard error of mean, 95% confidence intervals, variance and coefficient of variation (CV) of 14 housekeeping genes (HKGs) across SCC-6 cells (head and neck cancer cell line) with various levels of radiation treatment (2 Gy, 4 Gy, and 6 Gy).

SCC-6		Mean	Standard Deviation	SE of mean	Lower 95% CI of Mean	Upper 95% CI of Mean	Variance	Coefficient of Variation
**G6PD**	2 Gy	27.007	1.676	0.250	26.503	27.510	2.811	0.062
	4 Gy	26.512	1.133	0.169	26.172	26.852	1.284	0.043
	6 Gy	26.510	1.504	0.224	26.058	26.962	2.262	0.057
**IPO8**	2 Gy	25.549	1.226	0.183	25.180	25.917	1.504	0.048
	4 Gy	25.218	0.900	0.134	24.948	25.488	0.810	0.036
	6 Gy	25.052	1.154	0.172	24.705	25.399	1.332	0.046
**PGK1**	2 Gy	22.743	1.903	0.284	22.172	23.315	3.620	0.084
	4 Gy	21.671	1.188	0.177	21.314	22.028	1.411	0.055
	6 Gy	22.476	1.795	0.268	21.937	23.016	3.223	0.080
**PP1A**	2 Gy	17.733	0.875	0.130	17.470	17.996	0.766	0.049
	4 Gy	17.249	1.170	0.174	16.898	17.601	1.369	0.068
	6 Gy	17.738	1.112	0.166	17.404	18.072	1.236	0.063
**HMBS**	2 Gy	31.621	1.157	0.173	31.273	31.969	1.340	0.037
	4 Gy	30.968	0.722	0.108	30.751	31.184	0.521	0.023
	6 Gy	31.570	1.291	0.192	31.182	31.958	1.666	0.041
**GUSB**	2 Gy	23.740	0.666	0.099	23.540	23.941	0.444	0.028
	4 Gy	23.419	0.620	0.092	23.233	23.606	0.384	0.026
	6 Gy	23.674	0.935	0.139	23.393	23.955	0.874	0.039
**UBC**	2 Gy	21.306	1.061	0.158	20.987	21.625	1.126	0.050
	4 Gy	21.177	0.583	0.087	21.002	21.352	0.339	0.028
	6 Gy	21.180	0.880	0.131	20.915	21.444	0.775	0.042
**YWHAZ**	2 Gy	25.461	0.985	0.147	25.165	25.757	0.969	0.039
	4 Gy	25.382	0.592	0.088	25.204	25.560	0.351	0.023
	6 Gy	25.269	0.754	0.112	25.043	25.496	0.569	0.030
**GAPDH**	2 Gy	18.968	1.267	0.189	18.587	19.348	1.605	0.067
	4 Gy	17.972	0.887	0.132	17.705	18.238	0.787	0.049
	6 Gy	18.842	1.560	0.233	18.373	19.310	2.434	0.083
**HPRT1**	2 Gy	21.683	0.875	0.130	21.420	21.946	0.765	0.040
	4 Gy	21.356	1.089	0.162	21.029	21.684	1.187	0.051
	6 Gy	21.736	1.164	0.174	21.386	22.085	1.356	0.054
**ACTB**	2 Gy	23.244	2.292	0.342	22.556	23.933	5.253	0.099
	4 Gy	22.709	1.972	0.294	22.116	23.301	3.890	0.087
	6 Gy	23.194	1.945	0.290	22.610	23.778	3.783	0.084
**B2M**	2 Gy	18.703	0.860	0.128	18.445	18.962	0.739	0.046
	4 Gy	18.740	0.810	0.121	18.497	18.984	0.657	0.043
	6 Gy	19.226	0.749	0.112	19.001	19.451	0.561	0.039
**TBP**	2 Gy	25.611	0.962	0.143	25.322	25.900	0.925	0.038
	4 Gy	25.291	0.659	0.098	25.093	25.489	0.434	0.026
	6 Gy	25.358	1.175	0.175	25.005	25.711	1.381	0.046
**TFRC**	2 Gy	23.499	1.088	0.162	23.172	23.826	1.183	0.046
	4 Gy	22.893	0.899	0.134	22.623	23.163	0.808	0.039
	6 Gy	23.000	1.193	0.178	22.641	23.358	1.422	0.052

Table S3. Mean Cq values, standard deviation (SD), standard error of mean, 95% confidence intervals, variance and coefficient of variation (CV) of 14 housekeeping genes (HKGs) across SCC-1483 cells (head and neck cancer cell line) with various levels of radiation treatment (2 Gy, 4 Gy, and 6 Gy).

SCC-1483		Mean	Standard Deviation	SE of mean	Lower 95% CI of Mean	Upper 95% CI of Mean	Variance	Coefficient of Variation
**G6PD**	2 Gy	24.048	1.800	0.268	23.507	24.588	3.238	0.075
	4 Gy	23.209	1.224	0.182	22.842	23.577	1.497	0.053
	6 Gy	23.632	1.314	0.196	23.237	24.027	1.727	0.056
**IPO8**	2 Gy	24.153	1.367	0.204	23.742	24.564	1.869	0.057
	4 Gy	23.635	1.010	0.151	23.331	23.939	1.021	0.043
	6 Gy	24.034	1.082	0.161	23.709	24.359	1.171	0.045
**PGK1**	2 Gy	20.539	1.582	0.236	20.064	21.015	2.504	0.077
	4 Gy	20.275	1.087	0.162	19.949	20.602	1.182	0.054
	6 Gy	20.626	1.175	0.175	20.273	20.979	1.382	0.057
**PP1A**	2 Gy	16.897	0.987	0.147	16.600	17.193	0.975	0.058
	4 Gy	16.991	0.907	0.135	16.718	17.263	0.822	0.053
	6 Gy	16.999	0.838	0.125	16.747	17.251	0.703	0.049
**HMBS**	2 Gy	29.544	1.531	0.228	29.084	30.003	2.343	0.052
	4 Gy	28.991	1.062	0.158	28.672	29.310	1.128	0.037
	6 Gy	29.396	1.076	0.160	29.073	29.720	1.157	0.037
**GUSB**	2 Gy	22.638	0.783	0.117	22.403	22.873	0.612	0.035
	4 Gy	22.547	0.530	0.079	22.388	22.707	0.281	0.024
	6 Gy	22.570	0.496	0.074	22.421	22.719	0.246	0.022
**UBC**	2 Gy	20.541	1.082	0.161	20.216	20.866	1.170	0.053
	4 Gy	20.289	0.915	0.136	20.014	20.564	0.838	0.045
	6 Gy	20.350	0.932	0.139	20.070	20.630	0.868	0.046
**YWHAZ**	2 Gy	24.912	0.841	0.125	24.659	25.164	0.707	0.034
	4 Gy	24.759	0.708	0.106	24.546	24.972	0.501	0.029
	6 Gy	24.866	0.844	0.126	24.612	25.119	0.712	0.034
**GAPDH**	2 Gy	17.068	1.575	0.235	16.594	17.541	2.481	0.092
	4 Gy	16.753	1.387	0.207	16.336	17.169	1.924	0.083
	6 Gy	17.071	1.167	0.174	16.720	17.421	1.362	0.068
**HPRT1**	2 Gy	21.323	0.990	0.148	21.025	21.620	0.979	0.046
	4 Gy	21.037	0.827	0.123	20.789	21.286	0.683	0.039
	6 Gy	21.437	0.696	0.104	21.228	21.647	0.485	0.032
**ACTB**	2 Gy	20.717	2.076	0.309	20.093	21.341	4.310	0.100
	4 Gy	20.194	1.510	0.225	19.740	20.647	2.279	0.075
	6 Gy	20.383	1.589	0.237	19.906	20.860	2.524	0.078
**B2M**	2 Gy	19.156	0.773	0.115	18.923	19.388	0.598	0.040
	4 Gy	18.971	0.613	0.091	18.787	19.155	0.376	0.032
	6 Gy	19.186	0.575	0.086	19.014	19.359	0.330	0.030
**TBP**	2 Gy	24.697	1.188	0.177	24.340	25.054	1.411	0.048
	4 Gy	24.136	0.725	0.108	23.918	24.354	0.526	0.030
	6 Gy	24.376	0.703	0.105	24.164	24.587	0.494	0.029
**TFRC**	2 Gy	21.354	1.495	0.223	20.905	21.803	2.236	0.070
	4 Gy	20.621	0.931	0.139	20.341	20.900	0.867	0.045
	6 Gy	21.116	1.107	0.165	20.784	21.449	1.225	0.052

Table S4. Mean Cq values, standard deviation (SD), standard error of mean, 95% confidence intervals, variance and coefficient of variation (CV) of 14 HKGs across A549 cells (lung cancer cell line) with various levels of radiation treatment (2 Gy, 4 Gy, and 6 Gy).

A549		Mean	Standard Deviation	SE of mean	Lower 95% CI of Mean	Upper 95% CI of Mean	Variance	Coefficient of Variation
**G6PD**	2 Gy	22.237	1.265	0.189	21.857	22.617	1.600	0.057
	4 Gy	22.466	1.119	0.167	22.130	22.803	1.251	0.050
	6 Gy	22.258	1.218	0.182	21.892	22.625	1.485	0.055
**IPO8**	2 Gy	24.697	0.766	0.114	24.467	24.928	0.587	0.031
	4 Gy	25.415	0.980	0.146	25.121	25.710	0.960	0.039
	6 Gy	25.149	0.884	0.132	24.883	25.415	0.781	0.035
**PGK1**	2 Gy	20.803	0.956	0.143	20.516	21.091	0.914	0.046
	4 Gy	21.208	1.012	0.151	20.904	21.512	1.024	0.048
	6 Gy	20.928	0.988	0.147	20.631	21.225	0.977	0.047
**PP1A**	2 Gy	18.161	0.938	0.140	17.879	18.443	0.881	0.052
	4 Gy	18.781	1.110	0.166	18.447	19.114	1.233	0.059
	6 Gy	18.618	0.958	0.143	18.330	18.906	0.918	0.051
**HMBS**	2 Gy	29.909	1.002	0.149	29.608	30.210	1.004	0.034
	4 Gy	29.814	0.940	0.140	29.532	30.096	0.883	0.032
	6 Gy	29.911	0.861	0.128	29.653	30.170	0.742	0.029
**GUSB**	2 Gy	23.675	0.641	0.095	23.482	23.867	0.410	0.027
	4 Gy	23.520	0.712	0.106	23.306	23.734	0.507	0.030
	6 Gy	23.895	0.465	0.069	23.756	24.035	0.216	0.019
**UBC**	2 Gy	22.175	1.007	0.150	21.872	22.477	1.015	0.045
	4 Gy	21.692	1.114	0.166	21.357	22.027	1.242	0.051
	6 Gy	22.040	1.016	0.151	21.734	22.345	1.033	0.046
**YWHAZ**	2 Gy	26.254	1.151	0.172	25.908	26.599	1.325	0.044
	4 Gy	25.875	1.531	0.228	25.415	26.335	2.343	0.059
	6 Gy	26.243	1.099	0.164	25.913	26.573	1.209	0.042
**GAPDH**	2 Gy	17.251	1.148	0.171	16.906	17.596	1.317	0.067
	4 Gy	17.388	1.105	0.165	17.056	17.720	1.221	0.064
	6 Gy	17.053	1.108	0.165	16.720	17.386	1.229	0.065
**HPRT1**	2 Gy	21.165	0.831	0.124	20.916	21.415	0.691	0.039
	4 Gy	21.469	0.611	0.091	21.285	21.652	0.373	0.028
	6 Gy	21.661	0.577	0.086	21.488	21.834	0.333	0.027
**ACTB**	2 Gy	21.080	2.475	0.369	20.336	21.824	6.126	0.117
	4 Gy	22.981	1.565	0.233	22.511	23.451	2.451	0.068
	6 Gy	22.471	1.572	0.234	21.999	22.943	2.471	0.070
**B2M**	2 Gy	19.159	0.915	0.136	18.884	19.434	0.837	0.048
	4 Gy	19.380	0.937	0.140	19.099	19.662	0.878	0.048
	6 Gy	19.448	0.705	0.105	19.236	19.660	0.497	0.036
**TBP**	2 Gy	26.105	0.737	0.110	25.884	26.327	0.543	0.028
	4 Gy	26.214	0.879	0.131	25.950	26.478	0.772	0.034
	6 Gy	26.126	0.649	0.097	25.931	26.321	0.421	0.025
**TFRC**	2 Gy	23.687	0.925	0.138	23.409	23.965	0.855	0.039
	4 Gy	23.938	0.903	0.135	23.667	24.209	0.815	0.038
	6 Gy	23.837	0.880	0.131	23.573	24.102	0.775	0.037

Table S5. Mean Cq values, standard deviation (SD), standard error of mean, 95% confidence intervals, variance and coefficient of variation (CV) of 14 HKGs across NCI-H226 cells (lung cancer cell line) with various levels of radiation treatment (2 Gy, 4 Gy, and 6 Gy).

NCI-H226		Mean	Standard Deviation	SE of mean	Lower 95% CI of Mean	Upper 95% CI of Mean	Variance	Coefficient of Variation
**G6PD**	2 Gy	26.261	1.870	0.341	25.563	26.959	3.496	0.071
	4 Gy	25.941	1.302	0.238	25.455	26.428	1.696	0.050
	6 Gy	25.263	1.311	0.239	24.774	25.753	1.718	0.052
**IPO8**	2 Gy	25.435	1.583	0.289	24.844	26.026	2.507	0.062
	4 Gy	25.372	1.116	0.204	24.955	25.789	1.246	0.044
	6 Gy	24.787	1.173	0.214	24.349	25.225	1.377	0.047
**PGK1**	2 Gy	22.055	1.901	0.347	21.345	22.765	3.614	0.086
	4 Gy	21.823	1.477	0.270	21.272	22.375	2.181	0.068
	6 Gy	21.415	1.822	0.333	20.735	22.095	3.320	0.085
**PP1A**	2 Gy	18.553	1.503	0.274	17.992	19.114	2.258	0.081
	4 Gy	18.343	1.491	0.272	17.786	18.899	2.223	0.081
	6 Gy	18.228	1.526	0.279	17.658	18.798	2.330	0.084
**HMBS**	2 Gy	30.435	1.449	0.265	29.894	30.976	2.100	0.048
	4 Gy	30.554	1.145	0.209	30.127	30.982	1.311	0.037
	6 Gy	30.072	1.410	0.258	29.546	30.599	1.989	0.047
**GUSB**	2 Gy	24.352	1.234	0.225	23.891	24.813	1.523	0.051
	4 Gy	24.228	0.870	0.159	23.904	24.553	0.756	0.036
	6 Gy	23.979	0.917	0.167	23.636	24.321	0.841	0.038
**UBC**	2 Gy	21.996	1.113	0.203	21.581	22.412	1.239	0.051
	4 Gy	21.883	0.705	0.129	21.619	22.146	0.497	0.032
	6 Gy	21.544	1.015	0.185	21.165	21.923	1.030	0.047
**YWHAZ**	2 Gy	26.426	1.429	0.261	25.892	26.960	2.043	0.054
	4 Gy	26.235	1.041	0.190	25.846	26.623	1.083	0.040
	6 Gy	25.834	1.334	0.244	25.335	26.332	1.780	0.052
**GAPDH**	2 Gy	18.066	2.121	0.387	17.274	18.858	4.500	0.117
	4 Gy	17.658	1.480	0.270	17.105	18.210	2.191	0.084
	6 Gy	16.979	1.544	0.282	16.403	17.556	2.384	0.091
**HPRT1**	2 Gy	23.248	1.316	0.240	22.757	23.740	1.732	0.057
	4 Gy	23.277	0.950	0.174	22.922	23.632	0.903	0.041
	6 Gy	23.185	1.149	0.210	22.756	23.615	1.320	0.050
**ACTB**	2 Gy	22.150	3.056	0.558	21.008	23.291	9.339	0.138
	4 Gy	22.221	2.197	0.401	21.400	23.041	4.825	0.099
	6 Gy	21.761	2.785	0.508	20.721	22.801	7.756	0.128
**B2M**	2 Gy	17.928	1.392	0.254	17.408	18.447	1.937	0.078
	4 Gy	17.490	0.909	0.166	17.151	17.830	0.826	0.052
	6 Gy	17.061	1.034	0.189	16.675	17.448	1.069	0.061
**TBP**	2 Gy	26.177	1.469	0.268	25.629	26.726	2.158	0.056
	4 Gy	25.922	0.926	0.169	25.576	26.268	0.858	0.036
	6 Gy	25.385	1.031	0.188	25.000	25.770	1.062	0.041
**TFRC**	2 Gy	22.601	1.361	0.249	22.093	23.110	1.853	0.060
	4 Gy	22.301	1.082	0.198	21.897	22.705	1.170	0.049
	6 Gy	21.592	1.025	0.187	21.209	21.975	1.052	0.047

Table S6. Mean Cq values, standard deviation (SD), standard error of mean, 95% confidence intervals, variance and coefficient of variation (CV) of 14 HKGs across MIA PaCa-2 cells (pancreas cancer cell lines) with various levels of radiation treatment (2 Gy, 4 Gy, and 6 Gy).

MIA PaCa-2		Mean	Standard Deviation	SE of mean	Lower 95% CI of Mean	Upper 95% CI of Mean	Variance	Coefficient of Variation
**G6PD**	2 Gy	25.674	1.744	0.260	25.150	26.198	3.041	0.068
	4 Gy	26.407	1.239	0.185	26.035	26.779	1.535	0.047
	6 Gy	25.513	1.458	0.217	25.075	25.951	2.125	0.057
**IPO8**	2 Gy	24.436	1.567	0.234	23.965	24.907	2.456	0.064
	4 Gy	25.027	1.037	0.155	24.716	25.339	1.075	0.041
	6 Gy	24.642	1.057	0.158	24.324	24.959	1.116	0.043
**PGK1**	2 Gy	21.565	2.016	0.301	20.960	22.171	4.065	0.093
	4 Gy	21.948	1.600	0.238	21.467	22.428	2.559	0.073
	6 Gy	21.280	1.726	0.257	20.761	21.799	2.980	0.081
**PP1A**	2 Gy	18.162	1.715	0.256	17.646	18.677	2.942	0.094
	4 Gy	19.328	2.123	0.317	18.690	19.966	4.508	0.110
	6 Gy	18.356	1.521	0.227	17.899	18.813	2.315	0.083
**HMBS**	2 Gy	30.749	1.186	0.177	30.393	31.106	1.406	0.039
	4 Gy	31.080	1.188	0.177	30.724	31.437	1.410	0.038
	6 Gy	30.352	0.980	0.146	30.057	30.646	0.960	0.032
**GUSB**	2 Gy	23.351	1.331	0.198	22.951	23.751	1.772	0.057
	4 Gy	23.679	1.373	0.205	23.266	24.091	1.884	0.058
	6 Gy	23.061	1.239	0.185	22.689	23.434	1.536	0.054
**UBC**	2 Gy	21.547	0.757	0.113	21.320	21.775	0.573	0.035
	4 Gy	21.550	0.695	0.104	21.341	21.759	0.483	0.032
	6 Gy	21.449	0.565	0.084	21.279	21.619	0.320	0.026
**YWHAZ**	2 Gy	27.167	0.804	0.120	26.926	27.409	0.646	0.030
	4 Gy	27.578	0.941	0.140	27.295	27.861	0.885	0.034
	6 Gy	26.839	0.874	0.130	26.577	27.102	0.764	0.033
**GAPDH**	2 Gy	16.962	1.889	0.282	16.395	17.530	3.567	0.111
	4 Gy	17.870	1.354	0.202	17.463	18.277	1.833	0.076
	6 Gy	16.443	1.238	0.185	16.071	16.815	1.533	0.075
**HPRT1**	2 Gy	20.912	1.644	0.245	20.418	21.406	2.702	0.079
	4 Gy	21.735	1.859	0.277	21.176	22.293	3.458	0.086
	6 Gy	20.852	1.300	0.194	20.462	21.243	1.689	0.062
**ACTB**	2 Gy	21.896	1.958	0.292	21.308	22.484	3.834	0.089
	4 Gy	22.384	1.564	0.233	21.914	22.854	2.447	0.070
	6 Gy	21.475	1.861	0.277	20.916	22.034	3.465	0.087
**B2M**	2 Gy	19.569	1.354	0.202	19.162	19.976	1.834	0.069
	4 Gy	20.033	1.415	0.211	19.608	20.458	2.002	0.071
	6 Gy	19.410	1.382	0.206	18.995	19.825	1.909	0.071
**TBP**	2 Gy	25.564	1.053	0.157	25.248	25.880	1.108	0.041
	4 Gy	25.977	0.805	0.120	25.735	26.219	0.649	0.031
	6 Gy	25.259	0.974	0.145	24.966	25.551	0.949	0.039
**TFRC**	2 Gy	22.831	1.210	0.180	22.467	23.194	1.464	0.053
	4 Gy	23.526	1.120	0.167	23.189	23.862	1.254	0.048
	6 Gy	22.510	1.297	0.193	22.121	22.900	1.681	0.058

Table S7. Mean Cq values, standard deviation (SD), standard error of mean, 95% confidence intervals, variance and coefficient of variation (CV) of 14 HKGs across PANC-1 cells (pancreas cancer cell lines) with various levels of radiation treatment (2 Gy, 4 Gy, and 6 Gy).

PANC-1		Mean	Standard Deviation	SE of mean	Lower 95% CI of Mean	Upper 95% CI of Mean	Variance	Coefficient of Variation
**G6PD**	2 Gy	23.976	2.893	0.431	23.106	24.845	8.368	0.121
	4 Gy	22.924	2.030	0.303	22.314	23.534	4.122	0.089
	6 Gy	22.304	1.660	0.247	21.806	22.803	2.755	0.074
**IPO8**	2 Gy	24.199	1.275	0.190	23.816	24.582	1.625	0.053
	4 Gy	24.681	1.529	0.228	24.222	25.140	2.337	0.062
	6 Gy	24.125	1.340	0.200	23.722	24.527	1.794	0.056
**PGK1**	2 Gy	20.623	1.741	0.260	20.100	21.146	3.032	0.084
	4 Gy	20.914	1.818	0.271	20.367	21.460	3.306	0.087
	6 Gy	20.113	1.679	0.250	19.609	20.618	2.819	0.083
**PP1A**	2 Gy	17.600	1.398	0.208	17.180	18.020	1.955	0.079
	4 Gy	17.542	1.099	0.164	17.212	17.872	1.208	0.063
	6 Gy	17.488	1.251	0.186	17.112	17.863	1.564	0.072
**HMBS**	2 Gy	29.639	1.220	0.182	29.273	30.006	1.488	0.041
	4 Gy	29.895	1.359	0.203	29.487	30.303	1.846	0.045
	6 Gy	29.217	0.803	0.120	28.976	29.459	0.644	0.027
**GUSB**	2 Gy	23.280	0.787	0.117	23.044	23.516	0.619	0.034
	4 Gy	23.389	0.974	0.145	23.096	23.681	0.949	0.042
	6 Gy	23.151	0.723	0.108	22.933	23.368	0.522	0.031
**UBC**	2 Gy	21.909	1.114	0.166	21.575	22.244	1.240	0.051
	4 Gy	22.157	1.573	0.234	21.685	22.630	2.473	0.071
	6 Gy	21.531	1.087	0.162	21.204	21.857	1.182	0.050
**YWHAZ**	2 Gy	25.756	1.156	0.172	25.408	26.103	1.337	0.045
	4 Gy	26.252	1.276	0.190	25.869	26.636	1.629	0.049
	6 Gy	25.965	0.841	0.125	25.713	26.218	0.708	0.032
**GAPDH**	2 Gy	18.003	1.878	0.280	17.439	18.568	3.528	0.104
	4 Gy	17.840	1.404	0.209	17.418	18.262	1.972	0.079
	6 Gy	17.742	1.633	0.243	17.251	18.232	2.667	0.092
**HPRT1**	2 Gy	20.849	0.971	0.145	20.557	21.140	0.942	0.047
	4 Gy	20.849	0.925	0.138	20.571	21.127	0.856	0.044
	6 Gy	20.810	0.785	0.117	20.574	21.046	0.617	0.038
**ACTB**	2 Gy	21.717	2.185	0.326	21.060	22.373	4.775	0.101
	4 Gy	22.255	2.168	0.323	21.604	22.906	4.698	0.097
	6 Gy	21.907	2.133	0.318	21.267	22.548	4.548	0.097
**B2M**	2 Gy	18.185	0.918	0.137	17.909	18.461	0.843	0.050
	4 Gy	18.145	0.775	0.116	17.912	18.378	0.601	0.043
	6 Gy	18.257	0.809	0.121	18.014	18.501	0.655	0.044
**TBP**	2 Gy	24.592	1.168	0.174	24.241	24.942	1.363	0.047
	4 Gy	24.964	1.398	0.208	24.544	25.384	1.953	0.056
	6 Gy	24.513	1.054	0.157	24.197	24.830	1.110	0.043
**TFRC**	2 Gy	21.790	1.438	0.214	21.358	22.222	2.067	0.066
	4 Gy	22.337	1.613	0.240	21.852	22.821	2.601	0.072
	6 Gy	21.816	1.216	0.181	21.451	22.182	1.479	0.056

Table S8. Mean Cq values, standard deviation (SD), standard error of mean, 95% confidence intervals, variance and coefficient of variation (CV) of 14 HKGs across SCC-6 cells (head and neck cancer cell line) with various levels of radiation treatment (2 Gy, 4 Gy, and 6 Gy) and time post-irradiation (5 min, 1, 5, 24, and 48 h).

SCC-6			Mean	Standard Deviation	Variance	SE of mean	Lower 95% CI of Mean	Upper 95% CI of Mean	Coefficient of Variation
**G6PD**	2 Gy	5min	26.834	2.408	5.797	0.803	24.984	28.685	0.090
		60min	26.523	1.638	2.682	0.546	25.264	27.782	0.062
		5hr	26.324	1.774	3.146	0.591	24.961	27.687	0.067
		24hr	27.286	0.329	0.108	0.110	27.033	27.539	0.012
		48hr	28.066	1.247	1.554	0.416	27.108	29.024	0.044
	4 Gy	5min	26.406	0.721	0.520	0.240	25.851	26.960	0.027
		60min	26.740	1.418	2.012	0.473	25.650	27.831	0.053
		5hr	27.031	1.198	1.434	0.399	26.111	27.952	0.044
		24hr	25.550	1.042	1.085	0.347	24.749	26.350	0.041
		48hr	26.833	0.689	0.474	0.230	26.303	27.362	0.026
	6 Gy	5min	24.946	0.708	0.502	0.236	24.402	25.491	0.028
		60min	26.624	1.103	1.216	0.368	25.777	27.472	0.041
		5hr	26.698	1.013	1.025	0.338	25.920	27.477	0.038
		24hr	27.837	1.283	1.646	0.428	26.851	28.823	0.046
		48hr	26.443	1.806	3.262	0.602	25.055	27.832	0.068
**IPO8**	2 Gy	5min	25.045	1.259	1.584	0.420	24.078	26.013	0.050
		60min	24.865	1.081	1.168	0.360	24.034	25.696	0.043
		5hr	24.890	1.026	1.052	0.342	24.101	25.678	0.041
		24hr	26.422	0.379	0.144	0.126	26.131	26.714	0.014
		48hr	26.522	1.049	1.101	0.350	25.716	27.329	0.040
	4 Gy	5min	24.950	0.324	0.105	0.108	24.700	25.199	0.013
		60min	24.879	1.207	1.457	0.402	23.952	25.807	0.049
		5hr	24.916	0.632	0.400	0.211	24.430	25.402	0.025
		24hr	25.121	0.353	0.125	0.118	24.850	25.393	0.014
		48hr	26.223	0.952	0.906	0.317	25.491	26.955	0.036
	6 Gy	5min	23.760	0.448	0.201	0.149	23.415	24.104	0.019
		60min	24.980	1.024	1.048	0.341	24.193	25.767	0.041
		5hr	25.032	0.738	0.544	0.246	24.465	25.599	0.029
		24hr	25.965	1.168	1.364	0.389	25.067	26.863	0.045
		48hr	25.524	1.046	1.094	0.349	24.720	26.328	0.041
**PGK1**	2 Gy	5min	22.045	1.511	2.282	0.504	20.884	23.207	0.069
		60min	21.664	0.968	0.937	0.323	20.920	22.408	0.045
		5hr	21.288	0.847	0.717	0.282	20.637	21.939	0.040
		24hr	24.567	2.311	5.342	0.770	22.790	26.343	0.094
		48hr	24.152	0.647	0.418	0.216	23.655	24.650	0.027
	4 Gy	5min	20.979	0.473	0.224	0.158	20.616	21.343	0.023
		60min	21.274	0.881	0.776	0.294	20.597	21.952	0.041
		5hr	21.347	0.189	0.036	0.063	21.201	21.492	0.009
		24hr	21.176	0.534	0.285	0.178	20.766	21.587	0.025
		48hr	23.580	1.109	1.229	0.370	22.728	24.433	0.047
	6 Gy	5min	20.704	0.380	0.144	0.127	20.412	20.996	0.018
		60min	21.663	0.987	0.974	0.329	20.904	22.422	0.046
		5hr	22.172	0.918	0.844	0.306	21.466	22.878	0.041
		24hr	24.197	2.064	4.259	0.688	22.610	25.783	0.085
		48hr	23.647	1.501	2.254	0.500	22.493	24.801	0.063
**PP1A**	2 Gy	5min	17.247	0.534	0.285	0.178	16.836	17.658	0.031
		60min	17.224	0.680	0.463	0.227	16.701	17.747	0.039
		5hr	16.982	0.477	0.227	0.159	16.615	17.348	0.028
		24hr	18.783	0.456	0.208	0.152	18.432	19.133	0.024
		48hr	18.432	0.225	0.051	0.075	18.259	18.605	0.012
	4 Gy	5min	16.743	0.420	0.176	0.140	16.420	17.065	0.025
		60min	16.601	0.686	0.470	0.229	16.074	17.128	0.041
		5hr	16.630	0.429	0.184	0.143	16.300	16.960	0.026
		24hr	17.223	0.362	0.131	0.121	16.945	17.502	0.021
		48hr	19.049	1.314	1.727	0.438	18.039	20.060	0.069
	6 Gy	5min	16.608	0.396	0.157	0.132	16.304	16.913	0.024
		60min	17.103	0.767	0.589	0.256	16.513	17.693	0.045
		5hr	17.493	0.520	0.270	0.173	17.093	17.892	0.030
		24hr	18.292	0.291	0.085	0.097	18.068	18.515	0.016
		48hr	19.193	1.007	1.014	0.336	18.419	19.967	0.052
**HMBS**	2 Gy	5min	31.421	1.481	2.193	0.494	30.283	32.559	0.047
		60min	31.312	0.930	0.865	0.310	30.598	32.027	0.030
		5hr	31.006	1.108	1.227	0.369	30.155	31.857	0.036
		24hr	31.704	0.790	0.623	0.263	31.097	32.311	0.025
		48hr	32.661	0.811	0.657	0.270	32.038	33.284	0.025
	4 Gy	5min	30.987	0.384	0.148	0.128	30.692	31.282	0.012
		60min	31.234	0.919	0.845	0.306	30.528	31.941	0.029
		5hr	30.926	0.675	0.455	0.225	30.408	31.445	0.022
		24hr	30.178	0.318	0.101	0.106	29.934	30.423	0.011
		48hr	31.512	0.446	0.199	0.149	31.169	31.855	0.014
	6 Gy	5min	30.640	0.581	0.337	0.194	30.194	31.087	0.019
		60min	31.717	0.832	0.692	0.277	31.078	32.357	0.026
		5hr	32.321	0.858	0.736	0.286	31.662	32.980	0.027
		24hr	32.058	1.395	1.945	0.465	30.985	33.130	0.044
		48hr	31.113	1.822	3.321	0.607	29.712	32.514	0.059
**GUSB**	2 Gy	5min	23.452	0.255	0.065	0.085	23.255	23.648	0.011
		60min	23.950	0.387	0.150	0.129	23.652	24.247	0.016
		5hr	23.380	0.420	0.177	0.140	23.057	23.703	0.018
		24hr	24.514	0.936	0.877	0.312	23.795	25.234	0.038
		48hr	23.406	0.288	0.083	0.096	23.185	23.628	0.012
	4 Gy	5min	23.813	0.352	0.124	0.117	23.543	24.084	0.015
		60min	23.504	0.302	0.091	0.101	23.272	23.735	0.013
		5hr	23.403	0.218	0.047	0.073	23.236	23.571	0.009
		24hr	23.183	0.422	0.178	0.141	22.859	23.508	0.018
		48hr	23.193	1.170	1.368	0.390	22.294	24.092	0.050
	6 Gy	5min	23.181	0.443	0.197	0.148	22.840	23.522	0.019
		60min	23.514	0.844	0.712	0.281	22.865	24.163	0.036
		5hr	24.011	0.336	0.113	0.112	23.753	24.269	0.014
		24hr	24.131	0.489	0.239	0.163	23.756	24.507	0.020
		48hr	23.534	1.691	2.861	0.564	22.234	24.834	0.072
**UBC**	2 Gy	5min	20.973	0.751	0.564	0.250	20.396	21.550	0.036
		60min	20.839	0.594	0.353	0.198	20.382	21.296	0.029
		5hr	20.415	0.801	0.642	0.267	19.799	21.031	0.039
		24hr	22.021	1.142	1.303	0.381	21.143	22.899	0.052
		48hr	22.282	0.647	0.419	0.216	21.784	22.780	0.029
	4 Gy	5min	20.752	0.868	0.753	0.289	20.085	21.419	0.042
		60min	21.343	0.616	0.379	0.205	20.869	21.816	0.029
		5hr	21.255	0.369	0.136	0.123	20.972	21.539	0.017
		24hr	21.139	0.085	0.007	0.028	21.074	21.205	0.004
		48hr	21.395	0.543	0.295	0.181	20.977	21.812	0.025
	6 Gy	5min	20.226	0.179	0.032	0.060	20.088	20.364	0.009
		60min	20.738	0.827	0.684	0.276	20.102	21.374	0.040
		5hr	21.791	0.367	0.135	0.122	21.509	22.073	0.017
		24hr	21.696	1.036	1.074	0.345	20.899	22.492	0.048
		48hr	21.448	0.542	0.294	0.181	21.031	21.864	0.025

SCC-6			Mean	Standard Deviation	Variance	SE of mean	Lower 95% CI of Mean	Upper 95% CI of Mean	Coefficient of Variation
**YWHAZ**	2 Gy	5min	25.114	1.000	1.000	0.333	24.345	25.882	0.040
		60min	25.022	0.728	0.530	0.243	24.462	25.582	0.029
		5hr	24.709	0.559	0.312	0.186	24.280	25.139	0.023
		24hr	25.921	0.750	0.563	0.250	25.345	26.498	0.029
		48hr	26.539	0.625	0.391	0.208	26.059	27.020	0.024
	4 Gy	5min	25.125	0.653	0.426	0.218	24.623	25.627	0.026
		60min	25.587	0.592	0.350	0.197	25.132	26.042	0.023
		5hr	25.763	0.315	0.099	0.105	25.520	26.005	0.012
		24hr	25.436	0.138	0.019	0.046	25.330	25.542	0.005
		48hr	24.999	0.763	0.582	0.254	24.413	25.586	0.031
	6 Gy	5min	24.574	0.230	0.053	0.077	24.397	24.751	0.009
		60min	25.063	0.925	0.856	0.308	24.352	25.774	0.037
		5hr	25.730	0.151	0.023	0.050	25.613	25.846	0.006
		24hr	25.092	0.933	0.870	0.311	24.376	25.809	0.037
		48hr	25.888	0.180	0.032	0.060	25.749	26.026	0.007
**GAPDH**	2 Gy	5min	18.791	1.565	2.449	0.522	17.588	19.994	0.083
		60min	18.117	0.808	0.653	0.269	17.496	18.738	0.045
		5hr	17.971	0.520	0.270	0.173	17.571	18.370	0.029
		24hr	19.560	0.727	0.528	0.242	19.001	20.118	0.037
		48hr	20.400	0.506	0.256	0.169	20.011	20.789	0.025
	4 Gy	5min	17.535	0.446	0.199	0.149	17.192	17.878	0.025
		60min	17.804	1.009	1.018	0.336	17.029	18.580	0.057
		5hr	17.879	0.585	0.342	0.195	17.429	18.329	0.033
		24hr	17.469	0.248	0.062	0.083	17.278	17.660	0.014
		48hr	19.172	0.740	0.547	0.247	18.603	19.740	0.039
	6 Gy	5min	17.418	0.608	0.369	0.203	16.951	17.885	0.035
		60min	18.731	1.130	1.278	0.377	17.862	19.600	0.060
		5hr	18.677	1.170	1.369	0.390	17.777	19.576	0.063
		24hr	19.694	2.094	4.384	0.698	18.084	21.303	0.106
		48hr	19.688	1.429	2.043	0.476	18.589	20.787	0.073
**HPRT1**	2 Gy	5min	20.920	0.510	0.260	0.170	20.527	21.312	0.024
		60min	21.297	0.480	0.230	0.160	20.928	21.666	0.023
		5hr	21.253	0.174	0.030	0.058	21.119	21.387	0.008
		24hr	22.889	0.559	0.312	0.186	22.459	23.318	0.024
		48hr	22.058	0.743	0.551	0.248	21.487	22.629	0.034
	4 Gy	5min	20.923	0.925	0.856	0.308	20.212	21.634	0.044
		60min	20.919	1.122	1.258	0.374	20.057	21.781	0.054
		5hr	20.747	0.916	0.840	0.305	20.043	21.452	0.044
		24hr	21.694	0.255	0.065	0.085	21.498	21.890	0.012
		48hr	22.500	1.032	1.066	0.344	21.706	23.293	0.046
	6 Gy	5min	20.697	1.081	1.169	0.360	19.866	21.528	0.052
		60min	21.698	0.497	0.247	0.166	21.316	22.080	0.023
		5hr	21.678	0.973	0.946	0.324	20.930	22.425	0.045
		24hr	22.143	0.378	0.143	0.126	21.853	22.433	0.017
		48hr	22.462	1.718	2.950	0.573	21.142	23.783	0.076
**ACTB**	2 Gy	5min	21.808	1.463	2.141	0.488	20.683	22.932	0.067
		60min	21.417	0.609	0.371	0.203	20.949	21.885	0.028
		5hr	21.688	0.338	0.114	0.113	21.428	21.947	0.016
		24hr	24.515	0.809	0.654	0.270	23.893	25.137	0.033
		48hr	26.794	0.904	0.817	0.301	26.100	27.489	0.034
	4 Gy	5min	21.232	1.149	1.319	0.383	20.349	22.115	0.054
		60min	21.876	2.349	5.516	0.783	20.071	23.681	0.107
		5hr	22.249	1.630	2.658	0.543	20.996	23.503	0.073
		24hr	23.155	1.234	1.522	0.411	22.207	24.103	0.053
		48hr	25.031	0.788	0.621	0.263	24.425	25.637	0.031
	6 Gy	5min	21.483	1.024	1.049	0.341	20.696	22.270	0.048
		60min	21.846	0.671	0.450	0.224	21.331	22.361	0.031
		5hr	22.192	0.435	0.189	0.145	21.858	22.527	0.020
		24hr	25.067	1.659	2.751	0.553	23.792	26.342	0.066
		48hr	25.382	0.757	0.573	0.252	24.800	25.964	0.030
**B2M**	2 Gy	5min	17.682	0.818	0.670	0.273	17.052	18.311	0.046
		60min	18.595	0.380	0.144	0.127	18.303	18.887	0.020
		5hr	18.495	0.238	0.057	0.079	18.312	18.678	0.013
		24hr	19.649	0.720	0.519	0.240	19.095	20.203	0.037
		48hr	19.096	0.508	0.258	0.169	18.706	19.486	0.027
	4 Gy	5min	18.522	0.657	0.432	0.219	18.017	19.027	0.035
		60min	18.584	1.147	1.315	0.382	17.703	19.466	0.062
		5hr	18.367	0.645	0.416	0.215	17.871	18.863	0.035
		24hr	18.887	0.195	0.038	0.065	18.738	19.037	0.010
		48hr	19.340	0.863	0.744	0.288	18.676	20.003	0.045
	6 Gy	5min	18.879	0.291	0.085	0.097	18.655	19.103	0.015
		60min	19.000	0.811	0.657	0.270	18.377	19.624	0.043
		5hr	19.239	0.708	0.502	0.236	18.695	19.784	0.037
		24hr	19.615	0.428	0.183	0.143	19.286	19.944	0.022
		48hr	19.396	1.124	1.263	0.375	18.532	20.259	0.058
**TBP**	2 Gy	5min	25.013	0.491	0.241	0.164	24.636	25.390	0.020
		60min	25.141	0.825	0.680	0.275	24.507	25.775	0.033
		5hr	25.431	0.650	0.423	0.217	24.931	25.931	0.026
		24hr	25.855	1.133	1.284	0.378	24.984	26.726	0.044
		48hr	26.614	0.757	0.573	0.252	26.033	27.196	0.028
	4 Gy	5min	25.500	0.920	0.847	0.307	24.793	26.208	0.036
		60min	24.907	0.727	0.528	0.242	24.349	25.466	0.029
		5hr	25.293	0.420	0.176	0.140	24.971	25.616	0.017
		24hr	25.323	0.202	0.041	0.067	25.168	25.479	0.008
		48hr	25.429	0.748	0.560	0.249	24.854	26.004	0.029
	6 Gy	5min	24.564	0.427	0.183	0.142	24.236	24.893	0.017
		60min	25.613	0.767	0.588	0.256	25.024	26.203	0.030
		5hr	25.522	0.727	0.528	0.242	24.963	26.081	0.028
		24hr	25.999	1.159	1.342	0.386	25.109	26.890	0.045
		48hr	25.089	1.897	3.599	0.632	23.631	26.547	0.076
**TFRC**	2 Gy	5min	22.687	0.525	0.276	0.175	22.284	23.091	0.023
		60min	22.873	0.526	0.276	0.175	22.469	23.277	0.023
		5hr	23.161	0.608	0.370	0.203	22.693	23.628	0.026
		24hr	23.798	0.504	0.254	0.168	23.411	24.185	0.021
		48hr	24.976	1.210	1.464	0.403	24.046	25.906	0.048
	4 Gy	5min	22.779	0.263	0.069	0.088	22.576	22.981	0.012
		60min	22.493	1.364	1.862	0.455	21.445	23.542	0.061
		5hr	22.491	0.888	0.788	0.296	21.808	23.173	0.039
		24hr	23.047	0.244	0.060	0.081	22.859	23.234	0.011
		48hr	23.656	0.781	0.609	0.260	23.056	24.256	0.033
	6 Gy	5min	21.907	0.645	0.416	0.215	21.412	22.403	0.029
		60min	22.817	0.909	0.825	0.303	22.118	23.515	0.040
		5hr	23.097	0.445	0.198	0.148	22.755	23.439	0.019
		24hr	23.920	1.061	1.126	0.354	23.105	24.736	0.044
		48hr	23.258	1.685	2.839	0.562	21.963	24.553	0.072

Table S9. Mean Cq values, standard deviation (SD), standard error of mean, 95% confidence intervals, variance and coefficient of variation (CV) of 14 HKGs across SCC-1483 cells (head and neck cancer cell line) with various levels of radiation treatment (2 Gy, 4 Gy, and 6 Gy) and time post-irradiation (5 min, 1, 5, 24, and 48 h).

SCC-1483			Mean	Standard Deviation	Variance	SE of mean	Lower 95% CI of Mean	Upper 95% CI of Mean	Coefficient of Variation
**G6PD**	2 Gy	5min	24.298	2.569	6.600	0.856	22.323	26.272	0.106
		60min	23.489	1.502	2.256	0.501	22.335	24.644	0.064
		5hr	24.161	1.632	2.664	0.544	22.906	25.415	0.068
		24hr	23.077	1.076	1.157	0.359	22.250	23.904	0.047
		48hr	25.214	1.453	2.110	0.484	24.097	26.330	0.058
	4 Gy	5min	23.056	0.730	0.533	0.243	22.494	23.617	0.032
		60min	22.887	1.457	2.123	0.486	21.767	24.007	0.064
		5hr	23.750	1.641	2.694	0.547	22.489	25.012	0.069
		24hr	22.345	0.472	0.223	0.157	21.982	22.708	0.021
		48hr	24.009	0.793	0.630	0.264	23.399	24.619	0.033
	6 Gy	5min	24.312	1.748	3.054	0.583	22.969	25.655	0.072
		60min	23.308	1.113	1.239	0.371	22.453	24.164	0.048
		5hr	23.330	0.933	0.870	0.311	22.613	24.047	0.040
		24hr	23.547	1.521	2.312	0.507	22.378	24.715	0.065
		48hr	23.664	1.130	1.277	0.377	22.796	24.533	0.048
**IPO8**	2 Gy	5min	24.411	1.767	3.123	0.589	23.053	25.770	0.072
		60min	23.892	1.523	2.320	0.508	22.721	25.062	0.064
		5hr	23.866	1.418	2.010	0.473	22.777	24.956	0.059
		24hr	23.571	0.796	0.633	0.265	22.959	24.183	0.034
		48hr	25.024	0.844	0.712	0.281	24.375	25.673	0.034
	4 Gy	5min	23.410	0.959	0.919	0.320	22.674	24.147	0.041
		60min	23.333	1.395	1.945	0.465	22.261	24.405	0.060
		5hr	24.212	1.058	1.119	0.353	23.399	25.025	0.044
		24hr	22.965	0.257	0.066	0.086	22.767	23.162	0.011
		48hr	24.255	0.307	0.094	0.102	24.019	24.491	0.013
	6 Gy	5min	24.130	1.469	2.158	0.490	23.001	25.259	0.061
		60min	23.979	1.690	2.856	0.563	22.680	25.278	0.070
		5hr	23.679	0.570	0.325	0.190	23.240	24.117	0.024
		24hr	24.065	0.762	0.581	0.254	23.479	24.651	0.032
		48hr	24.318	0.521	0.272	0.174	23.918	24.719	0.021
**PGK1**	2 Gy	5min	20.622	1.482	2.195	0.494	19.484	21.761	0.072
		60min	19.796	1.634	2.671	0.545	18.540	21.053	0.083
		5hr	19.893	1.529	2.338	0.510	18.718	21.068	0.077
		24hr	20.220	0.756	0.572	0.252	19.638	20.801	0.037
		48hr	22.165	1.344	1.806	0.448	21.132	23.198	0.061
	4 Gy	5min	19.872	1.086	1.179	0.362	19.037	20.706	0.055
		60min	19.977	1.379	1.902	0.460	18.917	21.037	0.069
		5hr	20.675	1.250	1.563	0.417	19.715	21.636	0.060
		24hr	19.807	0.353	0.125	0.118	19.536	20.078	0.018
		48hr	21.046	0.595	0.354	0.198	20.589	21.504	0.028
	6 Gy	5min	20.146	1.263	1.596	0.421	19.175	21.117	0.063
		60min	20.109	1.403	1.970	0.468	19.031	21.188	0.070
		5hr	20.077	0.683	0.467	0.228	19.552	20.603	0.034
		24hr	20.907	0.696	0.485	0.232	20.371	21.442	0.033
		48hr	21.889	0.547	0.300	0.182	21.468	22.310	0.025
**PP1A**	2 Gy	5min	16.507	0.653	0.426	0.218	16.005	17.009	0.040
		60min	16.676	0.307	0.094	0.102	16.440	16.912	0.018
		5hr	16.511	0.875	0.765	0.292	15.838	17.183	0.053
		24hr	16.979	0.316	0.100	0.105	16.736	17.222	0.019
		48hr	17.810	1.624	2.638	0.541	16.562	19.059	0.091
	4 Gy	5min	16.435	0.733	0.537	0.244	15.872	16.999	0.045
		60min	16.511	0.976	0.952	0.325	15.761	17.261	0.059
		5hr	17.278	0.290	0.084	0.097	17.056	17.501	0.017
		24hr	16.747	0.436	0.190	0.145	16.412	17.082	0.026
		48hr	17.982	0.940	0.884	0.313	17.259	18.705	0.052
	6 Gy	5min	16.557	0.403	0.162	0.134	16.247	16.866	0.024
		60min	16.995	1.302	1.696	0.434	15.994	17.996	0.077
		5hr	16.585	0.205	0.042	0.068	16.428	16.742	0.012
		24hr	17.074	0.238	0.057	0.079	16.890	17.257	0.014
		48hr	17.785	0.892	0.795	0.297	17.099	18.470	0.050
**HMBS**	2 Gy	5min	30.076	2.126	4.521	0.709	28.442	31.710	0.071
		60min	29.201	1.600	2.562	0.533	27.971	30.431	0.055
		5hr	29.322	1.399	1.958	0.466	28.246	30.398	0.048
		24hr	28.743	0.670	0.449	0.223	28.228	29.258	0.023
		48hr	30.376	1.178	1.387	0.393	29.471	31.282	0.039
	4 Gy	5min	28.827	1.134	1.287	0.378	27.955	29.699	0.039
		60min	29.296	1.435	2.060	0.478	28.192	30.399	0.049
		5hr	29.434	1.220	1.487	0.407	28.497	30.372	0.041
		24hr	28.241	0.226	0.051	0.075	28.067	28.415	0.008
		48hr	29.157	0.549	0.301	0.183	28.735	29.578	0.019
	6 Gy	5min	29.232	1.342	1.801	0.447	28.200	30.263	0.046
		60min	29.201	1.277	1.632	0.426	28.219	30.183	0.044
		5hr	29.052	0.629	0.396	0.210	28.569	29.536	0.022
		24hr	29.739	0.931	0.866	0.310	29.024	30.455	0.031
		48hr	29.758	1.088	1.184	0.363	28.922	30.594	0.037
**GUSB**	2 Gy	5min	22.542	0.534	0.285	0.178	22.131	22.952	0.024
		60min	22.368	0.725	0.526	0.242	21.811	22.925	0.032
		5hr	22.586	0.802	0.643	0.267	21.969	23.202	0.036
		24hr	22.736	0.541	0.292	0.180	22.321	23.152	0.024
		48hr	22.960	1.183	1.400	0.394	22.050	23.869	0.052
	4 Gy	5min	22.503	0.299	0.089	0.100	22.273	22.732	0.013
		60min	22.644	0.821	0.674	0.274	22.013	23.275	0.036
		5hr	22.658	0.255	0.065	0.085	22.462	22.854	0.011
		24hr	22.260	0.453	0.205	0.151	21.912	22.608	0.020
		48hr	22.672	0.612	0.374	0.204	22.201	23.142	0.027
	6 Gy	5min	22.361	0.250	0.063	0.083	22.169	22.554	0.011
		60min	22.740	0.773	0.597	0.258	22.146	23.334	0.034
		5hr	22.273	0.344	0.119	0.115	22.008	22.538	0.015
		24hr	22.560	0.390	0.152	0.130	22.261	22.860	0.017
		48hr	22.916	0.327	0.107	0.109	22.664	23.167	0.014
**UBC**	2 Gy	5min	20.773	1.121	1.258	0.374	19.911	21.635	0.054
		60min	20.072	1.209	1.462	0.403	19.143	21.002	0.060
		5hr	20.386	1.303	1.698	0.434	19.384	21.387	0.064
		24hr	20.375	0.942	0.888	0.314	19.650	21.099	0.046
		48hr	21.099	0.645	0.416	0.215	20.603	21.594	0.031
	4 Gy	5min	20.136	0.959	0.920	0.320	19.399	20.874	0.048
		60min	20.336	1.486	2.207	0.495	19.194	21.478	0.073
		5hr	20.460	0.825	0.680	0.275	19.826	21.094	0.040
		24hr	20.129	0.714	0.509	0.238	19.581	20.678	0.035
		48hr	20.382	0.437	0.191	0.146	20.046	20.717	0.021
	6 Gy	5min	20.124	1.097	1.203	0.366	19.281	20.967	0.054
		60min	20.218	1.321	1.745	0.440	19.202	21.233	0.065
		5hr	19.941	0.510	0.260	0.170	19.549	20.333	0.026
		24hr	20.680	0.953	0.908	0.318	19.947	21.412	0.046
		48hr	20.786	0.238	0.057	0.079	20.603	20.969	0.011

SCC-1483			Mean	Standard Deviation	Variance	SE of mean	Lower 95% CI of Mean	Upper 95% CI of Mean	Coefficient of Variation
**YWHAZ**	2 Gy	5min	24.607	0.669	0.447	0.223	24.093	25.121	0.027
		60min	24.231	0.448	0.200	0.149	23.887	24.575	0.018
		5hr	24.804	0.505	0.255	0.168	24.416	25.192	0.020
		24hr	25.372	0.756	0.571	0.252	24.791	25.952	0.030
		48hr	25.546	1.041	1.084	0.347	24.746	26.346	0.041
	4 Gy	5min	24.295	0.578	0.334	0.193	23.851	24.740	0.024
		60min	24.538	0.603	0.363	0.201	24.075	25.001	0.025
		5hr	24.870	0.449	0.202	0.150	24.524	25.215	0.018
		24hr	24.813	0.191	0.036	0.064	24.666	24.959	0.008
		48hr	25.279	1.098	1.205	0.366	24.435	26.123	0.043
	6 Gy	5min	24.406	0.257	0.066	0.086	24.209	24.604	0.011
		60min	24.460	0.631	0.398	0.210	23.975	24.945	0.026
		5hr	24.511	0.296	0.087	0.099	24.284	24.738	0.012
		24hr	25.464	0.512	0.262	0.171	25.070	25.857	0.020
		48hr	25.488	1.304	1.699	0.435	24.486	26.490	0.051
**GAPDH**	2 Gy	5min	17.362	1.651	2.724	0.550	16.093	18.630	0.095
		60min	16.411	1.621	2.629	0.540	15.165	17.657	0.099
		5hr	17.049	1.440	2.074	0.480	15.942	18.156	0.084
		24hr	16.205	0.661	0.437	0.220	15.697	16.713	0.041
		48hr	18.312	1.619	2.620	0.540	17.067	19.556	0.088
	4 Gy	5min	16.333	1.115	1.242	0.372	15.476	17.190	0.068
		60min	17.045	2.307	5.322	0.769	15.272	18.818	0.135
		5hr	17.054	1.399	1.957	0.466	15.979	18.130	0.082
		24hr	15.957	0.621	0.385	0.207	15.480	16.434	0.039
		48hr	17.373	0.366	0.134	0.122	17.092	17.655	0.021
	6 Gy	5min	17.013	1.605	2.575	0.535	15.780	18.246	0.094
		60min	17.000	1.586	2.514	0.529	15.781	18.219	0.093
		5hr	16.829	0.878	0.771	0.293	16.154	17.504	0.052
		24hr	17.028	0.923	0.853	0.308	16.318	17.738	0.054
		48hr	17.483	0.715	0.512	0.238	16.933	18.032	0.041
**HPRT1**	2 Gy	5min	20.768	0.671	0.450	0.224	20.252	21.283	0.032
		60min	21.022	0.682	0.465	0.227	20.498	21.546	0.032
		5hr	21.255	0.830	0.688	0.277	20.617	21.893	0.039
		24hr	21.255	0.662	0.438	0.221	20.746	21.763	0.031
		48hr	22.314	1.334	1.780	0.445	21.288	23.340	0.060
	4 Gy	5min	20.455	0.869	0.756	0.290	19.786	21.123	0.043
		60min	20.235	0.366	0.134	0.122	19.954	20.516	0.018
		5hr	21.435	0.555	0.308	0.185	21.008	21.862	0.026
		24hr	21.357	0.643	0.413	0.214	20.863	21.851	0.030
		48hr	21.705	0.497	0.247	0.166	21.324	22.087	0.023
	6 Gy	5min	20.944	0.638	0.407	0.213	20.453	21.434	0.030
		60min	21.315	0.917	0.842	0.306	20.610	22.020	0.043
		5hr	21.139	0.472	0.223	0.157	20.776	21.502	0.022
		24hr	21.693	0.201	0.040	0.067	21.538	21.847	0.009
		48hr	22.096	0.451	0.203	0.150	21.749	22.443	0.020
**ACTB**	2 Gy	5min	20.647	2.113	4.466	0.704	19.023	22.271	0.102
		60min	19.579	2.239	5.014	0.746	17.858	21.301	0.114
		5hr	20.269	1.724	2.974	0.575	18.944	21.595	0.085
		24hr	20.151	1.290	1.665	0.430	19.159	21.143	0.064
		48hr	22.938	1.411	1.990	0.470	21.853	24.022	0.062
	4 Gy	5min	19.796	1.430	2.046	0.477	18.697	20.896	0.072
		60min	20.369	2.069	4.279	0.690	18.779	21.959	0.102
		5hr	20.035	1.890	3.572	0.630	18.582	21.488	0.094
		24hr	19.504	0.688	0.474	0.229	18.975	20.033	0.035
		48hr	21.263	0.321	0.103	0.107	21.017	21.510	0.015
	6 Gy	5min	20.222	1.890	3.571	0.630	18.769	21.674	0.093
		60min	19.528	1.404	1.970	0.468	18.450	20.607	0.072
		5hr	19.522	0.787	0.620	0.262	18.917	20.127	0.040
		24hr	20.988	1.532	2.346	0.511	19.811	22.166	0.073
		48hr	21.653	1.208	1.460	0.403	20.725	22.582	0.056
**B2M**	2 Gy	5min	19.005	0.393	0.155	0.131	18.702	19.307	0.021
		60min	19.168	0.700	0.490	0.233	18.630	19.706	0.037
		5hr	19.321	0.466	0.217	0.155	18.963	19.679	0.024
		24hr	18.759	0.458	0.210	0.153	18.407	19.111	0.024
		48hr	19.526	1.353	1.829	0.451	18.486	20.566	0.069
	4 Gy	5min	18.572	0.183	0.034	0.061	18.431	18.713	0.010
		60min	18.640	0.344	0.118	0.115	18.375	18.904	0.018
		5hr	19.492	0.431	0.186	0.144	19.160	19.823	0.022
		24hr	18.693	0.577	0.333	0.192	18.249	19.136	0.031
		48hr	19.458	0.658	0.433	0.219	18.953	19.964	0.034
	6 Gy	5min	18.980	0.284	0.081	0.095	18.761	19.198	0.015
		60min	19.522	0.938	0.879	0.313	18.802	20.243	0.048
		5hr	19.092	0.414	0.171	0.138	18.774	19.410	0.022
		24hr	18.966	0.360	0.130	0.120	18.690	19.243	0.019
		48hr	19.370	0.528	0.279	0.176	18.965	19.776	0.027
**TBP**	2 Gy	5min	25.011	1.511	2.283	0.504	23.850	26.173	0.060
		60min	24.337	1.177	1.385	0.392	23.432	25.242	0.048
		5hr	24.417	1.096	1.202	0.365	23.574	25.260	0.045
		24hr	24.194	0.802	0.643	0.267	23.577	24.810	0.033
		48hr	25.528	0.918	0.843	0.306	24.822	26.233	0.036
	4 Gy	5min	24.094	0.700	0.490	0.233	23.557	24.632	0.029
		60min	24.183	1.029	1.060	0.343	23.392	24.975	0.043
		5hr	24.251	0.775	0.600	0.258	23.655	24.846	0.032
		24hr	23.584	0.345	0.119	0.115	23.319	23.850	0.015
		48hr	24.565	0.239	0.057	0.080	24.381	24.749	0.010
	6 Gy	5min	24.298	0.930	0.864	0.310	23.584	25.013	0.038
		60min	24.382	0.681	0.464	0.227	23.858	24.905	0.028
		5hr	23.975	0.320	0.102	0.107	23.730	24.221	0.013
		24hr	24.623	0.812	0.659	0.271	23.999	25.247	0.033
		48hr	24.600	0.562	0.316	0.187	24.168	25.032	0.023
**TFRC**	2 Gy	5min	21.742	1.913	3.659	0.638	20.271	23.212	0.088
		60min	20.991	1.345	1.809	0.448	19.957	22.025	0.064
		5hr	21.194	1.439	2.071	0.480	20.088	22.300	0.068
		24hr	20.358	0.759	0.577	0.253	19.775	20.942	0.037
		48hr	22.485	1.131	1.280	0.377	21.615	23.354	0.050
	4 Gy	5min	20.224	0.567	0.322	0.189	19.788	20.660	0.028
		60min	20.422	1.023	1.047	0.341	19.635	21.208	0.050
		5hr	20.947	0.829	0.687	0.276	20.310	21.584	0.040
		24hr	19.804	0.289	0.083	0.096	19.582	20.025	0.015
		48hr	21.707	0.457	0.209	0.152	21.355	22.058	0.021
	6 Gy	5min	21.294	1.537	2.363	0.512	20.113	22.476	0.072
		60min	20.850	1.210	1.464	0.403	19.920	21.780	0.058
		5hr	20.649	0.733	0.537	0.244	20.086	21.213	0.036
		24hr	20.994	0.950	0.902	0.317	20.264	21.725	0.045
		48hr	21.793	0.762	0.581	0.254	21.208	22.379	0.035

Table S10. Mean Cq values, standard deviation (SD), standard error of mean, 95% confidence intervals, variance and coefficient of variation (CV) of 14 HKGs across A549 cells (lung cancer cell line) with various levels of radiation treatment (2 Gy, 4 Gy, and 6 Gy) and time post-irradiation (5 min, 1, 5, 24, and 48 h).

A549			Mean	Standard Deviation	Variance	SE of mean	Lower 95% CI of Mean	Upper 95% CI of Mean	Coefficient of Variation
G6PD	2 Gy	5min	22.007	0.216	0.047	0.072	21.841	22.173	0.010
		60min	23.177	1.461	2.134	0.487	22.055	24.300	0.063
		5hr	22.120	0.322	0.104	0.107	21.873	22.368	0.015
		24hr	22.116	1.168	1.364	0.389	21.218	23.013	0.053
		48hr	21.766	1.954	3.818	0.651	20.264	23.268	0.090
	4 Gy	5min	21.812	0.293	0.086	0.098	21.587	22.037	0.013
		60min	23.125	0.549	0.302	0.183	22.703	23.547	0.024
		5hr	22.586	0.399	0.159	0.133	22.279	22.892	0.018
		24hr	22.090	1.771	3.136	0.590	20.729	23.452	0.080
		48hr	22.719	1.409	1.985	0.470	21.636	23.802	0.062
	6 Gy	5min	22.095	0.474	0.225	0.158	21.731	22.460	0.021
		60min	23.296	1.613	2.600	0.538	22.057	24.536	0.069
		5hr	22.359	0.412	0.170	0.137	22.042	22.676	0.018
		24hr	22.217	0.994	0.988	0.331	21.453	22.981	0.045
		48hr	21.324	1.395	1.946	0.465	20.252	22.397	0.065
IPO8	2 Gy	5min	24.702	0.539	0.291	0.180	24.288	25.117	0.022
		60min	24.676	0.614	0.377	0.205	24.205	25.148	0.025
		5hr	24.264	0.897	0.804	0.299	23.575	24.953	0.037
		24hr	25.352	0.903	0.815	0.301	24.658	26.046	0.036
		48hr	24.492	0.446	0.199	0.149	24.150	24.835	0.018
	4 Gy	5min	24.859	0.649	0.421	0.216	24.360	25.357	0.026
		60min	25.974	0.333	0.111	0.111	25.718	26.230	0.013
		5hr	25.026	0.836	0.698	0.279	24.384	25.668	0.033
		24hr	25.413	1.368	1.872	0.456	24.361	26.464	0.054
		48hr	25.805	1.067	1.139	0.356	24.985	26.626	0.041
	6 Gy	5min	25.287	0.645	0.417	0.215	24.791	25.783	0.026
		60min	24.581	0.533	0.284	0.178	24.171	24.991	0.022
		5hr	25.094	0.336	0.113	0.112	24.836	25.353	0.013
		24hr	26.397	0.496	0.246	0.165	26.015	26.778	0.019
		48hr	24.386	0.665	0.442	0.222	23.875	24.897	0.027
PGK1	2 Gy	5min	21.057	1.557	2.423	0.519	19.861	22.254	0.074
		60min	20.700	0.861	0.742	0.287	20.037	21.362	0.042
		5hr	20.822	0.923	0.852	0.308	20.113	21.532	0.044
		24hr	21.178	0.536	0.287	0.179	20.767	21.590	0.025
		48hr	20.259	0.384	0.147	0.128	19.964	20.554	0.019
	4 Gy	5min	20.961	0.366	0.134	0.122	20.680	21.243	0.017
		60min	21.261	0.543	0.295	0.181	20.844	21.679	0.026
		5hr	21.425	0.462	0.213	0.154	21.070	21.780	0.022
		24hr	21.271	1.925	3.705	0.642	19.792	22.751	0.090
		48hr	21.123	1.071	1.147	0.357	20.300	21.946	0.051
	6 Gy	5min	21.841	1.073	1.151	0.358	21.017	22.666	0.049
		60min	21.038	0.896	0.803	0.299	20.349	21.727	0.043
		5hr	20.617	0.359	0.129	0.120	20.340	20.893	0.017
		24hr	21.096	0.777	0.604	0.259	20.498	21.693	0.037
		48hr	20.049	0.851	0.724	0.284	19.395	20.704	0.042
PP1A	2 Gy	5min	18.258	1.133	1.283	0.378	17.388	19.129	0.062
		60min	17.989	0.699	0.489	0.233	17.451	18.527	0.039
		5hr	17.988	0.979	0.958	0.326	17.236	18.741	0.054
		24hr	19.095	0.454	0.206	0.151	18.746	19.444	0.024
		48hr	17.475	0.564	0.318	0.188	17.041	17.908	0.032
	4 Gy	5min	18.335	1.104	1.219	0.368	17.486	19.184	0.060
		60min	19.064	0.927	0.859	0.309	18.352	19.777	0.049
		5hr	18.157	1.231	1.515	0.410	17.211	19.103	0.068
		24hr	19.173	0.748	0.559	0.249	18.598	19.748	0.039
		48hr	19.175	1.236	1.529	0.412	18.225	20.125	0.064
	6 Gy	5min	18.683	0.894	0.799	0.298	17.996	19.369	0.048
		60min	18.571	0.465	0.216	0.155	18.214	18.928	0.025
		5hr	17.813	1.414	1.999	0.471	16.726	18.900	0.079
		24hr	19.107	0.705	0.496	0.235	18.565	19.648	0.037
		48hr	18.916	0.661	0.437	0.220	18.408	19.424	0.035
HMBS	2 Gy	5min	29.950	1.214	1.474	0.405	29.017	30.883	0.041
		60min	30.441	0.503	0.253	0.168	30.054	30.827	0.017
		5hr	30.222	0.328	0.108	0.109	29.970	30.475	0.011
		24hr	29.874	1.137	1.292	0.379	29.000	30.748	0.038
		48hr	29.058	1.073	1.151	0.358	28.234	29.883	0.037
	4 Gy	5min	30.128	0.652	0.425	0.217	29.627	30.630	0.022
		60min	29.986	0.494	0.244	0.165	29.606	30.366	0.016
		5hr	29.831	0.294	0.087	0.098	29.605	30.057	0.010
		24hr	29.035	1.507	2.271	0.502	27.876	30.193	0.052
		48hr	30.091	0.957	0.915	0.319	29.355	30.826	0.032
	6 Gy	5min	30.540	1.066	1.136	0.355	29.721	31.360	0.035
		60min	30.163	0.845	0.714	0.282	29.514	30.813	0.028
		5hr	29.486	0.566	0.320	0.189	29.051	29.921	0.019
		24hr	29.858	0.768	0.590	0.256	29.268	30.448	0.026
		48hr	29.509	0.645	0.416	0.215	29.013	30.005	0.022
GUSB	2 Gy	5min	23.446	0.224	0.050	0.075	23.274	23.618	0.010
		60min	23.215	0.778	0.606	0.259	22.616	23.813	0.034
		5hr	23.788	0.285	0.081	0.095	23.569	24.007	0.012
		24hr	24.501	0.358	0.128	0.119	24.226	24.776	0.015
		48hr	23.425	0.491	0.241	0.164	23.047	23.802	0.021
	4 Gy	5min	23.330	1.083	1.172	0.361	22.498	24.162	0.046
		60min	23.814	0.472	0.223	0.157	23.451	24.176	0.020
		5hr	23.323	0.642	0.412	0.214	22.829	23.816	0.028
		24hr	23.659	0.833	0.693	0.278	23.019	24.299	0.035
		48hr	23.476	0.284	0.081	0.095	23.257	23.695	0.012
	6 Gy	5min	23.809	0.597	0.356	0.199	23.350	24.268	0.025
		60min	23.733	0.334	0.111	0.111	23.477	23.990	0.014
		5hr	23.686	0.327	0.107	0.109	23.435	23.938	0.014
		24hr	24.299	0.559	0.312	0.186	23.870	24.729	0.023
		48hr	23.948	0.168	0.028	0.056	23.819	24.077	0.007
UBC	2 Gy	5min	22.233	0.975	0.950	0.325	21.484	22.982	0.044
		60min	22.088	0.637	0.406	0.212	21.599	22.578	0.029
		5hr	22.558	0.283	0.080	0.094	22.340	22.775	0.013
		24hr	22.722	1.069	1.142	0.356	21.901	23.544	0.047
		48hr	21.271	1.253	1.571	0.418	20.308	22.235	0.059
	4 Gy	5min	21.874	0.602	0.362	0.201	21.411	22.337	0.028
		60min	22.206	0.475	0.226	0.158	21.840	22.571	0.021
		5hr	22.003	0.308	0.095	0.103	21.766	22.239	0.014
		24hr	21.668	1.784	3.184	0.595	20.296	23.039	0.082
		48hr	20.709	1.197	1.433	0.399	19.789	21.629	0.058
	6 Gy	5min	22.555	0.496	0.246	0.165	22.174	22.936	0.022
		60min	22.102	1.494	2.232	0.498	20.953	23.250	0.068
		5hr	22.152	0.673	0.453	0.224	21.634	22.669	0.030
		24hr	22.087	1.163	1.352	0.388	21.193	22.981	0.053
		48hr	21.303	0.683	0.466	0.228	20.778	21.828	0.032

A549			Mean	Standard Deviation	Variance	SE of mean	Lower 95% CI of Mean	Upper 95% CI of Mean	Coefficient of Variation
YWHAZ	2 Gy	5min	26.127	1.041	1.085	0.347	25.326	26.927	0.040
		60min	26.143	0.674	0.454	0.225	25.625	26.661	0.026
		5hr	26.282	0.320	0.103	0.107	26.036	26.529	0.012
		24hr	26.946	1.319	1.739	0.440	25.932	27.960	0.049
		48hr	25.770	1.753	3.072	0.584	24.422	27.117	0.068
	4 Gy	5min	26.138	1.276	1.627	0.425	25.158	27.119	0.049
		60min	26.400	1.110	1.233	0.370	25.547	27.254	0.042
		5hr	26.225	0.955	0.912	0.318	25.491	26.959	0.036
		24hr	25.439	2.036	4.145	0.679	23.874	27.004	0.080
		48hr	25.174	1.918	3.679	0.639	23.700	26.648	0.076
	6 Gy	5min	27.205	0.535	0.286	0.178	26.795	27.616	0.020
		60min	25.523	0.121	0.015	0.040	25.430	25.616	0.005
		5hr	26.061	0.286	0.082	0.095	25.841	26.282	0.011
		24hr	26.749	1.400	1.959	0.467	25.674	27.825	0.052
		48hr	25.676	1.412	1.993	0.471	24.591	26.761	0.055
GAPDH	2 Gy	5min	17.925	0.777	0.603	0.259	17.328	18.522	0.043
		60min	17.677	0.563	0.317	0.188	17.244	18.109	0.032
		5hr	17.078	0.391	0.153	0.130	16.778	17.379	0.023
		24hr	16.861	1.346	1.811	0.449	15.827	17.896	0.080
		48hr	16.715	1.765	3.117	0.588	15.358	18.072	0.106
	4 Gy	5min	17.825	1.106	1.222	0.369	16.975	18.674	0.062
		60min	17.062	0.648	0.420	0.216	16.564	17.560	0.038
		5hr	17.131	0.439	0.193	0.146	16.794	17.468	0.026
		24hr	17.474	1.897	3.600	0.632	16.015	18.932	0.109
		48hr	17.447	0.926	0.858	0.309	16.735	18.159	0.053
	6 Gy	5min	17.411	1.266	1.602	0.422	16.439	18.384	0.073
		60min	17.412	0.733	0.537	0.244	16.849	17.975	0.042
		5hr	16.360	0.682	0.465	0.227	15.835	16.884	0.042
		24hr	17.136	1.468	2.156	0.489	16.007	18.264	0.086
		48hr	16.945	1.071	1.146	0.357	16.122	17.768	0.063
HPRT1	2 Gy	5min	20.867	0.578	0.334	0.193	20.422	21.311	0.028
		60min	21.231	0.357	0.127	0.119	20.957	21.505	0.017
		5hr	20.868	0.158	0.025	0.053	20.747	20.989	0.008
		24hr	21.524	1.339	1.794	0.446	20.494	22.553	0.062
		48hr	21.338	1.066	1.137	0.356	20.518	22.158	0.050
	4 Gy	5min	21.344	0.530	0.281	0.177	20.936	21.751	0.025
		60min	21.147	0.362	0.131	0.121	20.869	21.425	0.017
		5hr	21.181	0.321	0.103	0.107	20.934	21.428	0.015
		24hr	21.885	0.877	0.770	0.292	21.211	22.559	0.040
		48hr	21.786	0.481	0.232	0.160	21.416	22.156	0.022
	6 Gy	5min	21.865	0.423	0.179	0.141	21.541	22.190	0.019
		60min	21.386	0.248	0.061	0.083	21.196	21.576	0.012
		5hr	21.190	0.213	0.045	0.071	21.027	21.354	0.010
		24hr	21.794	0.880	0.775	0.293	21.117	22.470	0.040
		48hr	22.069	0.424	0.180	0.141	21.743	22.395	0.019
ACTB	2 Gy	5min	21.685	0.746	0.556	0.249	21.111	22.258	0.034
		60min	22.093	2.213	4.899	0.738	20.392	23.794	0.100
		5hr	19.484	2.442	5.962	0.814	17.607	21.361	0.125
		24hr	19.905	2.227	4.959	0.742	18.194	21.617	0.112
		48hr	22.233	3.136	9.836	1.045	19.822	24.644	0.141
	4 Gy	5min	22.748	0.396	0.157	0.132	22.443	23.052	0.017
		60min	23.318	0.942	0.887	0.314	22.594	24.042	0.040
		5hr	22.769	0.813	0.661	0.271	22.144	23.394	0.036
		24hr	21.945	1.662	2.762	0.554	20.668	23.223	0.076
		48hr	24.125	2.468	6.092	0.823	22.228	26.023	0.102
	6 Gy	5min	22.436	0.906	0.821	0.302	21.739	23.132	0.040
		60min	21.726	1.550	2.402	0.517	20.534	22.917	0.071
		5hr	22.333	0.605	0.367	0.202	21.868	22.798	0.027
		24hr	22.853	1.840	3.385	0.613	21.439	24.267	0.081
		48hr	23.007	2.341	5.480	0.780	21.208	24.806	0.102
B2M	2 Gy	5min	18.771	0.727	0.529	0.242	18.212	19.330	0.039
		60min	19.068	0.649	0.421	0.216	18.569	19.567	0.034
		5hr	19.177	1.150	1.322	0.383	18.293	20.061	0.060
		24hr	19.866	0.220	0.049	0.074	19.697	20.036	0.011
		48hr	18.911	1.214	1.474	0.405	17.978	19.844	0.064
	4 Gy	5min	19.163	0.903	0.815	0.301	18.469	19.856	0.047
		60min	19.465	1.199	1.439	0.400	18.543	20.387	0.062
		5hr	18.861	0.643	0.413	0.214	18.367	19.355	0.034
		24hr	19.978	0.730	0.532	0.243	19.417	20.539	0.037
		48hr	19.434	0.928	0.862	0.309	18.721	20.148	0.048
	6 Gy	5min	19.757	0.466	0.217	0.155	19.399	20.116	0.024
		60min	19.331	0.653	0.426	0.218	18.829	19.832	0.034
		5hr	19.704	1.077	1.161	0.359	18.876	20.532	0.055
		24hr	19.512	0.437	0.191	0.146	19.176	19.848	0.022
		48hr	18.934	0.491	0.242	0.164	18.556	19.312	0.026
TBP	2 Gy	5min	26.191	0.955	0.912	0.318	25.457	26.925	0.036
		60min	26.395	0.546	0.298	0.182	25.975	26.814	0.021
		5hr	25.746	0.558	0.311	0.186	25.317	26.175	0.022
		24hr	26.508	0.787	0.619	0.262	25.903	27.113	0.030
		48hr	25.688	0.467	0.218	0.156	25.330	26.047	0.018
	4 Gy	5min	26.352	0.878	0.771	0.293	25.676	27.027	0.033
		60min	26.324	0.697	0.486	0.232	25.789	26.860	0.026
		5hr	26.275	0.748	0.560	0.249	25.700	26.851	0.028
		24hr	25.937	1.171	1.372	0.390	25.037	26.838	0.045
		48hr	26.181	0.966	0.933	0.322	25.439	26.924	0.037
	6 Gy	5min	26.367	0.546	0.299	0.182	25.947	26.787	0.021
		60min	25.920	0.458	0.209	0.153	25.568	26.271	0.018
		5hr	25.554	0.586	0.343	0.195	25.104	26.004	0.023
		24hr	26.827	0.427	0.182	0.142	26.499	27.155	0.016
		48hr	25.962	0.465	0.216	0.155	25.605	26.319	0.018
TFRC	2 Gy	5min	23.606	0.610	0.372	0.203	23.137	24.074	0.026
		60min	24.040	1.034	1.069	0.345	23.245	24.835	0.043
		5hr	23.176	0.350	0.123	0.117	22.907	23.445	0.015
		24hr	23.896	0.815	0.664	0.272	23.269	24.522	0.034
		48hr	23.718	1.408	1.982	0.469	22.636	24.800	0.059
	4 Gy	5min	23.883	0.967	0.936	0.322	23.140	24.627	0.041
		60min	23.945	1.133	1.284	0.378	23.074	24.816	0.047
		5hr	24.046	0.723	0.523	0.241	23.490	24.602	0.030
		24hr	23.801	0.985	0.971	0.328	23.043	24.558	0.041
		48hr	24.015	0.852	0.727	0.284	23.360	24.671	0.035
	6 Gy	5min	23.901	0.298	0.089	0.099	23.672	24.130	0.012
		60min	23.205	0.307	0.094	0.102	22.969	23.440	0.013
		5hr	23.228	0.206	0.042	0.069	23.070	23.387	0.009
		24hr	25.084	0.412	0.170	0.137	24.767	25.400	0.016
		48hr	23.768	1.115	1.243	0.372	22.911	24.625	0.047

Table S11. Mean Cq values, standard deviation (SD), standard error of mean, 95% confidence intervals, variance and coefficient of variation (CV) of 14 HKGs across NCI-H226 cells (lung cancer cell line) with various levels of radiation treatment (2 Gy, 4 Gy, and 6 Gy) and time post-irradiation (5 min, 1, 5, 24, and 48 h).

NCI-H226			Mean	Standard Deviation	Variance	SE of mean	Lower 95% CI of Mean	Upper 95% CI of Mean	Coefficient of Variation
G6PD	2 Gy	5min	24.269	0.974	0.948	0.397	23.247	25.290	0.040
		60min	24.328	0.844	0.712	0.344	23.443	25.213	0.035
		5hr	27.600	0.142	0.020	0.058	27.451	27.749	0.005
		24hr	27.491	1.666	2.777	0.680	25.742	29.240	0.061
		48hr	27.616	0.635	0.403	0.259	26.949	28.282	0.023
	4 Gy	5min	24.342	0.725	0.526	0.296	23.581	25.103	0.030
		60min	27.603	0.121	0.015	0.049	27.477	27.730	0.004
		5hr	25.001	0.394	0.155	0.161	24.587	25.415	0.016
		24hr	26.968	0.150	0.023	0.061	26.811	27.126	0.006
		48hr	25.791	0.618	0.382	0.252	25.142	26.440	0.024
	6 Gy	5min	25.665	1.970	3.879	0.804	23.598	27.732	0.077
		60min	24.654	0.142	0.020	0.058	24.505	24.804	0.006
		5hr	23.926	0.686	0.471	0.280	23.206	24.646	0.029
		24hr	25.631	0.990	0.981	0.404	24.592	26.670	0.039
		48hr	26.440	0.089	0.008	0.036	26.346	26.533	0.003
IPO8	2 Gy	5min	23.809	0.269	0.072	0.110	23.528	24.091	0.011
		60min	23.908	0.568	0.322	0.232	23.313	24.504	0.024
		5hr	26.955	0.420	0.177	0.172	26.514	27.396	0.016
		24hr	26.472	1.665	2.773	0.680	24.725	28.220	0.063
		48hr	26.031	0.860	0.739	0.351	25.129	26.933	0.033
	4 Gy	5min	24.382	0.963	0.928	0.393	23.372	25.393	0.040
		60min	26.418	0.133	0.018	0.054	26.279	26.557	0.005
		5hr	24.233	0.148	0.022	0.061	24.078	24.389	0.006
		24hr	26.118	0.922	0.850	0.376	25.150	27.085	0.035
		48hr	25.709	0.751	0.564	0.306	24.921	26.497	0.029
	6 Gy	5min	24.656	1.547	2.393	0.632	23.032	26.279	0.063
		60min	23.987	0.117	0.014	0.048	23.864	24.109	0.005
		5hr	23.940	0.362	0.131	0.148	23.559	24.320	0.015
		24hr	25.421	1.317	1.734	0.538	24.039	26.803	0.052
		48hr	25.932	0.076	0.006	0.031	25.852	26.012	0.003
PGK1	2 Gy	5min	20.303	0.445	0.198	0.182	19.836	20.769	0.022
		60min	20.344	0.649	0.421	0.265	19.662	21.025	0.032
		5hr	23.196	1.187	1.410	0.485	21.950	24.442	0.051
		24hr	23.828	2.204	4.856	0.900	21.515	26.140	0.092
		48hr	22.607	1.084	1.176	0.443	21.469	23.745	0.048
	4 Gy	5min	20.498	0.752	0.565	0.307	19.709	21.286	0.037
		60min	23.133	0.931	0.866	0.380	22.156	24.109	0.040
		5hr	20.526	0.256	0.065	0.104	20.258	20.795	0.012
		24hr	23.408	1.216	1.479	0.496	22.132	24.684	0.052
		48hr	21.551	0.622	0.386	0.254	20.899	22.204	0.029
	6 Gy	5min	22.219	3.027	9.164	1.236	19.042	25.395	0.136
		60min	20.197	0.781	0.610	0.319	19.378	21.017	0.039
		5hr	20.263	0.524	0.274	0.214	19.714	20.813	0.026
		24hr	22.641	1.763	3.108	0.720	20.791	24.491	0.078
		48hr	21.755	0.101	0.010	0.041	21.649	21.861	0.005
PP1A	2 Gy	5min	17.232	0.394	0.155	0.161	16.818	17.646	0.023
		60min	17.571	0.550	0.303	0.225	16.993	18.149	0.031
		5hr	19.293	1.808	3.270	0.738	17.396	21.191	0.094
		24hr	20.368	1.188	1.410	0.485	19.122	21.615	0.058
		48hr	18.299	0.128	0.016	0.052	18.164	18.433	0.007
	4 Gy	5min	17.510	0.916	0.840	0.374	16.548	18.472	0.052
		60min	17.835	0.256	0.066	0.105	17.567	18.104	0.014
		5hr	17.364	0.517	0.267	0.211	16.822	17.906	0.030
		24hr	20.775	1.503	2.260	0.614	19.197	22.352	0.072
		48hr	18.229	0.242	0.059	0.099	17.975	18.483	0.013
	6 Gy	5min	17.705	0.617	0.381	0.252	17.057	18.353	0.035
		60min	17.387	0.551	0.303	0.225	16.809	17.964	0.032
		5hr	17.231	0.779	0.607	0.318	16.413	18.049	0.045
		24hr	20.729	1.519	2.308	0.620	19.134	22.323	0.073
		48hr	18.089	0.130	0.017	0.053	17.952	18.226	0.007
HMBS	2 Gy	5min	29.198	0.176	0.031	0.072	29.013	29.382	0.006
		60min	29.148	0.419	0.175	0.171	28.709	29.587	0.014
		5hr	32.221	0.756	0.572	0.309	31.427	33.014	0.023
		24hr	29.924	0.941	0.885	0.384	28.937	30.912	0.031
		48hr	31.684	0.825	0.681	0.337	30.818	32.550	0.026
	4 Gy	5min	29.422	0.519	0.269	0.212	28.877	29.966	0.018
		60min	32.018	0.600	0.360	0.245	31.389	32.648	0.019
		5hr	29.656	0.368	0.135	0.150	29.270	30.042	0.012
		24hr	31.610	0.290	0.084	0.118	31.306	31.914	0.009
		48hr	30.064	0.223	0.050	0.091	29.830	30.299	0.007
	6 Gy	5min	31.146	2.772	7.685	1.132	28.236	34.055	0.089
		60min	29.308	0.161	0.026	0.066	29.139	29.478	0.006
		5hr	29.218	0.250	0.062	0.102	28.956	29.480	0.009
		24hr	30.503	0.716	0.513	0.292	29.752	31.255	0.023
		48hr	30.185	0.234	0.055	0.095	29.940	30.431	0.008
GUSB	2 Gy	5min	23.460	0.461	0.212	0.188	22.976	23.943	0.020
		60min	23.573	0.790	0.624	0.322	22.744	24.402	0.034
		5hr	25.193	1.688	2.849	0.689	23.422	26.965	0.067
		24hr	25.625	0.650	0.423	0.265	24.943	26.308	0.025
		48hr	23.907	0.095	0.009	0.039	23.807	24.008	0.004
	4 Gy	5min	23.706	0.600	0.360	0.245	23.076	24.335	0.025
		60min	23.943	0.244	0.059	0.099	23.687	24.199	0.010
		5hr	23.683	0.321	0.103	0.131	23.347	24.020	0.014
		24hr	25.689	0.601	0.361	0.245	25.059	26.320	0.023
		48hr	24.120	0.381	0.145	0.156	23.720	24.520	0.016
	6 Gy	5min	23.436	0.597	0.357	0.244	22.810	24.063	0.025
		60min	23.480	0.456	0.208	0.186	23.001	23.959	0.019
		5hr	23.745	0.525	0.276	0.214	23.194	24.296	0.022
		24hr	25.188	1.242	1.543	0.507	23.884	26.492	0.049
		48hr	24.045	0.125	0.016	0.051	23.913	24.176	0.005
UBC	2 Gy	5min	20.862	0.170	0.029	0.070	20.683	21.040	0.008
		60min	20.988	0.565	0.319	0.231	20.395	21.580	0.027
		5hr	22.920	0.879	0.773	0.359	21.997	23.843	0.038
		24hr	22.277	0.846	0.715	0.345	21.390	23.164	0.038
		48hr	22.935	0.639	0.408	0.261	22.265	23.606	0.028
	4 Gy	5min	21.361	0.731	0.534	0.298	20.594	22.128	0.034
		60min	22.400	0.238	0.057	0.097	22.150	22.649	0.011
		5hr	21.017	0.177	0.031	0.072	20.831	21.202	0.008
		24hr	22.093	0.301	0.090	0.123	21.777	22.408	0.014
		48hr	22.543	0.212	0.045	0.087	22.321	22.766	0.009
	6 Gy	5min	21.969	1.619	2.621	0.661	20.270	23.668	0.074
		60min	20.845	0.083	0.007	0.034	20.757	20.932	0.004
		5hr	20.745	0.369	0.136	0.151	20.358	21.132	0.018
		24hr	21.658	0.703	0.494	0.287	20.920	22.395	0.032
		48hr	22.504	0.152	0.023	0.062	22.345	22.664	0.007

NCI-H226			Mean	Standard Deviation	Variance	SE of mean	Lower 95% CI of Mean	Upper 95% CI of Mean	Coefficient of Variation
YWHAZ	2 Gy	5min	25.702	0.524	0.274	0.214	25.152	26.251	0.020
		60min	25.711	0.874	0.764	0.357	24.794	26.629	0.034
		5hr	28.394	1.046	1.094	0.427	27.296	29.491	0.037
		24hr	25.218	1.195	1.429	0.488	23.963	26.472	0.047
		48hr	27.106	0.307	0.094	0.125	26.784	27.428	0.011
	4 Gy	5min	26.156	1.072	1.149	0.438	25.032	27.281	0.041
		60min	27.634	0.105	0.011	0.043	27.524	27.744	0.004
		5hr	25.841	0.291	0.085	0.119	25.536	26.147	0.011
		24hr	25.082	0.761	0.579	0.311	24.284	25.880	0.030
		48hr	26.461	0.508	0.258	0.207	25.928	26.994	0.019
	6 Gy	5min	27.003	2.167	4.698	0.885	24.728	29.277	0.080
		60min	25.825	0.438	0.192	0.179	25.365	26.284	0.017
		5hr	25.641	0.574	0.330	0.234	25.039	26.244	0.022
		24hr	24.540	1.134	1.286	0.463	23.350	25.730	0.046
		48hr	26.160	0.057	0.003	0.023	26.099	26.220	0.002
GAPDH	2 Gy	5min	15.968	0.552	0.304	0.225	15.389	16.547	0.035
		60min	16.194	0.839	0.703	0.342	15.314	17.074	0.052
		5hr	18.988	1.535	2.357	0.627	17.377	20.599	0.081
		24hr	19.909	2.396	5.741	0.978	17.395	22.424	0.120
		48hr	19.269	0.820	0.672	0.335	18.409	20.130	0.043
	4 Gy	5min	16.041	0.629	0.395	0.257	15.381	16.701	0.039
		60min	19.620	0.252	0.063	0.103	19.356	19.884	0.013
		5hr	16.258	0.284	0.081	0.116	15.960	16.556	0.017
		24hr	18.833	0.593	0.351	0.242	18.211	19.454	0.031
		48hr	17.537	0.187	0.035	0.076	17.340	17.733	0.011
	6 Gy	5min	17.752	2.795	7.814	1.141	14.818	20.685	0.157
		60min	15.916	0.263	0.069	0.107	15.640	16.191	0.017
		5hr	16.040	0.176	0.031	0.072	15.855	16.225	0.011
		24hr	18.115	1.015	1.030	0.414	17.050	19.180	0.056
		48hr	17.075	0.439	0.192	0.179	16.615	17.535	0.026
HPRT1	2 Gy	5min	22.503	0.525	0.276	0.214	21.952	23.054	0.023
		60min	22.237	1.080	1.167	0.441	21.103	23.371	0.049
		5hr	23.784	1.836	3.370	0.749	21.857	25.710	0.077
		24hr	24.413	1.131	1.279	0.462	23.226	25.600	0.046
		48hr	23.305	0.286	0.082	0.117	23.004	23.605	0.012
	4 Gy	5min	22.654	0.797	0.635	0.325	21.818	23.490	0.035
		60min	22.851	0.146	0.021	0.060	22.698	23.005	0.006
		5hr	22.549	0.367	0.135	0.150	22.164	22.934	0.016
		24hr	24.504	0.869	0.754	0.355	23.592	25.415	0.035
		48hr	23.828	0.452	0.204	0.184	23.353	24.302	0.019
	6 Gy	5min	22.216	0.592	0.351	0.242	21.594	22.837	0.027
		60min	22.596	0.484	0.235	0.198	22.087	23.104	0.021
		5hr	22.703	0.592	0.351	0.242	22.082	23.325	0.026
		24hr	24.453	1.463	2.139	0.597	22.918	25.988	0.060
		48hr	23.960	0.333	0.111	0.136	23.611	24.309	0.014
ACTB	2 Gy	5min	19.342	1.331	1.772	0.543	17.945	20.738	0.069
		60min	18.989	1.465	2.147	0.598	17.452	20.527	0.077
		5hr	22.988	0.402	0.161	0.164	22.566	23.409	0.017
		24hr	24.166	3.354	11.251	1.369	20.646	27.687	0.139
		48hr	25.263	0.178	0.032	0.073	25.076	25.450	0.007
	4 Gy	5min	19.702	1.702	2.897	0.695	17.916	21.488	0.086
		60min	22.797	0.457	0.209	0.186	22.318	23.276	0.020
		5hr	20.877	1.601	2.563	0.654	19.196	22.557	0.077
		24hr	24.064	2.285	5.222	0.933	21.666	26.462	0.095
		48hr	23.663	0.585	0.342	0.239	23.049	24.278	0.025
	6 Gy	5min	21.323	3.909	15.279	1.596	17.221	25.425	0.183
		60min	21.335	3.148	9.912	1.285	18.031	24.639	0.148
		5hr	20.055	1.651	2.727	0.674	18.322	21.788	0.082
		24hr	23.147	2.959	8.754	1.208	20.042	26.252	0.128
		48hr	22.945	0.617	0.380	0.252	22.298	23.593	0.027
B2M	2 Gy	5min	17.069	0.514	0.264	0.210	16.530	17.608	0.030
		60min	17.171	0.974	0.949	0.398	16.149	18.194	0.057
		5hr	18.732	2.362	5.578	0.964	16.253	21.210	0.126
		24hr	18.805	1.062	1.129	0.434	17.691	19.920	0.056
		48hr	17.861	0.199	0.040	0.081	17.652	18.070	0.011
	4 Gy	5min	17.108	0.876	0.767	0.357	16.189	18.026	0.051
		60min	17.209	0.209	0.044	0.085	16.990	17.428	0.012
		5hr	16.592	0.301	0.090	0.123	16.277	16.908	0.018
		24hr	18.716	0.689	0.474	0.281	17.993	19.439	0.037
		48hr	17.827	0.489	0.239	0.200	17.314	18.340	0.027
	6 Gy	5min	16.748	0.490	0.240	0.200	16.234	17.262	0.029
		60min	16.724	0.233	0.054	0.095	16.479	16.968	0.014
		5hr	16.120	0.748	0.560	0.306	15.334	16.905	0.046
		24hr	18.291	1.260	1.587	0.514	16.969	19.613	0.069
		48hr	17.424	0.686	0.470	0.280	16.704	18.144	0.039
TBP	2 Gy	5min	24.855	0.360	0.130	0.147	24.477	25.233	0.014
		60min	24.687	0.750	0.563	0.306	23.899	25.474	0.030
		5hr	27.172	0.673	0.453	0.275	26.466	27.879	0.025
		24hr	27.358	1.749	3.058	0.714	25.522	29.193	0.064
		48hr	26.815	0.449	0.202	0.183	26.344	27.287	0.017
	4 Gy	5min	24.802	0.495	0.245	0.202	24.282	25.321	0.020
		60min	26.966	0.148	0.022	0.060	26.811	27.121	0.005
		5hr	25.007	0.133	0.018	0.054	24.867	25.147	0.005
		24hr	26.640	0.376	0.141	0.153	26.245	27.034	0.014
		48hr	26.195	0.153	0.024	0.063	26.034	26.356	0.006
	6 Gy	5min	25.794	1.923	3.697	0.785	23.776	27.812	0.075
		60min	24.795	0.292	0.085	0.119	24.489	25.100	0.012
		5hr	24.637	0.265	0.070	0.108	24.359	24.915	0.011
		24hr	25.825	0.702	0.492	0.286	25.089	26.561	0.027
		48hr	25.875	0.074	0.006	0.030	25.797	25.953	0.003
TFRC	2 Gy	5min	21.333	0.314	0.098	0.128	21.004	21.663	0.015
		60min	21.235	0.604	0.365	0.247	20.601	21.869	0.028
		5hr	23.956	0.187	0.035	0.077	23.759	24.153	0.008
		24hr	23.162	1.640	2.689	0.669	21.441	24.882	0.071
		48hr	23.321	0.435	0.189	0.178	22.864	23.777	0.019
	4 Gy	5min	21.706	1.050	1.103	0.429	20.604	22.808	0.048
		60min	23.857	0.094	0.009	0.038	23.758	23.955	0.004
		5hr	21.129	0.191	0.037	0.078	20.929	21.330	0.009
		24hr	22.459	0.786	0.618	0.321	21.634	23.284	0.035
		48hr	22.354	0.116	0.014	0.048	22.231	22.476	0.005
	6 Gy	5min	22.108	1.755	3.080	0.716	20.266	23.950	0.079
		60min	21.201	0.412	0.169	0.168	20.769	21.632	0.019
		5hr	20.773	0.313	0.098	0.128	20.444	21.102	0.015
		24hr	21.761	1.036	1.074	0.423	20.674	22.849	0.048
		48hr	22.116	0.079	0.006	0.032	22.033	22.198	0.004

Table S12. Mean Cq values, standard deviation (SD), standard error of mean, 95% confidence intervals, variance and coefficient of variation (CV) of 14 HKGs across MIA PaCa-2 cells (pancreas cancer cell line) with various levels of radiation treatment (2 Gy, 4 Gy, and 6 Gy) and time post-irradiation (5 min, 1, 5, 24, and 48 h).

MIA PaCa-2			Mean	Standard Deviation	Variance	SE of mean	Lower 95% CI of Mean	Upper 95% CI of Mean	Coefficient of Variation
**G6PD**	2 Gy	5min	24.528	0.527	0.277	0.176	24.124	24.933	0.021
		60min	25.981	1.492	2.225	0.497	24.834	27.128	0.057
		5hr	25.033	1.837	3.374	0.612	23.621	26.445	0.073
		24hr	27.396	2.098	4.400	0.699	25.783	29.008	0.077
		48hr	25.434	1.001	1.002	0.334	24.664	26.203	0.039
	4 Gy	5min	25.279	0.420	0.176	0.140	24.957	25.602	0.017
		60min	26.610	1.328	1.764	0.443	25.589	27.631	0.050
		5hr	26.036	1.049	1.100	0.350	25.230	26.842	0.040
		24hr	27.726	1.316	1.731	0.439	26.715	28.737	0.047
		48hr	26.385	0.285	0.081	0.095	26.166	26.603	0.011
	6 Gy	5min	25.250	0.772	0.596	0.257	24.656	25.844	0.031
		60min	25.969	1.736	3.013	0.579	24.635	27.303	0.067
		5hr	25.152	1.664	2.770	0.555	23.873	26.432	0.066
		24hr	26.256	1.729	2.988	0.576	24.927	27.585	0.066
		48hr	24.939	0.933	0.870	0.311	24.222	25.656	0.037
**IPO8**	2 Gy	5min	23.315	0.900	0.809	0.300	22.623	24.006	0.039
		60min	24.403	1.050	1.103	0.350	23.595	25.210	0.043
		5hr	24.495	1.074	1.153	0.358	23.669	25.320	0.044
		24hr	25.997	2.121	4.497	0.707	24.367	27.627	0.082
		48hr	23.970	1.241	1.541	0.414	23.015	24.924	0.052
	4 Gy	5min	23.786	0.553	0.306	0.184	23.361	24.211	0.023
		60min	24.869	0.755	0.570	0.252	24.289	25.450	0.030
		5hr	24.506	0.644	0.415	0.215	24.010	25.001	0.026
		24hr	26.194	0.118	0.014	0.039	26.103	26.285	0.005
		48hr	25.782	0.604	0.365	0.201	25.318	26.246	0.023
	6 Gy	5min	24.329	1.019	1.037	0.340	23.546	25.112	0.042
		60min	25.135	0.416	0.173	0.139	24.816	25.455	0.017
		5hr	24.058	0.880	0.775	0.293	23.381	24.734	0.037
		24hr	25.382	1.555	2.419	0.518	24.187	26.578	0.061
		48hr	24.303	0.471	0.222	0.157	23.941	24.665	0.019
**PGK1**	2 Gy	5min	20.349	1.033	1.068	0.344	19.555	21.143	0.051
		60min	21.562	0.249	0.062	0.083	21.370	21.753	0.012
		5hr	20.918	0.800	0.639	0.267	20.303	21.532	0.038
		24hr	24.432	2.679	7.178	0.893	22.373	26.492	0.110
		48hr	20.566	0.949	0.900	0.316	19.836	21.295	0.046
	4 Gy	5min	20.499	0.667	0.445	0.222	19.987	21.012	0.033
		60min	21.427	0.852	0.726	0.284	20.772	22.082	0.040
		5hr	20.761	0.496	0.246	0.165	20.380	21.142	0.024
		24hr	24.154	0.988	0.976	0.329	23.395	24.914	0.041
		48hr	22.896	0.969	0.939	0.323	22.151	23.641	0.042
	6 Gy	5min	20.958	0.582	0.339	0.194	20.511	21.406	0.028
		60min	20.586	0.852	0.727	0.284	19.930	21.241	0.041
		5hr	20.463	0.977	0.954	0.326	19.712	21.213	0.048
		24hr	23.725	2.408	5.800	0.803	21.874	25.577	0.102
		48hr	20.668	0.104	0.011	0.035	20.588	20.748	0.005
**PP1A**	2 Gy	5min	17.547	0.887	0.786	0.296	16.866	18.229	0.051
		60min	17.441	0.983	0.967	0.328	16.685	18.197	0.056
		5hr	17.867	0.640	0.409	0.213	17.375	18.359	0.036
		24hr	20.317	2.621	6.868	0.874	18.303	22.332	0.129
		48hr	17.635	0.709	0.502	0.236	17.090	18.180	0.040
	4 Gy	5min	17.761	0.390	0.152	0.130	17.461	18.061	0.022
		60min	18.138	0.824	0.679	0.275	17.505	18.771	0.045
		5hr	17.602	0.741	0.548	0.247	17.033	18.172	0.042
		24hr	21.942	0.802	0.643	0.267	21.326	22.559	0.037
		48hr	21.197	1.857	3.449	0.619	19.769	22.624	0.088
	6 Gy	5min	17.944	0.611	0.373	0.204	17.475	18.414	0.034
		60min	17.738	0.472	0.223	0.157	17.376	18.101	0.027
		5hr	17.708	1.098	1.205	0.366	16.864	18.551	0.062
		24hr	20.471	2.134	4.555	0.711	18.831	22.112	0.104
		48hr	17.918	0.185	0.034	0.062	17.775	18.060	0.010
**HMBS**	2 Gy	5min	29.960	1.014	1.028	0.338	29.181	30.739	0.034
		60min	30.930	0.911	0.829	0.304	30.230	31.630	0.029
		5hr	30.481	0.401	0.161	0.134	30.173	30.789	0.013
		24hr	32.308	1.116	1.245	0.372	31.450	33.166	0.035
		48hr	30.067	0.629	0.396	0.210	29.584	30.551	0.021
	4 Gy	5min	30.116	0.720	0.518	0.240	29.563	30.670	0.024
		60min	30.827	1.001	1.001	0.334	30.058	31.596	0.032
		5hr	30.337	0.527	0.278	0.176	29.932	30.742	0.017
		24hr	32.196	0.773	0.597	0.258	31.602	32.790	0.024
		48hr	31.926	1.191	1.419	0.397	31.011	32.842	0.037
	6 Gy	5min	30.608	0.801	0.642	0.267	29.992	31.224	0.026
		60min	30.429	0.627	0.393	0.209	29.947	30.911	0.021
		5hr	29.903	0.818	0.669	0.273	29.274	30.531	0.027
		24hr	31.245	1.284	1.648	0.428	30.259	32.232	0.041
		48hr	29.573	0.205	0.042	0.068	29.416	29.731	0.007
**GUSB**	2 Gy	5min	22.632	0.651	0.423	0.217	22.131	23.132	0.029
		60min	22.747	0.561	0.315	0.187	22.316	23.178	0.025
		5hr	23.224	0.557	0.310	0.186	22.796	23.652	0.024
		24hr	25.263	1.806	3.263	0.602	23.875	26.652	0.072
		48hr	22.889	0.263	0.069	0.088	22.687	23.091	0.012
	4 Gy	5min	22.598	0.171	0.029	0.057	22.466	22.729	0.008
		60min	22.912	0.521	0.271	0.174	22.512	23.313	0.023
		5hr	22.684	0.323	0.105	0.108	22.435	22.932	0.014
		24hr	25.454	0.377	0.142	0.126	25.165	25.744	0.015
		48hr	24.746	1.378	1.898	0.459	23.687	25.805	0.056
	6 Gy	5min	23.016	0.433	0.188	0.144	22.683	23.349	0.019
		60min	22.940	0.359	0.129	0.120	22.664	23.216	0.016
		5hr	22.479	0.191	0.036	0.064	22.332	22.625	0.008
		24hr	24.427	2.247	5.049	0.749	22.700	26.154	0.092
		48hr	22.444	0.343	0.118	0.114	22.181	22.708	0.015
**UBC**	2 Gy	5min	20.740	0.727	0.529	0.242	20.181	21.299	0.035
		60min	21.308	0.432	0.187	0.144	20.975	21.640	0.020
		5hr	21.697	0.678	0.459	0.226	21.176	22.218	0.031
		24hr	22.315	0.468	0.219	0.156	21.956	22.675	0.021
		48hr	21.676	0.506	0.256	0.169	21.288	22.065	0.023
	4 Gy	5min	20.843	0.357	0.127	0.119	20.569	21.118	0.017
		60min	21.384	0.478	0.229	0.159	21.016	21.751	0.022
		5hr	21.819	0.458	0.210	0.153	21.467	22.171	0.021
		24hr	21.890	0.703	0.494	0.234	21.350	22.431	0.032
		48hr	21.815	0.845	0.714	0.282	21.165	22.464	0.039
	6 Gy	5min	21.082	0.450	0.202	0.150	20.736	21.427	0.021
		60min	21.241	0.635	0.403	0.212	20.754	21.729	0.030
		5hr	21.447	0.465	0.216	0.155	21.090	21.805	0.022
		24hr	21.972	0.511	0.262	0.171	21.579	22.365	0.023
		48hr	21.504	0.404	0.163	0.135	21.193	21.815	0.019

MIA PaCa-2			Mean	Standard Deviation	Variance	SE of mean	Lower 95% CI of Mean	Upper 95% CI of Mean	Coefficient of Variation
**YWHAZ**	2 Gy	5min	26.489	0.324	0.105	0.108	26.239	26.738	0.012
		60min	27.269	0.584	0.341	0.195	26.820	27.718	0.021
		5hr	27.008	0.833	0.694	0.278	26.367	27.648	0.031
		24hr	27.224	0.520	0.270	0.173	26.824	27.624	0.019
		48hr	27.847	1.031	1.062	0.344	27.055	28.640	0.037
	4 Gy	5min	27.186	0.367	0.135	0.122	26.903	27.468	0.014
		60min	27.857	0.461	0.213	0.154	27.502	28.211	0.017
		5hr	27.681	0.552	0.304	0.184	27.257	28.105	0.020
		24hr	27.025	0.625	0.391	0.208	26.544	27.505	0.023
		48hr	28.142	1.689	2.851	0.563	26.844	29.440	0.060
	6 Gy	5min	27.159	0.303	0.092	0.101	26.926	27.392	0.011
		60min	27.145	1.441	2.076	0.480	26.038	28.253	0.053
		5hr	26.905	0.829	0.687	0.276	26.268	27.542	0.031
		24hr	26.472	0.675	0.455	0.225	25.954	26.991	0.025
		48hr	26.516	0.631	0.398	0.210	26.031	27.001	0.024
**GAPDH**	2 Gy	5min	15.529	0.783	0.613	0.261	14.927	16.130	0.050
		60min	16.770	0.476	0.227	0.159	16.404	17.136	0.028
		5hr	16.739	1.643	2.698	0.548	15.477	18.002	0.098
		24hr	19.252	2.445	5.979	0.815	17.373	21.132	0.127
		48hr	16.521	1.255	1.574	0.418	15.557	17.486	0.076
	4 Gy	5min	16.637	0.681	0.464	0.227	16.113	17.160	0.041
		60min	17.448	0.726	0.528	0.242	16.890	18.006	0.042
		5hr	16.944	0.818	0.668	0.273	16.315	17.572	0.048
		24hr	19.556	0.563	0.317	0.188	19.123	19.989	0.029
		48hr	18.765	1.062	1.128	0.354	17.949	19.582	0.057
	6 Gy	5min	16.635	0.542	0.294	0.181	16.218	17.051	0.033
		60min	16.276	1.322	1.747	0.441	15.260	17.292	0.081
		5hr	15.830	0.851	0.724	0.284	15.176	16.484	0.054
		24hr	17.561	1.791	3.209	0.597	16.184	18.938	0.102
		48hr	15.915	0.492	0.242	0.164	15.536	16.293	0.031
**HPRT1**	2 Gy	5min	19.657	0.566	0.320	0.189	19.222	20.092	0.029
		60min	20.446	0.643	0.413	0.214	19.952	20.940	0.031
		5hr	20.713	0.944	0.891	0.315	19.987	21.438	0.046
		24hr	22.940	2.344	5.493	0.781	21.138	24.741	0.102
		48hr	20.806	1.023	1.046	0.341	20.020	21.592	0.049
	4 Gy	5min	20.176	0.186	0.035	0.062	20.033	20.319	0.009
		60min	20.365	0.152	0.023	0.051	20.248	20.481	0.007
		5hr	20.438	0.529	0.280	0.176	20.032	20.845	0.026
		24hr	24.000	0.346	0.120	0.115	23.734	24.267	0.014
		48hr	23.694	1.315	1.730	0.438	22.683	24.705	0.056
	6 Gy	5min	20.486	0.370	0.137	0.123	20.201	20.770	0.018
		60min	20.285	0.366	0.134	0.122	20.004	20.567	0.018
		5hr	20.203	0.573	0.328	0.191	19.763	20.644	0.028
		24hr	22.594	1.992	3.968	0.664	21.063	24.126	0.088
		48hr	20.693	0.543	0.295	0.181	20.275	21.110	0.026
**ACTB**	2 Gy	5min	21.323	1.400	1.960	0.467	20.247	22.399	0.066
		60min	22.429	2.086	4.353	0.695	20.826	24.033	0.093
		5hr	21.195	1.238	1.534	0.413	20.243	22.147	0.058
		24hr	23.591	2.439	5.948	0.813	21.716	25.466	0.103
		48hr	20.942	1.340	1.795	0.447	19.913	21.972	0.064
	4 Gy	5min	21.297	1.525	2.325	0.508	20.125	22.469	0.072
		60min	21.786	1.385	1.917	0.462	20.722	22.850	0.064
		5hr	21.656	0.895	0.802	0.298	20.967	22.344	0.041
		24hr	24.010	1.402	1.964	0.467	22.933	25.088	0.058
		48hr	23.172	0.669	0.447	0.223	22.658	23.686	0.029
	6 Gy	5min	21.294	0.944	0.891	0.315	20.569	22.020	0.044
		60min	20.997	1.520	2.311	0.507	19.828	22.165	0.072
		5hr	20.844	1.192	1.421	0.397	19.928	21.760	0.057
		24hr	23.125	2.879	8.286	0.960	20.912	25.337	0.124
		48hr	21.115	1.482	2.197	0.494	19.976	22.255	0.070
**B2M**	2 Gy	5min	18.992	0.581	0.338	0.194	18.545	19.438	0.031
		60min	19.532	0.266	0.071	0.089	19.328	19.737	0.014
		5hr	18.968	0.347	0.121	0.116	18.701	19.235	0.018
		24hr	21.389	2.116	4.476	0.705	19.762	23.015	0.099
		48hr	18.963	0.403	0.163	0.134	18.653	19.273	0.021
	4 Gy	5min	18.881	0.283	0.080	0.094	18.663	19.098	0.015
		60min	19.231	0.683	0.467	0.228	18.706	19.756	0.036
		5hr	19.100	0.232	0.054	0.077	18.922	19.279	0.012
		24hr	21.948	0.187	0.035	0.062	21.805	22.092	0.009
		48hr	21.003	1.411	1.992	0.470	19.918	22.088	0.067
	6 Gy	5min	19.123	0.407	0.166	0.136	18.810	19.436	0.021
		60min	19.521	0.373	0.139	0.124	19.234	19.808	0.019
		5hr	18.939	0.441	0.194	0.147	18.600	19.278	0.023
		24hr	21.274	1.979	3.917	0.660	19.753	22.795	0.093
		48hr	18.192	0.392	0.153	0.131	17.891	18.493	0.022
**TBP**	2 Gy	5min	24.609	0.452	0.204	0.151	24.262	24.956	0.018
		60min	25.897	0.599	0.358	0.200	25.437	26.357	0.023
		5hr	25.192	0.792	0.627	0.264	24.583	25.801	0.031
		24hr	26.539	1.232	1.518	0.411	25.592	27.486	0.046
		48hr	25.583	1.007	1.013	0.336	24.809	26.357	0.039
	4 Gy	5min	25.031	0.449	0.202	0.150	24.686	25.377	0.018
		60min	25.995	0.786	0.618	0.262	25.390	26.599	0.030
		5hr	25.720	0.409	0.167	0.136	25.406	26.035	0.016
		24hr	26.807	0.493	0.243	0.164	26.429	27.186	0.018
		48hr	26.332	0.583	0.339	0.194	25.885	26.780	0.022
	6 Gy	5min	25.251	0.385	0.148	0.128	24.955	25.546	0.015
		60min	25.409	1.004	1.008	0.335	24.637	26.181	0.040
		5hr	24.873	0.963	0.927	0.321	24.133	25.614	0.039
		24hr	26.037	1.269	1.610	0.423	25.062	27.013	0.049
		48hr	24.723	0.574	0.329	0.191	24.282	25.164	0.023
**TFRC**	2 Gy	5min	21.725	0.451	0.203	0.150	21.379	22.072	0.021
		60min	22.839	0.812	0.659	0.271	22.215	23.463	0.036
		5hr	22.487	0.971	0.942	0.324	21.741	23.233	0.043
		24hr	24.036	1.515	2.295	0.505	22.871	25.200	0.063
		48hr	23.066	0.866	0.749	0.289	22.400	23.731	0.038
	4 Gy	5min	22.137	1.107	1.226	0.369	21.286	22.988	0.050
		60min	23.362	0.454	0.206	0.151	23.013	23.711	0.019
		5hr	23.351	0.307	0.094	0.102	23.116	23.587	0.013
		24hr	24.758	0.854	0.729	0.285	24.102	25.414	0.034
		48hr	24.022	0.652	0.425	0.217	23.521	24.523	0.027
	6 Gy	5min	22.183	0.699	0.488	0.233	21.646	22.720	0.032
		60min	22.647	1.205	1.453	0.402	21.721	23.574	0.053
		5hr	21.765	1.185	1.404	0.395	20.855	22.676	0.054
		24hr	23.637	1.612	2.598	0.537	22.398	24.876	0.068
		48hr	22.318	1.034	1.070	0.345	21.523	23.113	0.046

Table S13. Mean Cq values, standard deviation (SD), standard error of mean, 95% confidence intervals, variance and coefficient of variation (CV) of 14 HKGs across PANC-1 cells (pancreas cancer cell line) with various levels of radiation treatment (2 Gy, 4 Gy, and 6 Gy) and time post-irradiation (5 min, 1, 5, 24, and 48 h).

PANC-1			Mean	Standard Deviation	Variance	SE of mean	Lower 95% CI of Mean	Upper 95% CI of Mean	Coefficient of Variation
**G6PD**	2 Gy	5min	23.823	3.937	15.499	1.312	20.797	26.849	0.165
		60min	23.299	4.287	18.381	1.429	20.003	26.594	0.184
		5hr	24.688	2.877	8.278	0.959	22.477	26.900	0.117
		24hr	23.323	1.063	1.131	0.355	22.505	24.140	0.046
		48hr	24.745	0.691	0.478	0.230	24.213	25.276	0.028
	4 Gy	5min	22.286	2.227	4.962	0.742	20.574	23.998	0.100
		60min	22.275	2.500	6.250	0.833	20.353	24.196	0.112
		5hr	22.266	1.368	1.871	0.456	21.215	23.317	0.061
		24hr	23.180	1.854	3.439	0.618	21.755	24.605	0.080
		48hr	24.614	1.201	1.441	0.400	23.691	25.537	0.049
	6 Gy	5min	21.923	2.036	4.145	0.679	20.358	23.488	0.093
		60min	21.074	1.561	2.437	0.520	19.874	22.274	0.074
		5hr	21.892	1.458	2.124	0.486	20.771	23.012	0.067
		24hr	23.452	1.176	1.383	0.392	22.548	24.356	0.050
		48hr	23.180	0.812	0.659	0.271	22.556	23.804	0.035
**IPO8**	2 Gy	5min	23.824	1.240	1.538	0.413	22.871	24.777	0.052
		60min	23.349	1.047	1.097	0.349	22.544	24.154	0.045
		5hr	23.700	0.771	0.595	0.257	23.108	24.293	0.033
		24hr	24.448	0.265	0.070	0.088	24.244	24.651	0.011
		48hr	25.673	1.368	1.872	0.456	24.621	26.724	0.053
	4 Gy	5min	24.077	1.512	2.288	0.504	22.915	25.240	0.063
		60min	24.232	1.985	3.939	0.662	22.706	25.757	0.082
		5hr	24.249	1.023	1.046	0.341	23.463	25.035	0.042
		24hr	25.221	1.518	2.304	0.506	24.054	26.387	0.060
		48hr	25.627	1.048	1.098	0.349	24.822	26.432	0.041
	6 Gy	5min	23.635	1.564	2.447	0.521	22.433	24.838	0.066
		60min	23.182	0.998	0.995	0.333	22.415	23.948	0.043
		5hr	23.803	1.244	1.548	0.415	22.846	24.759	0.052
		24hr	25.153	1.182	1.398	0.394	24.244	26.062	0.047
		48hr	24.851	0.557	0.311	0.186	24.422	25.279	0.022
**PGK1**	2 Gy	5min	20.107	1.722	2.964	0.574	18.783	21.430	0.086
		60min	19.374	1.178	1.389	0.393	18.468	20.280	0.061
		5hr	19.855	0.717	0.514	0.239	19.304	20.406	0.036
		24hr	21.038	0.571	0.326	0.190	20.600	21.477	0.027
		48hr	22.742	1.876	3.520	0.625	21.300	24.184	0.083
	4 Gy	5min	19.792	1.537	2.361	0.512	18.611	20.973	0.078
		60min	20.414	2.220	4.930	0.740	18.707	22.120	0.109
		5hr	20.376	0.674	0.454	0.225	19.858	20.894	0.033
		24hr	21.408	2.010	4.040	0.670	19.863	22.953	0.094
		48hr	22.578	0.992	0.984	0.331	21.815	23.340	0.044
	6 Gy	5min	19.518	1.585	2.512	0.528	18.300	20.736	0.081
		60min	18.677	0.957	0.915	0.319	17.942	19.412	0.051
		5hr	19.706	0.930	0.865	0.310	18.991	20.421	0.047
		24hr	21.988	1.287	1.656	0.429	20.999	22.978	0.059
		48hr	20.677	1.529	2.339	0.510	19.501	21.853	0.074
**PP1A**	2 Gy	5min	17.019	0.476	0.227	0.159	16.653	17.385	0.028
		60min	16.659	0.469	0.220	0.156	16.299	17.019	0.028
		5hr	16.750	0.346	0.120	0.115	16.484	17.016	0.021
		24hr	18.240	1.005	1.009	0.335	17.468	19.013	0.055
		48hr	19.330	1.778	3.163	0.593	17.963	20.697	0.092
	4 Gy	5min	16.782	0.582	0.338	0.194	16.335	17.230	0.035
		60min	16.813	0.900	0.810	0.300	16.121	17.505	0.054
		5hr	17.225	0.472	0.223	0.157	16.862	17.588	0.027
		24hr	18.025	0.873	0.762	0.291	17.354	18.696	0.048
		48hr	18.866	0.956	0.914	0.319	18.131	19.601	0.051
	6 Gy	5min	16.480	0.498	0.248	0.166	16.098	16.863	0.030
		60min	16.654	0.575	0.330	0.192	16.212	17.096	0.035
		5hr	16.990	0.617	0.381	0.206	16.516	17.465	0.036
		24hr	18.578	0.935	0.874	0.312	17.859	19.297	0.050
		48hr	18.735	1.216	1.480	0.405	17.800	19.670	0.065
**HMBS**	2 Gy	5min	30.168	1.685	2.838	0.562	28.873	31.463	0.056
		60min	29.337	0.625	0.391	0.208	28.857	29.818	0.021
		5hr	29.303	0.480	0.230	0.160	28.934	29.672	0.016
		24hr	28.621	0.595	0.354	0.198	28.164	29.078	0.021
		48hr	30.766	1.110	1.232	0.370	29.913	31.619	0.036
	4 Gy	5min	29.182	0.813	0.661	0.271	28.557	29.807	0.028
		60min	30.102	1.578	2.489	0.526	28.890	31.315	0.052
		5hr	29.320	0.582	0.339	0.194	28.873	29.768	0.020
		24hr	30.417	2.216	4.912	0.739	28.714	32.121	0.073
		48hr	30.453	0.316	0.100	0.105	30.210	30.696	0.010
	6 Gy	5min	29.218	1.082	1.171	0.361	28.387	30.050	0.037
		60min	28.759	0.699	0.489	0.233	28.222	29.297	0.024
		5hr	29.016	0.849	0.720	0.283	28.363	29.668	0.029
		24hr	29.668	0.454	0.206	0.151	29.319	30.017	0.015
		48hr	29.426	0.630	0.397	0.210	28.942	29.910	0.021
**GUSB**	2 Gy	5min	23.233	0.562	0.316	0.187	22.801	23.665	0.024
		60min	22.745	0.403	0.163	0.134	22.435	23.055	0.018
		5hr	22.530	0.175	0.031	0.058	22.395	22.664	0.008
		24hr	23.610	0.510	0.260	0.170	23.218	24.002	0.022
		48hr	24.283	0.648	0.420	0.216	23.785	24.782	0.027
	4 Gy	5min	22.713	0.576	0.332	0.192	22.270	23.156	0.025
		60min	22.885	0.656	0.430	0.219	22.381	23.389	0.029
		5hr	22.821	0.471	0.222	0.157	22.459	23.183	0.021
		24hr	23.897	0.887	0.786	0.296	23.216	24.579	0.037
		48hr	24.628	0.518	0.269	0.173	24.230	25.027	0.021
	6 Gy	5min	22.923	0.479	0.230	0.160	22.554	23.291	0.021
		60min	22.431	0.371	0.138	0.124	22.146	22.716	0.017
		5hr	22.651	0.347	0.120	0.116	22.384	22.917	0.015
		24hr	24.195	0.181	0.033	0.060	24.056	24.334	0.007
		48hr	23.553	0.144	0.021	0.048	23.443	23.664	0.006
**UBC**	2 Gy	5min	22.419	1.042	1.086	0.347	21.618	23.220	0.046
		60min	21.078	0.884	0.781	0.295	20.399	21.757	0.042
		5hr	21.374	0.606	0.367	0.202	20.908	21.840	0.028
		24hr	21.609	0.473	0.224	0.158	21.245	21.973	0.022
		48hr	23.066	1.168	1.365	0.389	22.167	23.964	0.051
	4 Gy	5min	20.882	1.113	1.238	0.371	20.027	21.738	0.053
		60min	21.769	1.376	1.894	0.459	20.711	22.827	0.063
		5hr	21.572	1.390	1.932	0.463	20.504	22.641	0.064
		24hr	23.100	1.605	2.575	0.535	21.867	24.334	0.069
		48hr	23.463	0.813	0.661	0.271	22.838	24.088	0.035
	6 Gy	5min	21.172	0.859	0.738	0.286	20.511	21.832	0.041
		60min	20.657	0.318	0.101	0.106	20.413	20.901	0.015
		5hr	20.904	0.382	0.146	0.127	20.611	21.197	0.018
		24hr	22.538	1.127	1.270	0.376	21.671	23.404	0.050
		48hr	22.383	0.917	0.840	0.306	21.678	23.088	0.041

PANC-1			Mean	Standard Deviation	Variance	SE of mean	Lower 95% CI of Mean	Upper 95% CI of Mean	Coefficient of Variation
**YWHAZ**	2 Gy	5min	26.374	1.560	2.434	0.520	25.175	27.574	0.059
		60min	25.400	0.697	0.486	0.232	24.864	25.936	0.027
		5hr	26.237	1.307	1.708	0.436	25.233	27.242	0.050
		24hr	24.988	0.275	0.076	0.092	24.776	25.200	0.011
		48hr	25.779	1.073	1.152	0.358	24.954	26.604	0.042
	4 Gy	5min	25.990	1.431	2.047	0.477	24.890	27.090	0.055
		60min	26.364	1.471	2.164	0.490	25.233	27.495	0.056
		5hr	25.963	0.796	0.634	0.265	25.351	26.575	0.031
		24hr	27.039	1.702	2.896	0.567	25.731	28.347	0.063
		48hr	25.907	0.449	0.202	0.150	25.561	26.252	0.017
	6 Gy	5min	25.800	1.053	1.109	0.351	24.990	26.609	0.041
		60min	25.625	0.755	0.571	0.252	25.045	26.206	0.029
		5hr	25.730	0.657	0.431	0.219	25.225	26.235	0.026
		24hr	26.065	0.885	0.783	0.295	25.385	26.745	0.034
		48hr	26.608	0.550	0.303	0.183	26.185	27.031	0.021
**GAPDH**	2 Gy	5min	17.801	1.310	1.716	0.437	16.794	18.808	0.074
		60min	16.839	1.007	1.014	0.336	16.065	17.613	0.060
		5hr	16.837	0.505	0.255	0.168	16.449	17.225	0.030
		24hr	17.999	0.530	0.281	0.177	17.591	18.406	0.029
		48hr	20.540	2.407	5.796	0.802	18.690	22.391	0.117
	4 Gy	5min	16.777	0.762	0.580	0.254	16.191	17.362	0.045
		60min	17.617	1.270	1.614	0.423	16.640	18.593	0.072
		5hr	17.275	0.253	0.064	0.084	17.081	17.469	0.015
		24hr	18.610	2.073	4.295	0.691	17.017	20.203	0.111
		48hr	18.920	0.791	0.626	0.264	18.312	19.528	0.042
	6 Gy	5min	16.563	0.578	0.334	0.193	16.119	17.007	0.035
		60min	17.028	1.173	1.375	0.391	16.127	17.929	0.069
		5hr	17.133	0.457	0.208	0.152	16.782	17.484	0.027
		24hr	19.365	2.255	5.083	0.752	17.632	21.098	0.116
		48hr	18.620	1.133	1.283	0.378	17.749	19.490	0.061
**HPRT1**	2 Gy	5min	21.114	0.983	0.965	0.328	20.358	21.869	0.047
		60min	20.855	0.543	0.295	0.181	20.438	21.272	0.026
		5hr	20.172	0.487	0.237	0.162	19.798	20.546	0.024
		24hr	20.640	1.032	1.064	0.344	19.847	21.433	0.050
		48hr	21.462	1.247	1.554	0.416	20.503	22.420	0.058
	4 Gy	5min	20.583	0.147	0.021	0.049	20.471	20.696	0.007
		60min	20.667	0.754	0.568	0.251	20.087	21.246	0.036
		5hr	20.343	0.231	0.053	0.077	20.165	20.520	0.011
		24hr	20.912	0.518	0.269	0.173	20.513	21.310	0.025
		48hr	21.742	1.578	2.491	0.526	20.529	22.955	0.073
	6 Gy	5min	20.415	0.319	0.102	0.106	20.170	20.660	0.016
		60min	20.311	0.396	0.157	0.132	20.007	20.616	0.020
		5hr	20.633	0.454	0.206	0.151	20.284	20.982	0.022
		24hr	21.230	0.851	0.724	0.284	20.576	21.884	0.040
		48hr	21.462	1.018	1.036	0.339	20.679	22.244	0.047
**ACTB**	2 Gy	5min	20.971	2.215	4.905	0.738	19.269	22.674	0.106
		60min	20.379	1.169	1.368	0.390	19.480	21.278	0.057
		5hr	20.437	0.994	0.988	0.331	19.672	21.201	0.049
		24hr	22.767	1.320	1.742	0.440	21.753	23.782	0.058
		48hr	24.029	2.351	5.525	0.784	22.222	25.836	0.098
	4 Gy	5min	21.257	2.310	5.335	0.770	19.482	23.033	0.109
		60min	21.239	2.193	4.808	0.731	19.554	22.925	0.103
		5hr	21.395	1.800	3.239	0.600	20.011	22.778	0.084
		24hr	23.659	1.849	3.419	0.616	22.238	25.081	0.078
		48hr	23.726	1.129	1.276	0.376	22.858	24.594	0.048
	6 Gy	5min	20.898	2.018	4.074	0.673	19.347	22.450	0.097
		60min	20.852	1.876	3.519	0.625	19.410	22.294	0.090
		5hr	21.353	1.927	3.715	0.642	19.871	22.834	0.090
		24hr	23.812	1.835	3.368	0.612	22.401	25.223	0.077
		48hr	22.620	1.715	2.940	0.572	21.302	23.938	0.076
**B2M**	2 Gy	5min	17.972	0.540	0.291	0.180	17.557	18.387	0.030
		60min	17.567	0.260	0.068	0.087	17.368	17.767	0.015
		5hr	17.782	0.636	0.404	0.212	17.294	18.271	0.036
		24hr	18.524	0.750	0.563	0.250	17.948	19.101	0.041
		48hr	19.078	1.273	1.620	0.424	18.100	20.056	0.067
	4 Gy	5min	17.582	0.247	0.061	0.082	17.392	17.772	0.014
		60min	17.772	0.492	0.243	0.164	17.393	18.150	0.028
		5hr	17.885	0.252	0.064	0.084	17.692	18.079	0.014
		24hr	18.589	0.842	0.709	0.281	17.942	19.237	0.045
		48hr	18.895	0.887	0.787	0.296	18.213	19.576	0.047
	6 Gy	5min	17.887	0.540	0.292	0.180	17.472	18.302	0.030
		60min	18.020	0.578	0.334	0.193	17.576	18.465	0.032
		5hr	18.309	0.941	0.885	0.314	17.586	19.032	0.051
		24hr	18.745	0.608	0.369	0.203	18.278	19.212	0.032
		48hr	18.326	1.109	1.230	0.370	17.473	19.178	0.061
**TBP**	2 Gy	5min	24.988	1.503	2.259	0.501	23.833	26.144	0.060
		60min	23.676	0.755	0.570	0.252	23.095	24.256	0.032
		5hr	24.077	0.693	0.480	0.231	23.545	24.609	0.029
		24hr	24.259	0.179	0.032	0.060	24.121	24.397	0.007
		48hr	25.957	0.720	0.518	0.240	25.404	26.510	0.028
	4 Gy	5min	23.919	0.853	0.728	0.284	23.263	24.575	0.036
		60min	24.625	1.417	2.007	0.472	23.536	25.714	0.058
		5hr	24.308	0.971	0.942	0.324	23.562	25.054	0.040
		24hr	25.865	1.528	2.334	0.509	24.690	27.039	0.059
		48hr	26.102	0.722	0.521	0.241	25.547	26.656	0.028
	6 Gy	5min	23.976	0.908	0.825	0.303	23.278	24.675	0.038
		60min	23.643	0.665	0.442	0.222	23.132	24.154	0.028
		5hr	24.282	0.968	0.936	0.323	23.538	25.025	0.040
		24hr	25.660	0.853	0.728	0.284	25.004	26.316	0.033
		48hr	25.007	0.432	0.187	0.144	24.674	25.339	0.017
**TFRC**	2 Gy	5min	22.428	1.738	3.022	0.579	21.091	23.764	0.078
		60min	20.833	0.729	0.531	0.243	20.273	21.394	0.035
		5hr	21.318	0.953	0.908	0.318	20.585	22.050	0.045
		24hr	21.212	0.185	0.034	0.062	21.069	21.354	0.009
		48hr	23.161	1.625	2.641	0.542	21.912	24.411	0.070
	4 Gy	5min	21.127	1.009	1.019	0.336	20.352	21.903	0.048
		60min	21.941	1.574	2.476	0.525	20.732	23.151	0.072
		5hr	21.693	1.367	1.868	0.456	20.642	22.743	0.063
		24hr	23.422	1.600	2.559	0.533	22.192	24.651	0.068
		48hr	23.500	1.120	1.254	0.373	22.639	24.360	0.048
	6 Gy	5min	20.945	0.959	0.920	0.320	20.208	21.683	0.046
		60min	20.902	0.611	0.374	0.204	20.432	21.372	0.029
		5hr	21.601	1.135	1.289	0.378	20.728	22.473	0.053
		24hr	23.245	0.898	0.806	0.299	22.555	23.935	0.039
		48hr	22.388	0.488	0.238	0.163	22.013	22.763	0.022

Table S14. Mean Cq values, standard deviation (SD), standard error of mean, 95% confidence interval (CI) of mean, variance and coefficient of variation (CV) of 14 housekeeping genes (HKGs) across all untreated cells (0 Gy).

Untreated	HKG	Mean	Standard Deviation	SE of mean	Lower 95% CI of Mean	Upper 95% CI of Mean	Variance	Coefficient of Variation
**SCC-6**	G6PD	25.997	1.262	0.421	25.027	26.967	1.592	0.049
	IPO8	24.533	1.052	0.351	23.724	25.342	1.107	0.043
	PGK1	21.131	1.171	0.390	20.231	22.030	1.370	0.055
	PP1A	16.963	0.550	0.183	16.540	17.386	0.303	0.032
	HMBS	31.405	1.385	0.462	30.340	32.470	1.919	0.044
	GUSB	23.746	0.352	0.117	23.476	24.016	0.124	0.015
	UBC	21.117	0.926	0.309	20.405	21.829	0.857	0.044
	YWHAZ	25.088	1.141	0.380	24.210	25.965	1.303	0.045
	GAPDH	18.220	0.812	0.271	17.595	18.844	0.660	0.045
	HPRT1	20.971	0.338	0.113	20.712	21.231	0.114	0.016
	ACTB	20.932	1.420	0.473	19.840	22.023	2.016	0.068
	B2M	17.929	0.633	0.211	17.443	18.416	0.400	0.035
	TBP	24.479	0.515	0.172	24.083	24.875	0.265	0.021
	TFRC	21.903	1.027	0.342	21.113	22.692	1.055	0.047
**SCC-1483**	G6PD	24.043	2.173	0.724	22.373	25.714	4.720	0.090
	IPO8	24.374	1.510	0.503	23.213	25.534	2.280	0.062
	PGK1	20.403	1.393	0.464	19.332	21.474	1.942	0.068
	PP1A	16.827	0.643	0.214	16.333	17.321	0.413	0.038
	HMBS	29.116	1.343	0.448	28.083	30.148	1.804	0.046
	GUSB	22.716	0.646	0.215	22.219	23.212	0.418	0.028
	UBC	20.528	1.289	0.430	19.537	21.520	1.663	0.063
	YWHAZ	24.484	0.381	0.127	24.190	24.777	0.145	0.016
	GAPDH	16.546	1.265	0.422	15.573	17.518	1.601	0.076
	HPRT1	21.122	0.885	0.295	20.442	21.802	0.783	0.042
	ACTB	20.011	1.907	0.636	18.546	21.477	3.636	0.095
	B2M	19.017	0.262	0.087	18.815	19.219	0.069	0.014
	TBP	24.018	0.968	0.323	23.273	24.762	0.937	0.040
	TFRC	21.137	1.116	0.372	20.279	21.995	1.246	0.053
**A549**	G6PD	23.039	0.662	0.221	22.531	23.548	0.438	0.029
	IPO8	24.918	0.603	0.201	24.454	25.381	0.364	0.024
	PGK1	20.640	0.896	0.299	19.951	21.329	0.804	0.043
	PP1A	17.687	0.938	0.313	16.966	18.407	0.879	0.053
	HMBS	29.553	0.554	0.185	29.127	29.979	0.307	0.019
	GUSB	23.716	0.398	0.133	23.409	24.022	0.159	0.017
	UBC	22.528	0.435	0.145	22.194	22.862	0.189	0.019
	YWHAZ	26.246	0.344	0.115	25.981	26.510	0.119	0.013
	GAPDH	17.351	0.853	0.284	16.695	18.007	0.728	0.049
	HPRT1	21.167	0.146	0.049	21.055	21.280	0.021	0.007
	ACTB	22.497	1.111	0.370	21.644	23.351	1.234	0.049
	B2M	19.370	0.899	0.300	18.680	20.061	0.808	0.046
	TBP	26.257	0.709	0.236	25.712	26.802	0.503	0.027
	TFRC	23.772	1.057	0.352	22.960	24.585	1.118	0.044
**NCI-H226**	G6PD	26.774	0.711	0.290	26.027	27.520	0.506	0.027
	IPO8	26.184	0.152	0.062	26.025	26.344	0.023	0.006
	PGK1	22.854	0.791	0.323	22.024	23.684	0.625	0.035
	PP1A	19.086	0.818	0.334	18.228	19.945	0.670	0.043
	HMBS	32.247	0.272	0.111	31.961	32.532	0.074	0.008
	GUSB	24.488	0.438	0.179	24.029	24.947	0.191	0.018
	UBC	23.225	0.067	0.027	23.155	23.295	0.005	0.003
	YWHAZ	28.118	0.289	0.118	27.815	28.421	0.084	0.010
	GAPDH	20.281	1.144	0.467	19.081	21.481	1.308	0.056
	HPRT1	22.968	0.330	0.135	22.622	23.314	0.109	0.014
	ACTB	23.261	0.428	0.175	22.812	23.711	0.184	0.018
	B2M	17.862	0.199	0.081	17.653	18.071	0.040	0.011
	TBP	26.686	0.096	0.039	26.585	26.787	0.009	0.004
	TFRC	23.641	0.328	0.134	23.297	23.985	0.107	0.014
**MIA PaCa-2**	G6PD	25.526	0.254	0.085	25.331	25.721	0.065	0.010
	IPO8	23.911	0.483	0.161	23.540	24.282	0.233	0.020
	PGK1	20.707	0.790	0.263	20.100	21.314	0.623	0.038
	PP1A	17.115	0.877	0.292	16.441	17.790	0.769	0.051
	HMBS	29.867	0.572	0.191	29.427	30.307	0.328	0.019
	GUSB	22.640	0.456	0.152	22.290	22.990	0.208	0.020
	UBC	21.326	0.449	0.150	20.981	21.671	0.201	0.021
	YWHAZ	27.425	0.970	0.323	26.680	28.171	0.940	0.035
	GAPDH	15.704	0.933	0.311	14.987	16.422	0.871	0.059
	HPRT1	20.718	0.629	0.210	20.235	21.201	0.395	0.030
	ACTB	20.957	1.100	0.367	20.112	21.803	1.210	0.053
	B2M	19.468	0.452	0.151	19.120	19.815	0.204	0.023
	TBP	24.925	0.394	0.131	24.622	25.228	0.156	0.016
	TFRC	22.212	0.892	0.297	21.526	22.897	0.795	0.040
**PANC-1**	G6PD	21.617	1.290	0.430	20.625	22.609	1.665	0.060
	IPO8	23.239	0.690	0.230	22.709	23.769	0.476	0.030
	PGK1	19.247	0.614	0.205	18.775	19.718	0.376	0.032
	PP1A	16.674	1.137	0.379	15.800	17.548	1.293	0.068
	HMBS	29.007	0.613	0.204	28.536	29.478	0.376	0.021
	GUSB	22.669	0.271	0.090	22.461	22.877	0.073	0.012
	UBC	20.818	0.579	0.193	20.373	21.263	0.335	0.028
	YWHAZ	25.115	0.692	0.231	24.583	25.647	0.478	0.028
	GAPDH	16.553	0.662	0.221	16.044	17.062	0.438	0.040
	HPRT1	20.717	0.597	0.199	20.258	21.176	0.357	0.029
	ACTB	20.751	1.295	0.432	19.755	21.747	1.678	0.062
	B2M	17.924	0.224	0.075	17.752	18.096	0.050	0.012
	TBP	23.615	0.380	0.127	23.323	23.908	0.144	0.016
	TFRC	21.124	0.390	0.130	20.824	21.424	0.152	0.018

Table S15. geNorm and Normfinder results for all three cancer cell types (head and neck, lung, and pancreas). All four radiation doses (including 0 Gy) and five time points of the two cell lines from the same tissue are grouped together as one to obtain the results as shown below. The genes in the table were sorted by M value or SD from lowest (indicating the most stable HKG) to the highest (most unstable HKG).

**Head & Neck**				**Lung**				**Pancreas**			
**geNorm**		**Normfinder**		**geNorm**		**Normfinder**		**geNorm**		**Normfinder**	
**Gene**	**M-Value**	**Gene**	**SD**	**Gene**	**M-Value**	**Gene**	**SD**	**Gene**	**M-Value**	**Gene**	**SD**
UBC	0.703	IPO8	0.453	IPO8	0.784	TBP	0.438	TBP	0.491	TBP	0.440
TBP	0.703	UBC	0.499	TBP	0.784	IPO8	0.521	TFRC	0.491	IPO8	0.495
IPO8	0.729	TBP	0.505	UBC	0.873	GUSB	0.764	IPO8	0.672	TFRC	0.561
TFRC	0.786	GAPDH	0.633	GUSB	0.922	UBC	0.774	HMBS	0.773	GUSB	0.673
GAPDH	0.821	TFRC	0.651	HMBS	0.990	PGK1	0.781	GUSB	0.842	HMBS	0.701
HMBS	0.839	GUSB	0.771	PGK1	1.025	HMBS	0.820	B2M	0.898	PGK1	0.795
PGK1	0.880	HMBS	0.772	GAPDH	1.056	GAPDH	0.866	PGK1	0.944	B2M	0.808
GUSB	0.919	PP1A	0.799	PP1A	1.101	PP1A	1.016	PP1A	0.979	PP1A	0.908
PP1A	0.954	PGK1	0.811	YWHAZ	1.144	HPRT1	1.089	HPRT1	1.007	UBC	0.913
YWHAZ	0.982	YWHAZ	0.873	HPRT1	1.187	YWHAZ	1.116	UBC	1.039	HPRT1	0.956
HPRT1	1.010	HPRT1	0.964	TFRC	1.229	TFRC	1.158	GAPDH	1.091	YWHAZ	1.146
B2M	1.045	B2M	1.188	B2M	1.276	B2M	1.391	YWHAZ	1.137	GAPDH	1.160
G6PD	1.116	G6PD	1.337	ACTB	1.388	ACTB	1.848	ACTB	1.190	ACTB	1.269
ACTB	1.182	ACTB	1.422	G6PD	1.483	G6PD	1.891	G6PD	1.292	G6PD	1.797

Table S16. geNorm and Normfinder results for all cell lines. Data from four radiation doses (including 0 Gy) and five time points of each cell line are treated as one group to obtain the results as shown below. The genes in the table were sorted by M value or SD from lowest (indicating the most stable HKG) to the highest (most unstable HKG).

**SCC-6**				**SCC-1483**			
**geNorm**		**Normfinder**		**geNorm**		**Normfinder**	
**Gene**	**M-Value**	**Gene**	**SD**	**Gene**	**M-Value**	**Gene**	**SD**
UBC	0.455	TFRC	0.494	HMBS	0.535	TBP	0.275
YWHAZ	0.455	UBC	0.496	TBP	0.535	UBC	0.386
IPO8	0.683	IPO8	0.518	IPO8	0.549	IPO8	0.421
TFRC	0.735	GAPDH	0.610	UBC	0.563	HMBS	0.530
TBP	0.777	TBP	0.642	GAPDH	0.606	PGK1	0.565
GUSB	0.830	PP1A	0.669	PGK1	0.631	TFRC	0.583
PP1A	0.874	YWHAZ	0.695	TFRC	0.664	GAPDH	0.633
B2M	0.903	HMBS	0.763	GUSB	0.720	GUSB	0.657
HPRT1	0.916	HPRT1	0.849	PP1A	0.767	PP1A	0.752
GAPDH	0.934	GUSB	0.868	B2M	0.798	HPRT1	0.768
HMBS	0.951	B2M	0.898	HPRT1	0.818	B2M	0.784
PGK1	0.995	PGK1	0.957	YWHAZ	0.849	YWHAZ	0.857
G6PD	1.033	G6PD	1.045	G6PD	0.887	G6PD	0.936
ACTB	1.116	ACTB	1.510	ACTB	0.921	ACTB	0.974

**A549**				**NCI-H226**			
**geNorm**		**Normfinder**		**geNorm**		**Normfinder**	
**Gene**	**M-Value**	**Gene**	**SD**	**Gene**	**M-Value**	**Gene**	**SD**
TBP	0.660	TBP	0.429	IPO8	0.446	TBP	0.231
TFRC	0.660	IPO8	0.658	TBP	0.446	IPO8	0.326
HPRT1	0.840	HPRT1	0.702	TFRC	0.473	TFRC	0.486
GUSB	0.907	TFRC	0.710	UBC	0.517	UBC	0.492
IPO8	0.929	HMBS	0.716	B2M	0.665	G6PD	0.650
B2M	0.960	GUSB	0.782	GUSB	0.732	PGK1	0.672
PGK1	0.996	PGK1	0.790	HPRT1	0.768	B2M	0.742
HMBS	1.014	GAPDH	0.877	G6PD	0.802	GUSB	0.778
GAPDH	1.035	B2M	0.901	PGK1	0.838	HPRT1	0.857
UBC	1.066	UBC	0.904	PP1A	0.881	GAPDH	0.887
G6PD	1.093	G6PD	0.951	GAPDH	0.924	HMBS	0.967
PP1A	1.119	PP1A	1.042	HMBS	0.963	PP1A	0.979
YWHAZ	1.150	YWHAZ	1.099	YWHAZ	1.014	YWHAZ	1.176
ACTB	1.269	ACTB	1.918	ACTB	1.126	ACTB	1.742

**MIA PaCa-2**				**PANC-1**			
**geNorm**		**Normfinder**		**geNorm**		**Normfinder**	
**Gene**	**M-Value**	**Gene**	**SD**	**Gene**	**M-Value**	**Gene**	**SD**
TBP	0.507	TBP	0.490	TBP	0.475	TBP	0.402
TFRC	0.507	IPO8	0.495	TFRC	0.475	IPO8	0.451
IPO8	0.620	GUSB	0.561	UBC	0.600	UBC	0.496
HMBS	0.722	TFRC	0.571	IPO8	0.667	TFRC	0.555
GUSB	0.778	HMBS	0.635	GUSB	0.722	GUSB	0.608
B2M	0.821	GAPDH	0.707	HMBS	0.774	B2M	0.725
GAPDH	0.864	B2M	0.710	B2M	0.813	HMBS	0.730
PGK1	0.895	HPRT1	0.810	PP1A	0.858	PGK1	0.762
HPRT1	0.916	PGK1	0.813	PGK1	0.896	PP1A	0.805
PP1A	0.943	G6PD	0.848	HPRT1	0.937	GAPDH	0.937
G6PD	0.973	UBC	0.907	YWHAZ	0.972	YWHAZ	0.970
UBC	1.005	PP1A	0.997	GAPDH	1.007	HPRT1	1.049
ACTB	1.059	ACTB	1.174	ACTB	1.066	ACTB	1.239
YWHAZ	1.109	YWHAZ	1.251	G6PD	1.177	G6PD	1.776

Table S17. Normfinder results for all cell lines. The SDs for each housekeeping gene in each cell and each condition were estimated by using the Normfinder algorithm. The genes in the table were sorted by SD from lowest (indicating the most stable HKG) to the highest (most unstable HKG).

	**SCC-6**		**SCC-1483**		**A549**		**NCI-H226**		**MIA PaCa-2**		**PANC-1**	
	**HKG**	**SD**	**HKG**	**SD**	**HKG**	**SD**	**HKG**	**SD**	**HKG**	**SD**	**HKG**	**SD**
**Untreated**	UBC	0.214	TBP	0.256	YWHAZ	0.148	TBP	0.053	TBP	0.170	B2M	0.100
	IPO8	0.365	UBC	0.274	HPRT1	0.175	UBC	0.053	IPO8	0.246	GUSB	0.103
	GUSB	0.445	HMBS	0.312	GUSB	0.424	B2M	0.137	UBC	0.303	UBC	0.259
	YWHAZ	0.459	GAPDH	0.379	IPO8	0.452	IPO8	0.194	HMBS	0.308	PGK1	0.396
	TBP	0.487	PGK1	0.385	UBC	0.555	HMBS	0.204	GUSB	0.351	YWHAZ	0.437
	PP1A	0.521	PP1A	0.466	TBP	0.598	YWHAZ	0.223	G6PD	0.448	TBP	0.469
	GAPDH	0.594	TFRC	0.467	HMBS	0.741	ACTB	0.341	TFRC	0.576	IPO8	0.489
	G6PD	0.652	GUSB	0.543	PGK1	0.782	TFRC	0.348	PGK1	0.591	TFRC	0.680
	TFRC	0.697	YWHAZ	0.685	B2M	0.787	GUSB	0.373	GAPDH	0.630	GAPDH	0.704
	ACTB	0.779	IPO8	0.697	GAPDH	0.801	HPRT1	0.377	PP1A	0.746	HMBS	0.781
	PGK1	0.781	ACTB	0.985	PP1A	0.826	PP1A	0.795	YWHAZ	0.783	HPRT1	0.960
	HMBS	0.831	HPRT1	0.986	G6PD	0.850	G6PD	0.826	B2M	0.803	PP1A	0.966
	HPRT1	1.011	B2M	1.183	TFRC	1.108	PGK1	0.903	HPRT1	0.984	ACTB	1.037
	B2M	1.178	G6PD	1.661	ACTB	1.288	GAPDH	1.142	ACTB	0.992	G6PD	1.261
**2 Gy**	YWHAZ	0.333	TBP	0.195	TBP	0.417	TBP	0.115	TFRC	0.408	UBC	0.345
	UBC	0.388	UBC	0.384	IPO8	0.465	IPO8	0.386	TBP	0.495	IPO8	0.381
	PP1A	0.441	IPO8	0.391	HPRT1	0.646	TFRC	0.420	IPO8	0.529	TBP	0.451
	TFRC	0.515	HMBS	0.584	GUSB	0.727	UBC	0.482	HPRT1	0.574	B2M	0.612
	GAPDH	0.538	GAPDH	0.608	TFRC	0.791	PGK1	0.621	GUSB	0.625	TFRC	0.649
	TBP	0.560	TFRC	0.662	PGK1	0.819	G6PD	0.733	B2M	0.695	GUSB	0.697
	IPO8	0.581	PGK1	0.709	UBC	0.820	HPRT1	0.735	PP1A	0.779	PP1A	0.743
	HMBS	0.630	HPRT1	0.795	HMBS	0.824	PP1A	0.778	GAPDH	0.812	PGK1	0.777
	GUSB	0.983	PP1A	0.834	PP1A	0.851	GAPDH	0.799	HMBS	0.828	HMBS	0.840
	HPRT1	1.010	GUSB	0.850	GAPDH	0.866	B2M	0.831	G6PD	0.858	HPRT1	0.935
	PGK1	1.119	YWHAZ	0.872	YWHAZ	0.950	GUSB	0.845	UBC	0.892	GAPDH	1.088
	B2M	1.172	B2M	0.891	B2M	0.960	YWHAZ	1.217	PGK1	0.929	YWHAZ	1.098
	G6PD	1.188	G6PD	1.001	G6PD	1.054	HMBS	1.364	YWHAZ	1.169	ACTB	1.353
	ACTB	1.579	ACTB	1.131	ACTB	2.504	ACTB	1.989	ACTB	1.410	G6PD	2.539
**4 Gy**	IPO8	0.266	TBP	0.226	TBP	0.414	TBP	0.246	IPO8	0.285	TBP	0.325
	GAPDH	0.342	IPO8	0.328	TFRC	0.550	IPO8	0.249	HMBS	0.499	IPO8	0.429
	TFRC	0.381	PGK1	0.381	HMBS	0.623	UBC	0.547	GUSB	0.563	UBC	0.563
	B2M	0.512	TFRC	0.437	IPO8	0.684	B2M	0.596	TBP	0.575	TFRC	0.568
	UBC	0.514	UBC	0.474	G6PD	0.713	TFRC	0.607	GAPDH	0.581	GUSB	0.607
	PGK1	0.669	HMBS	0.517	PGK1	0.791	G6PD	0.636	B2M	0.631	GAPDH	0.637
	HPRT1	0.734	GUSB	0.536	GAPDH	0.885	PGK1	0.638	PGK1	0.668	HMBS	0.640
	YWHAZ	0.746	G6PD	0.610	GUSB	0.909	HPRT1	0.653	TFRC	0.724	PGK1	0.700
	PP1A	0.764	PP1A	0.617	HPRT1	0.919	GUSB	0.672	G6PD	0.900	B2M	0.727
	HMBS	0.767	B2M	0.679	UBC	0.973	HMBS	0.684	UBC	0.967	PP1A	0.809
	TBP	0.767	GAPDH	0.800	B2M	1.031	GAPDH	0.751	HPRT1	1.010	YWHAZ	0.921
	GUSB	0.869	HPRT1	0.831	ACTB	1.204	PP1A	1.026	ACTB	1.059	ACTB	1.106
	G6PD	1.117	ACTB	0.845	PP1A	1.284	YWHAZ	1.202	PP1A	1.222	G6PD	1.112
	ACTB	1.564	YWHAZ	0.927	YWHAZ	1.392	ACTB	1.513	YWHAZ	1.399	HPRT1	1.116
**6 Gy**	TFRC	0.412	UBC	0.264	TBP	0.416	TBP	0.284	GAPDH	0.433	TFRC	0.300
	UBC	0.486	TBP	0.309	HPRT1	0.544	TFRC	0.309	TBP	0.443	TBP	0.392
	IPO8	0.557	IPO8	0.399	HMBS	0.644	IPO8	0.355	GUSB	0.521	IPO8	0.478
	TBP	0.600	GAPDH	0.489	GUSB	0.692	UBC	0.492	TFRC	0.529	UBC	0.568
	B2M	0.667	HMBS	0.516	TFRC	0.708	G6PD	0.493	IPO8	0.553	GUSB	0.578
	GAPDH	0.697	GUSB	0.532	B2M	0.744	GAPDH	0.677	HMBS	0.574	HMBS	0.663
	GUSB	0.736	PGK1	0.586	PGK1	0.759	HMBS	0.737	HPRT1	0.749	B2M	0.768
	HPRT1	0.765	TFRC	0.593	IPO8	0.759	PGK1	0.746	UBC	0.763	PGK1	0.794
	PP1A	0.784	HPRT1	0.625	UBC	0.770	B2M	0.766	B2M	0.766	PP1A	0.826
	HMBS	0.794	B2M	0.685	GAPDH	0.865	GUSB	0.784	PP1A	0.810	G6PD	0.896
	YWHAZ	0.847	PP1A	0.774	PP1A	0.905	HPRT1	0.897	G6PD	0.849	YWHAZ	0.926
	G6PD	0.860	YWHAZ	0.799	YWHAZ	0.918	PP1A	1.128	PGK1	0.870	GAPDH	1.041
	PGK1	0.933	G6PD	0.887	G6PD	1.031	YWHAZ	1.204	ACTB	1.077	HPRT1	1.048
	ACTB	1.377	ACTB	0.925	ACTB	1.500	ACTB	1.908	YWHAZ	1.209	ACTB	1.266

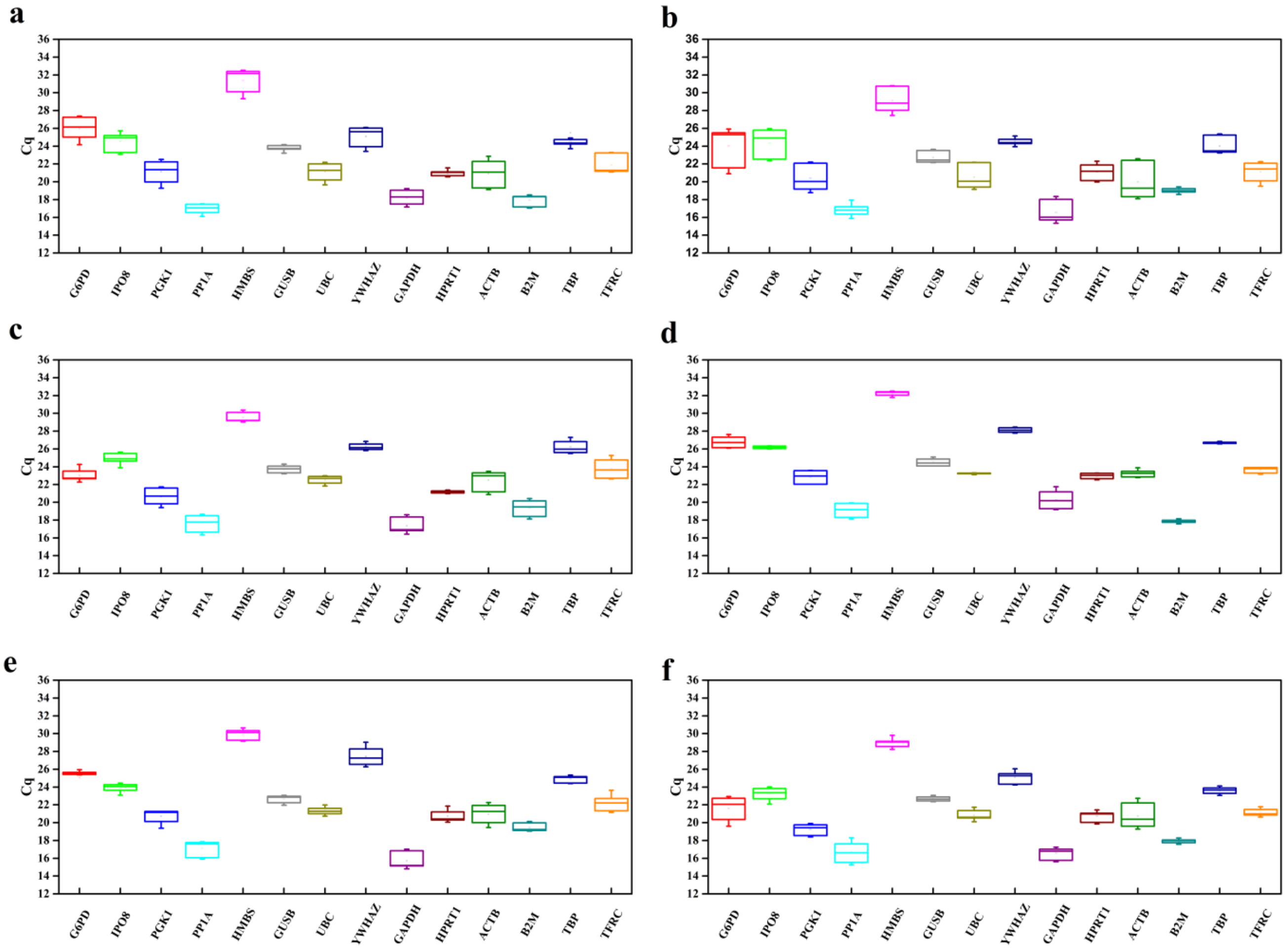

Figure S1. Gene expression (cycle threshold numbers) of untreated samples: a) SCC-6, b) SCC-1483, c) A549, d) NCI-H226, e) MIA PaCa-2, and f) PANC-1.

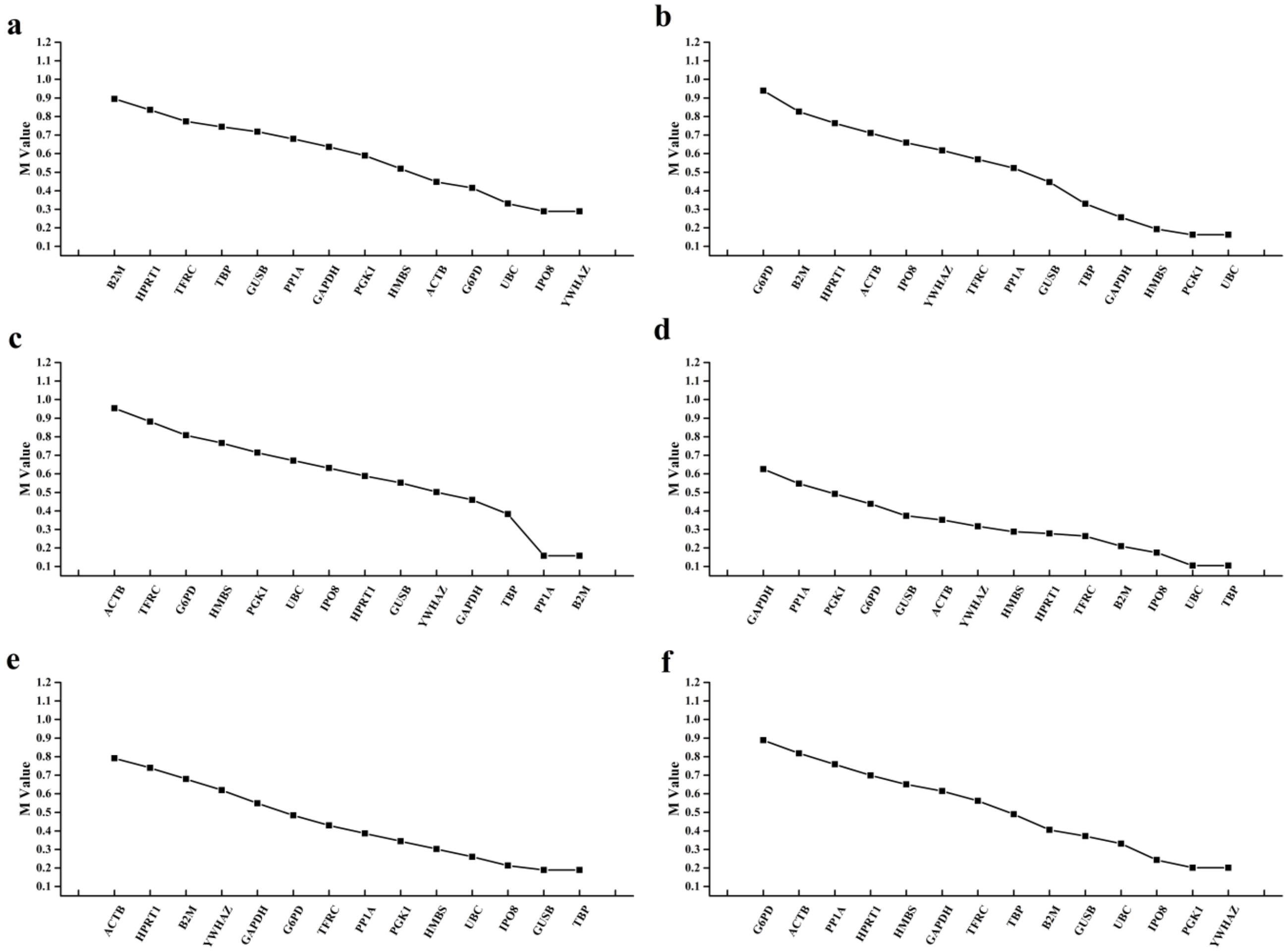

Figure S2. geNorm results for untreated samples: a) SCC-6, b) SCC-1483, c) A549, d) NCI-H226, e) MIA PaCa-2, f) PANC-1. By using the geNorm algorithm, the M values (average expression stability measure) were calculated to determine the stability of each HKG. From high to low M values, the curve represents the stepwise exclusion of the least stable HKG, meaning the most stable genes determined by geNorm are located at the end of the curve and have the lowest M values.

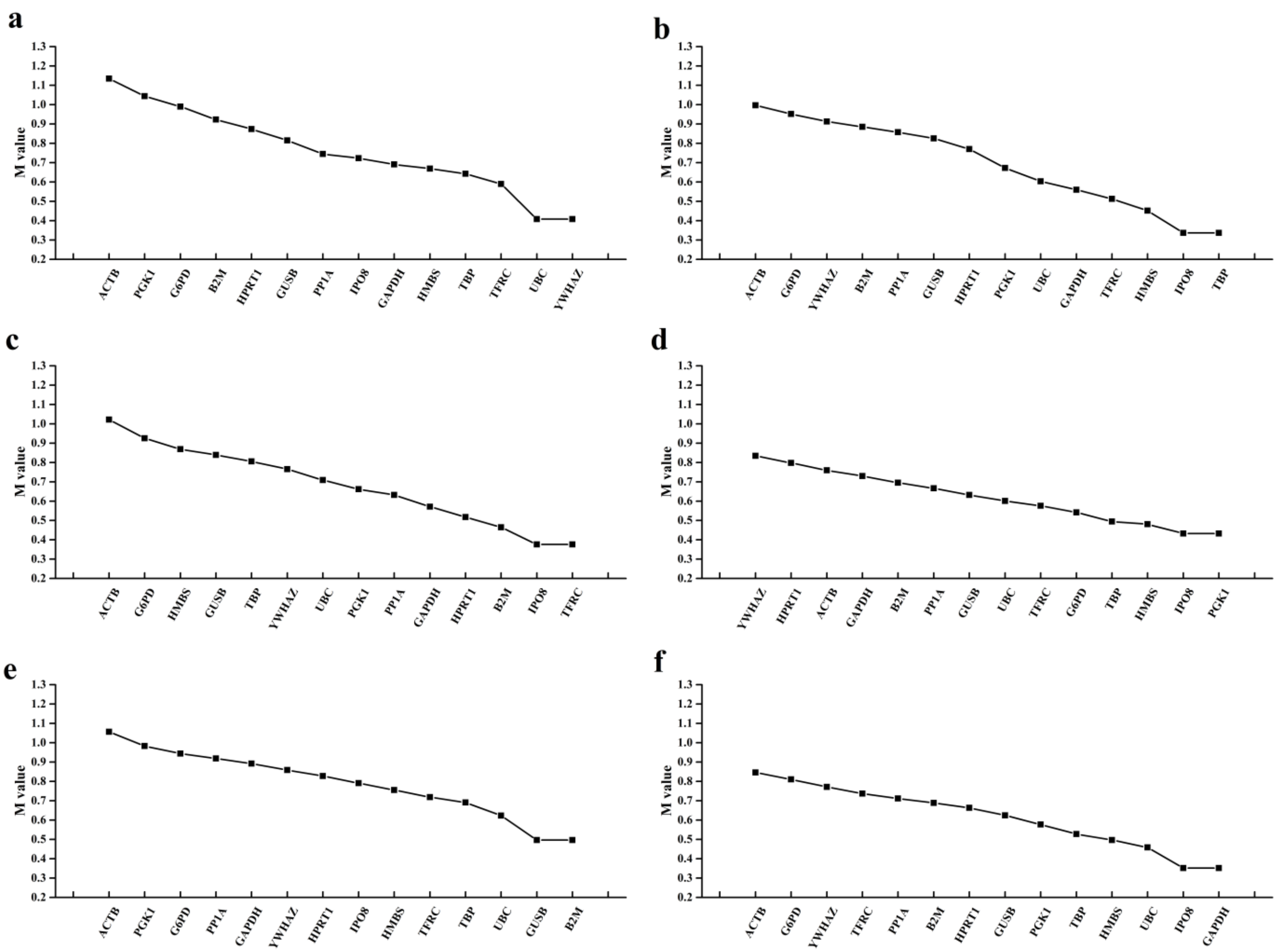

Figure S3. geNorm results for radiated SCC-6 and SCC-1483 cells. As in Figure S2, but for radiated SCC-6 and SCC-1483 cells. a) SCC-6 treated with 2 Gy radiation, b) SCC-1483 with 2 Gy radiation, c) SCC-6 with 4 Gy radiation, d) SCC-1483 with 4 Gy radiation, e) SCC-6 with 6 Gy radiation, f) SCC-1483 with 6 Gy radiation.

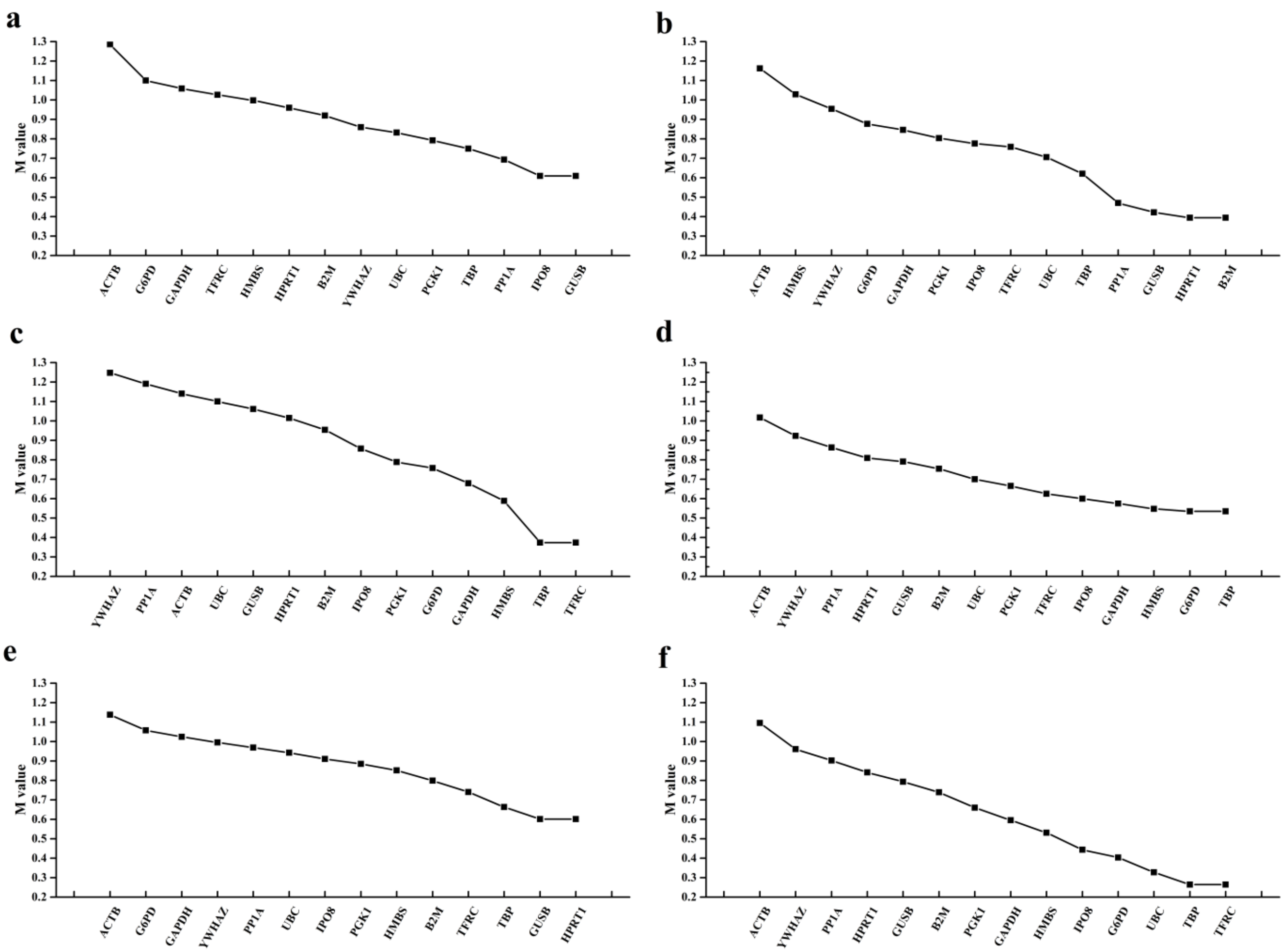

Figure S4. geNorm results for radiated A549 and NCI-H226 cells. As in Figure S3, but for radiated A549 and NCI-H226 cells. a) A549 treated with 2 Gy radiation, b) NCI-H226 with 2 Gy radiation, c) A549 with 4 Gy radiation, d) NCI-H226 with 4 Gy radiation, e) A549 with 6 Gy radiation, f) NCI-H226 with 6 Gy radiation.

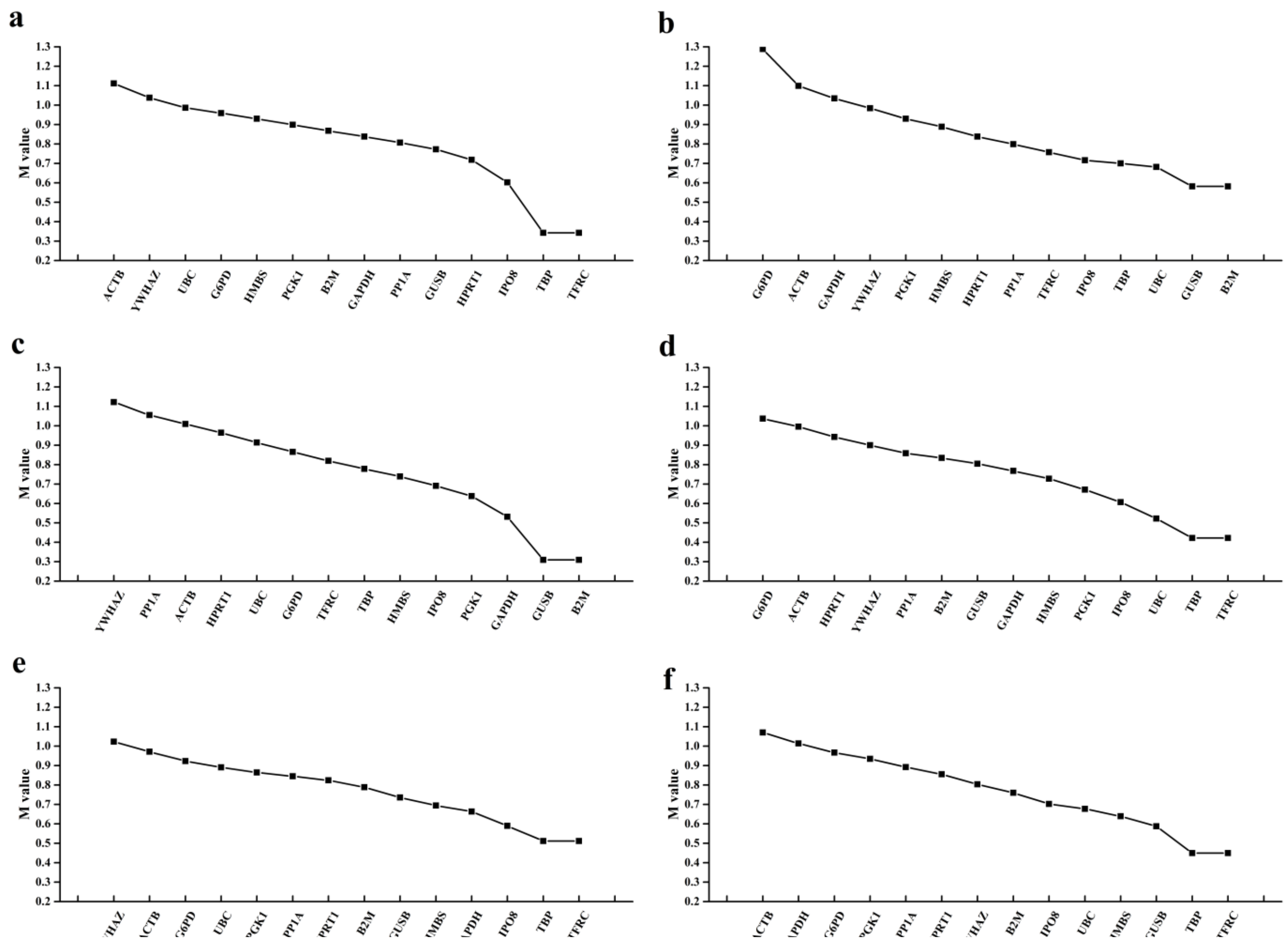

Figure S5. geNorm results for radiated MIA PaCa-2 and PANC-1. As in Figure S2, but for radiated MIA PaCa-2 and PANC-1 cells. a) MIA PaCa-2 treated with 2 Gy radiation, b) PANC-1 with 2 Gy radiation, c) MIA PaCa-2 with 4 Gy radiation, d) PANC-1 with 4 Gy radiation, e) MIA PaCa-2 with 6 Gy radiation, f) PANC-1 with 6 Gy radiation.

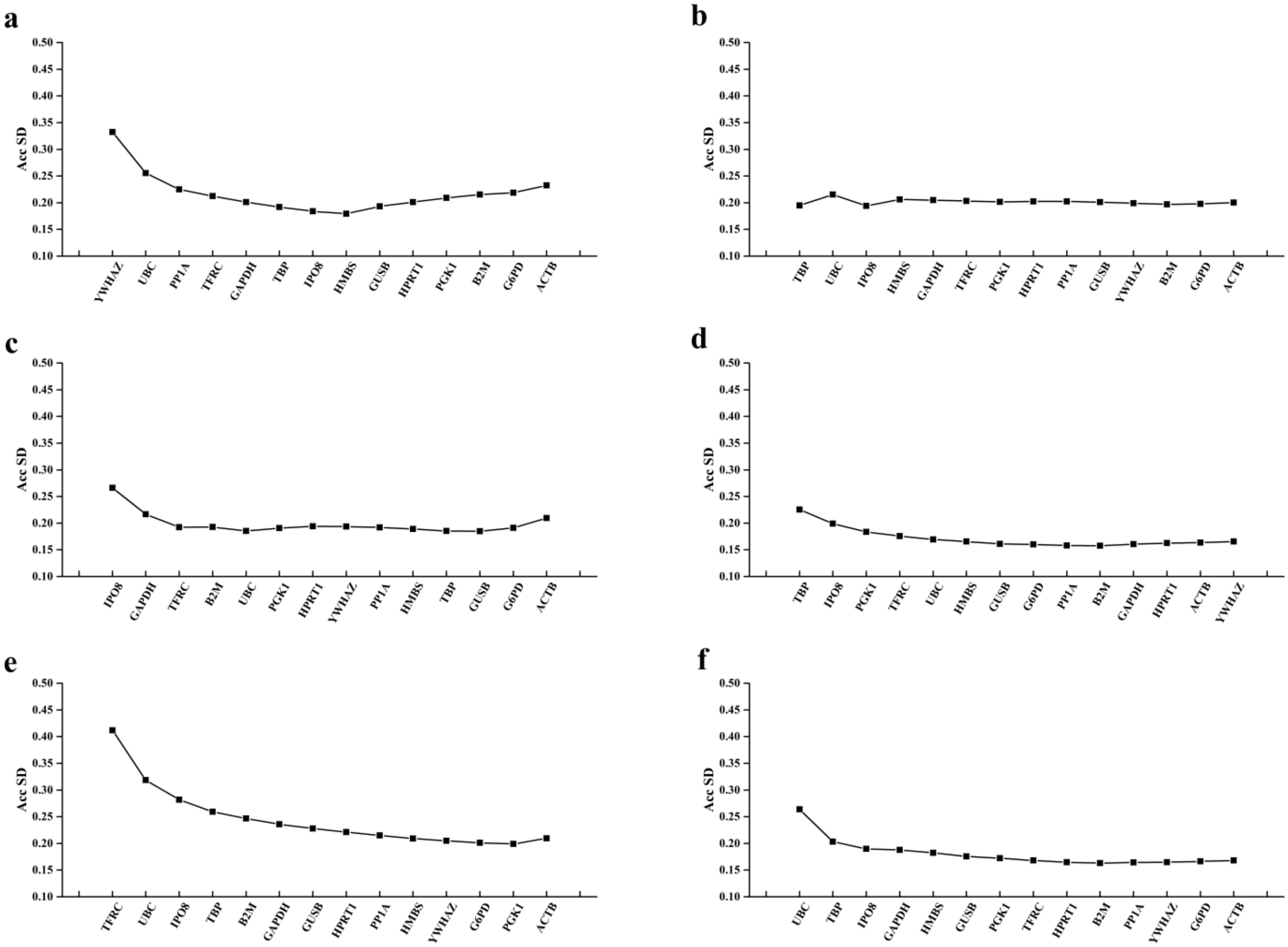

Figure S6. Accumulated SD of HKGs generated by NormFinder for SCC-6 and SCC-1483 cell lines. Accumulated SD is the SD estimated when multiple HKGs are used for normalization. Based on NormFinder, HKGs are ranked in order from most stable to most unstable starting from the left of x-axis of the plot. As more reference genes are used for normalization, the accumulated SD is decreased since the random variation among gene expression is partially canceled. a) SCC-6 treated with 2 Gy radiation, b) SCC-1483 with 2 Gy radiation, c) SCC-6 with 4 Gy radiation, d) SCC-1483 with 4 Gy radiation, e) SCC-6 with 6 Gy radiation, f) SCC-1483 with 6 Gy radiation.

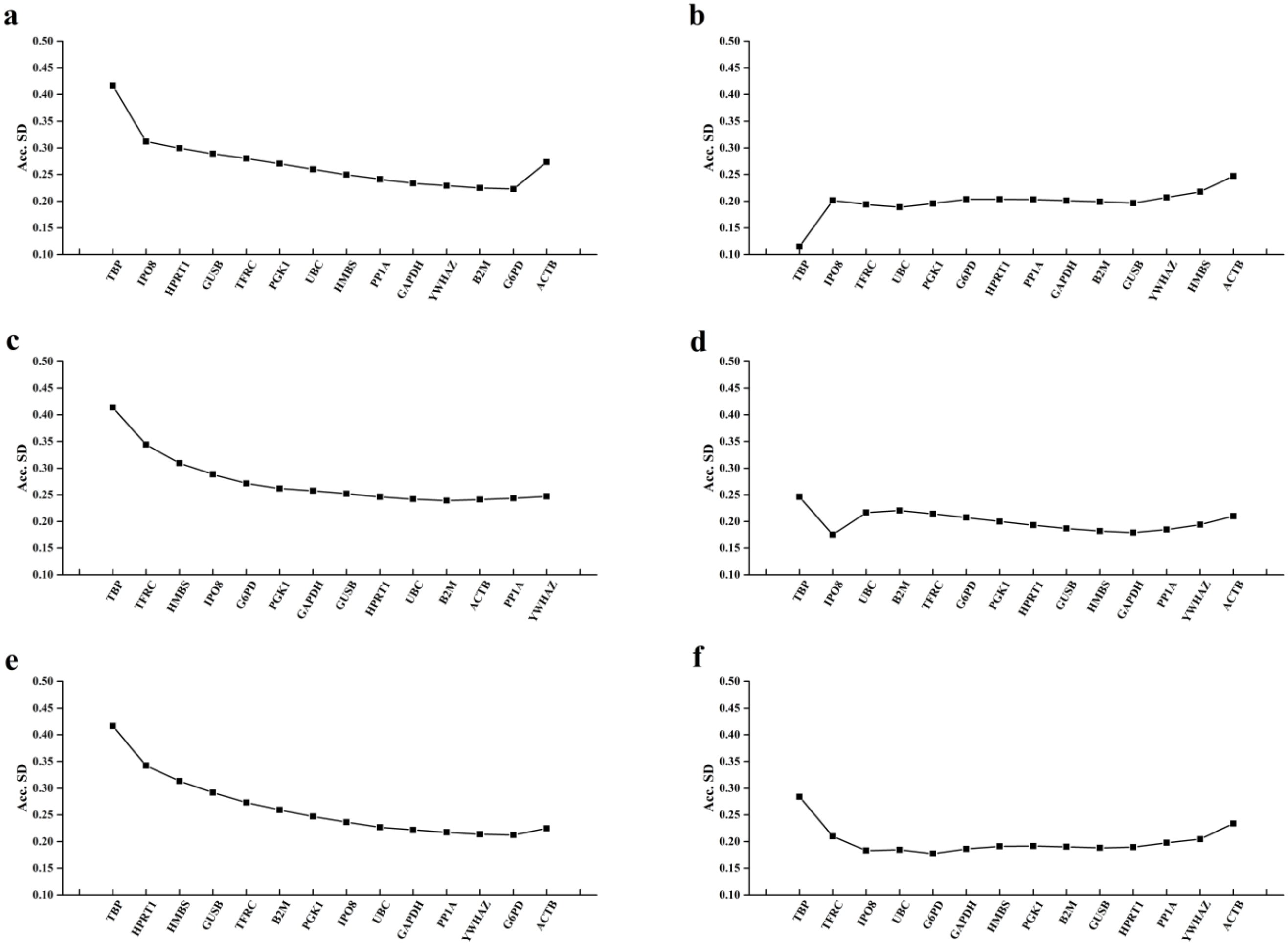

Figure S7. Accumulated SD generated by NormFinder. As in Figure S6, but for A549 and NCI-H226 cells. a) A549 treated with 2 Gy radiation, b) NCI-H226 with 2 Gy radiation, c) A549 with 4 Gy radiation, d) NCI-H226 with 4 Gy radiation, e) A549 with 6 Gy radiation, f) NCI-H226 with 6 Gy radiation.

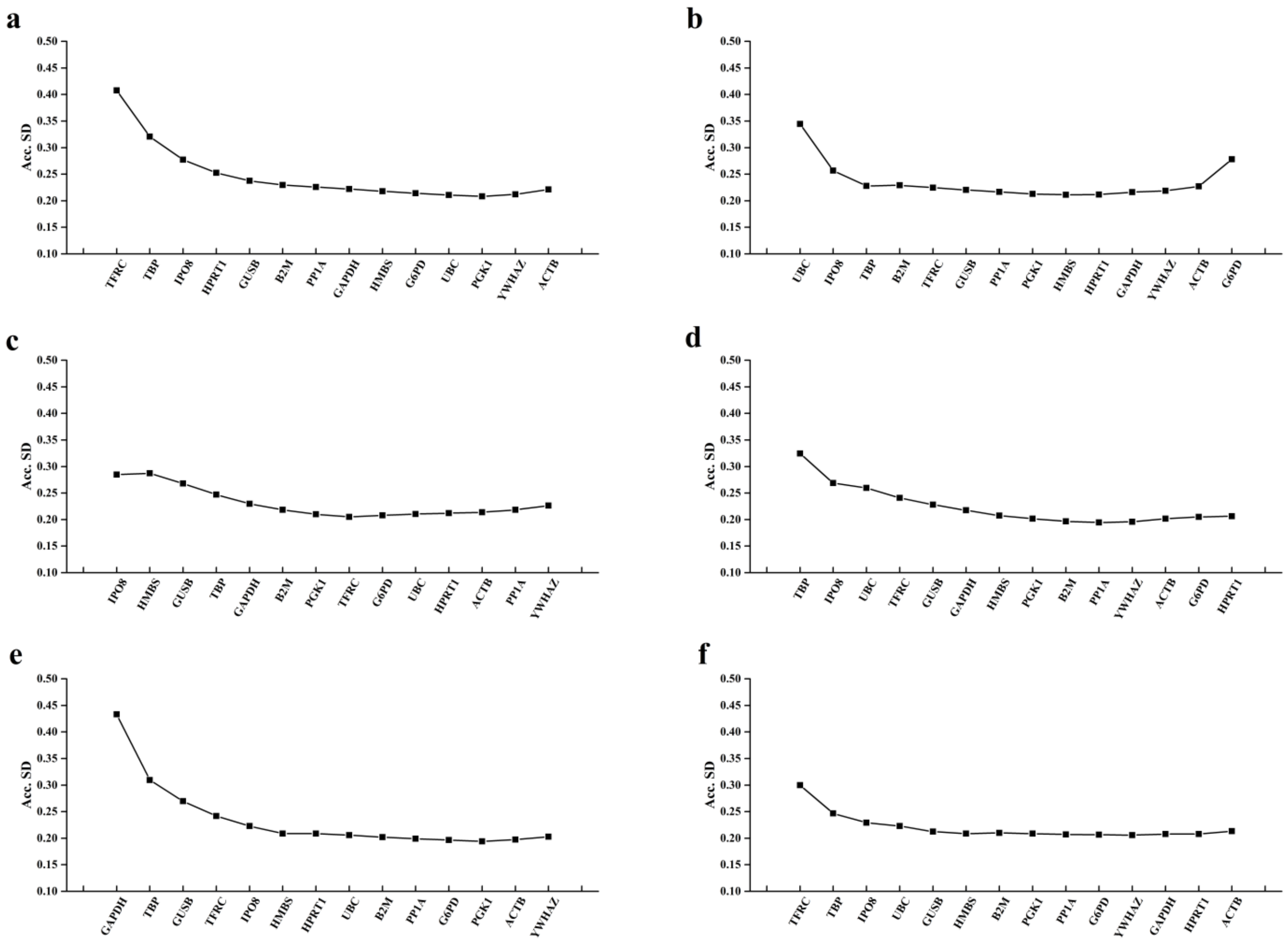

Figure S8. Accumulated SD generated by NormFinder. As in Figure S6, but for MIA PaCa-2 and PANC-1 cells. a) MIA PaCa-2 treated with 2 Gy radiation, b) PANC-1 with 2 Gy radiation, c) MIA PaCa-2 with 4 Gy radiation, d) PANC-1 with 4 Gy radiation, e) MIA PaCa-2 with 6 Gy radiation, f) PANC-1 with 6 Gy radiation.

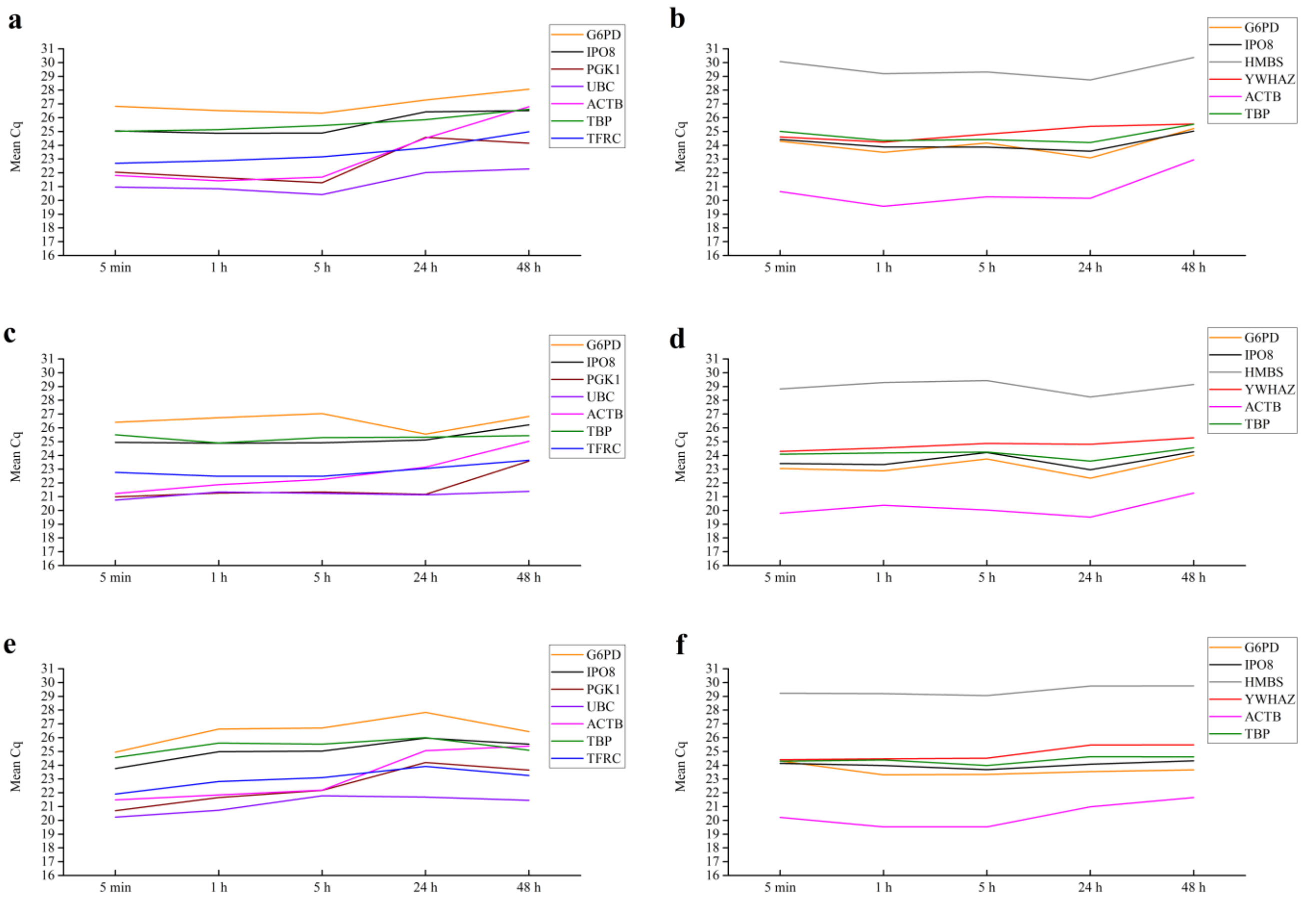

Figure S9. Mean Cq values of top three stable and unstable housekeeping genes (HKGs) for SCC-6 and SCC-1483 across different radiation treatment time (5 minutes, 1 hour, 5 hours, 24 hours, and 48 hours). a) SCC-6 treated with 2 Gy radiation, b) SCC-1483 with 2 Gy radiation, c) SCC-6 with 4 Gy radiation, d) SCC-1483 with 4 Gy radiation, e) SCC-6 with 6 Gy radiation, and f) SCC-1483 with 6 Gy radiation. In general, the expressions of stable HKGs show less fluctuation across time than the expressions of unstable HKGs. For SCC-6, the top stable HKGs are TFRC (average Cq range across all radiation times and doses: 1.82), UBC (average Cq range: 1.36), IPO8 (average Cq range: 1.74) and TBP (average Cq range: 1.21), while the unstable HKGs are ACTB (average Cq range: 4.36), G6PD (average Cq range: 2.04), and PGK1 (average Cq range: 3.12). For SCC-1483, the stable HKGs are TBP (average Cq range: 0.99), IPO8 (average Cq range: 1.13), and HMBS (average Cq range: 1.18), while the unstable HKGs are ACTB (average Cq range: 2.42), YWHAZ (average Cq range: 1.13), and G6PD (average Cq range: 1.60). Error bars are not shown due to the complexity of the figure.

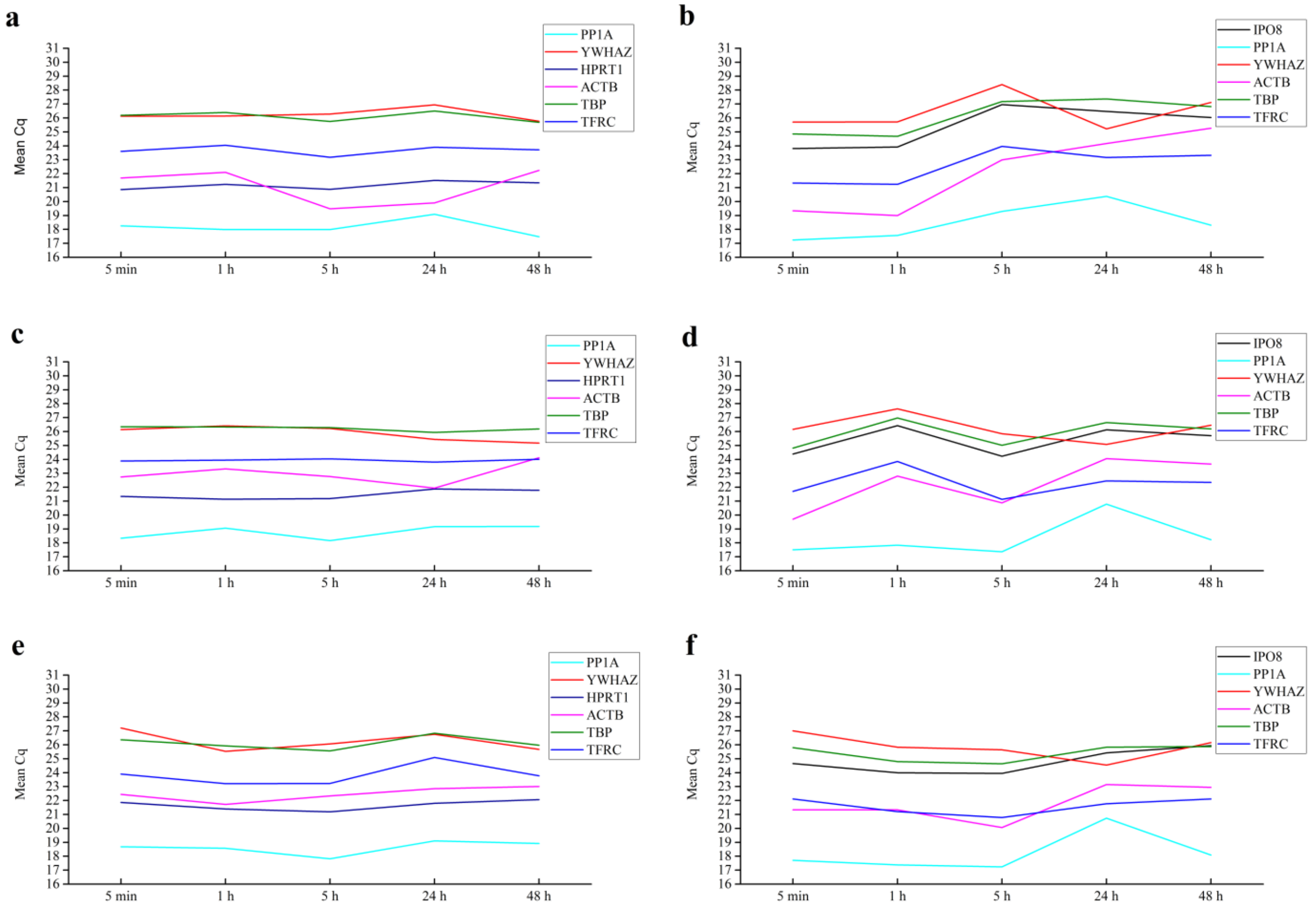

Figure S10. Mean Cq values across radiation treatment time for A549 and NCI-H226 cells. a) A549 treated with 2 Gy radiation, b) NCI-H226 with 2 Gy radiation, c) A549 with 4 Gy radiation, d) NCI-H226 with 4 Gy radiation, e) A549 with 6 Gy radiation, f) NCI-H226 with 6 Gy radiation. Top stable HKGs: TBP (average Cq range across all radiation times and doses: 0.84), TFRC (average Cq range: 1.00), and HPRT1 (average Cq range: 0.76) for A549; IPO8 (average Cq range: 2.44), TBP (average Cq range: 2.02), and TFRC (average Cq range: 2.26) for NCI-H226. Both A549 and NCI-H226 has ACTB (average Cq range: 2.07 and 4.58 respectively), YWHAZ (average Cq range: 1.36 and 2.73 respectively), and PP1A (average Cq range: 1.31 and 3.35 respectively) as their most unstable HKGs. Error bars are not shown due to the complexity of the figure.

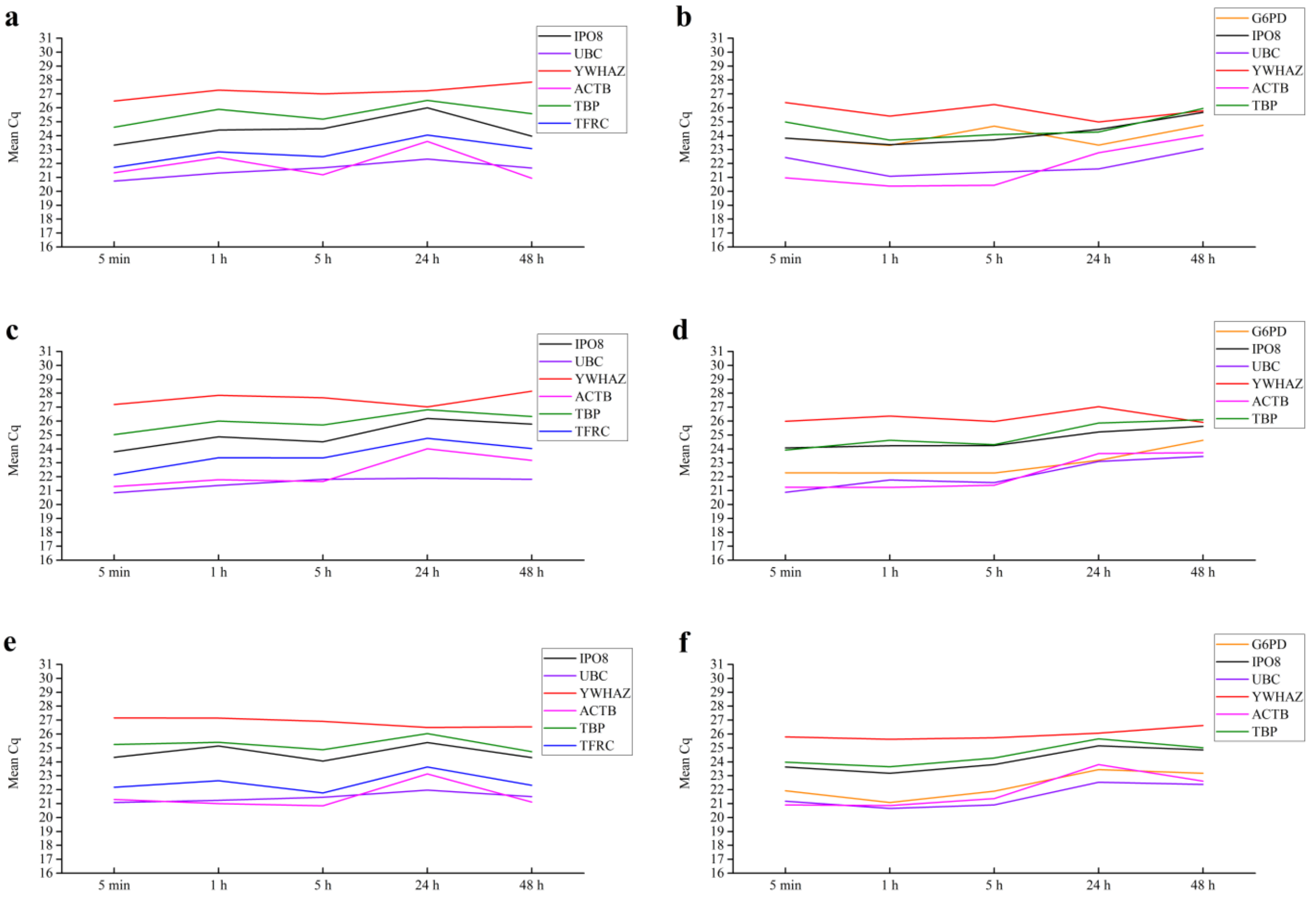

Figure S11. Mean Cq values across radiation treatment time for MIA PaCa-2 and PANC-1. a) MIA PaCa-2 treated with 2 Gy radiation, b) PANC-1 with 2 Gy radiation, c) MIA PaCa-2 with 4 Gy radiation, d) PANC-1 with 4 Gy radiation, e) MIA PaCa-2 with 6 Gy radiation, and f) PANC-1 with 6 Gy radiation. For MIA PaCa-2, the most stable HKGs are TFRC (average Cq range across all radiation times and doses: 2.27), TBP (average Cq range: 1.67), IPO8 (average Cq range: 2.14), and the most unstable HKGs are YWHAZ (average Cq range: 1.05), ACTB (average Cq range: 2.55), UBC (average Cq range: 1.17). For PANC-1, the stable HKGs are TBP (average Cq range: 2.16), UBC (average Cq range: 2.15), IPO8 (average Cq range: 1.95), while the unstable HKGs are G6PD (average Cq range: 2.06), ACTB (average Cq range: 3.03), YWHAZ (average Cq range 1.17). Error bars are not shown due to the complexity of the figure.
